# Synthesis, Characterisation and Structural Analysis of Rhenium and Technetium Nitride Complexes With Tridentate Thiosemicarbazone‐Phenols and Phosphine Ligands: Potential Applications in Technetium‐99m Radiotracer Development

**DOI:** 10.1155/bca/3202767

**Published:** 2026-07-22

**Authors:** Nicola Salvarese, Davide Lucchini, Carolina Gobbi, Marco Baron, Dominga Rogolino, Mauro Carcelli, Alessandro Dolmella, Cristina Bolzati

**Affiliations:** ^1^ Institute of Condensed Matter Chemistry and Energy Technologies–National Research Council, Corso Stati Uniti 4, Padua 35127, Italy; ^2^ Department of Chemical Sciences, University of Padua, Via F. Marzolo 1, Padua 35131, Italy, unipd.it; ^3^ Department of Chemistry, Life Sciences and Environmental Sustainability, University of Parma, Parco Area Delle Scienze 17/A, Parma 43124, Italy, unipr.it; ^4^ Department of Pharmaceutical and Pharmacological Sciences, University of Padua, Via F. Marzolo 5, Padua 35131, Italy, unipd.it

## Abstract

The reactivity of two tridentate salicylthiosemicarbazones (H_2_L1 = 3‐methoxysalicylaldehyde thiosemicarbazone; H_2_L2 = 3‐methoxysalicylaldehyde N,N‐dimethyl‐thiosemicarbazone), with the [M^V^ ≡ N]^2+^ core (M = Re, ^99m^Tc), is here described, leading to the formation of heteroleptic complexes of the general composition [MN(L)P] (P = triphenylphosphine, PPh_3_; tris(2‐cyanoethyl)phosphine, PCN). The [ReN(L)PPh_3_] complexes were prepared in good yield starting from [Re^V^NCl_2_(PPh_3_)_2_] precursor and in lower quantity from (Bu_4_N)[ReN^VI^Cl_4_]. Complexes were fully characterised by elemental analyses, spectroscopic, spectrometric techniques and X‐ray diffraction. Single‐crystal X‐ray diffraction analysis of [ReN(L1)PPh_3_] and [ReN(L2)PPh_3_] showed the formation of a distorted square base pyramid geometry with the rhenium atom located 0.57 Å above the pyramid base and the nitride ion in apical position. The basal plane is occupied by the S,N,O‐thiosemicarbazonate and the triphenylphosphine ligands. Superimposable complexes were obtained in high yield at tracer level with technetium‐99m and under carried‐added conditions. In technetium complexes, triphenylphosphine can be successfully replaced with PCN, forming corresponding complexes in high yield; this substitution was unfeasible with cold rhenium. Based on the collected preliminary results, it is reasonable to assume that [^99m^Tc][TcN(L)P] (L = bisdeprotonated ligand; P = monophosphine) could be a promising platform for developing new potential technetium‐99m‐based radiotracers. However, improvements in labelling efficiency and stability must be addressed to fully realise its potential.

## 1. Introduction

Theranostics with radionuclides represents a rapidly evolving field of research in Nuclear Medicine, and it was recently suggested as a future cancer research priority.

In clinical practice, the diagnostic part involves imaging methods that include quantitative positron emission tomography (PET) technique or, less frequently, single‐photon emission computed tomography (SPECT) and fluorine‐18 (^18^F) and gallium‐68 (^68^Ga) are the imaging radioisotopes of choice. As a therapeutic counterpart, lutetium‐177 (^177^Lu) has recently been recognised as the workhorse radionuclide for the preparation of radiotherapeutic companions. It is incorporated into two approved radiopharmaceuticals: Lutathera (^177^Lu‐DOTATATE, Novartis Pharmaceutical Corporation) and Pluvicto (^177^Lu‐PSMA‐617; Novartis Pharmaceutical Corporation) [1].

Although ^68^Ga/^177^Lu is the dominant pair of radionuclides in theranostic practices, there are important economic and infrastructural limitations related to the high costs of the PET facility and its limited availability in rural areas and developing countries, which constitutes a challenge for patients who are far from the PET centres, preventing them from undergoing PET screening for the theranostic audit trail necessary to identify suitable candidates for targeted treatments.

SPECT technique is the mainstay imaging modality in nuclear medicine, because of its high accessibility and wide clinical use; moreover, it relies mainly on technetium‐99m (^99m^Tc, γ_max_ = 140 KeV, *t*_1/2_ = 6.02 h). The latter, thanks to its ideal physical–chemical properties, low cost and convenient accessibility from the ^99^Mo‐^99m^Tc generator, is the most used radionuclide in conventional nuclear medicine.

Furthermore, the existence of two medically relevant isotopes rhenium‐186/188 [^186^Re: β_max_ = 1.07 MeV, 137 KeV *γ* (9%), *t*_1/2_ = 90 h; ^188^Re: β_max_ = 2.12 MeV, 155 KeV *γ* (15%) *t*_1/2_ = 17 h] of the heavy group VII congener, with nuclear properties suitable for targeted radionuclide therapy (TRT), opens the possibility of developing an accessible theranostic pair [2].

Being congeners of the same group, the coordination chemistry of technetium and rhenium is intrinsically connected and isostructural metal complexes of Re and Tc are frequent, which results, for stable complexes characterised by identical molecular environments, in a similar biological performance [3, 4].

The possibility of preparing ^188^Re analogues of the ^99m^Tc‐labelled tracer in a so‐called ‘theranostic pair’ can provide important therapeutic strategies, offering the opportunity to develop a tracer for widespread scintigraphic diagnostics with ^99m^Tc and for TRT with ^188^Re. This will permit a wider portion of the population to receive the best clinical care, with a positive impact on patient management, reducing the cost of health care. These considerations highlight the importance of research on the design of technetium and rhenium compounds for imaging and radiotherapy applications.

Moreover, advances in SPECT technology, in particular the introduction of cadmium–zinc–telluride (CZT) detectors [5], which allow rapid acquisition of images, higher spatial resolution and sensitivity compared to conventional Anger cameras, are changing the perspective of ^99m^Tc‐based radiopharmaceuticals, stimulating new opportunities in imaging applications of this radionuclide [6].

Because of the close chemical and structural similarities of technetium and rhenium complexes and the fact that a nonradioactive isotope of technetium does not exist, natural rhenium has often been utilised as a surrogate of Tc for nonradioactive characterisation and mechanistic studies. Likewise, ^99m^Tc radiocomplexes may serve as a prototype for the development of ^186^Re/^188^Re agents. However, the important differences in the kinetics of the ligand exchange reactions and in the redox behaviour of the two metals mean that the methods utilised for the preparation of ^188^Re radiopharmaceuticals cannot simply follow the routes employed for obtaining ^99m^Tc analogues [7], since harsher reaction conditions are required. Furthermore, rhenium complexes are thermodynamically more stable in their higher oxidation states than the corresponding ^99m^Tc compounds; therefore, once formed, ^188^Re complexes have a higher tendency to reoxidise to perrhenate than the corresponding ^99m^Tc ones, impairing the stability properties of the radiopharmaceutical.

The prerequisites for clinical translation are the safety and stability during intravenous administration of radiocompounds. Therefore, the development of a chelator for ^188^Re requires careful evaluation of the kinetic, thermodynamic and redox stability of the resulting complex to ensure high chemical inertia and stability under physiological conditions. Additionally, the selection of the most stable metal core is also crucial for the formation of strong rhenium chelates.

A successful approach in developing ^99m^Tc/^188^Re theranostic pairs exploits tetradentate ligands to stabilise the oxo‐metal [M^V^=O]^3+^ and [O = M^V^=O]^+^ cores (M = ^99m^Tc; ^188^Re). These chelators are usually N_x_S_4-x_ or N_4_ donor type [3, 8, 9].

The nitride core ([M^V^ ≡ N]^2+^) is another inorganic functional group, which is isoelectronic with the [M^V^=O]^3+^ core. Species containing the [^99m^Tc/^188^Re^V^ ≡ N]^2+^ moiety can be efficiently prepared at the tracer level under sterile and pyrogen‐free conditions [10–15]. Compared to the [M^V^=O]^3+^, the [M^V^ ≡ N]^2+^ core is highly stable in a wide range of pH values and has greater redox stability, and the nitrogen atom is more difficult to displace than oxygen, conferring to the complexes where it is present a particular thermodynamic–kinetic resistance to redox processes [4, 12]. Moreover, the nitride core is characterised by greater flexibility, more readily and frequently forming heteroleptic complexes; this characteristic expands the potential for developing labelling systems that can serve as versatile platforms for radiopharmaceutical design and development [12].

Thiosemicarbazones (TSCs) are an interesting class of ligands subject to extensive research due to their relatively high synthetic accessibility, which allows for the facile introduction of a number of different substituents that confer significant chemical and biological properties as antifungal, antibacterial and anticancer agents. Furthermore, a broad spectrum of complexes with transition and main group metals is known, and in some cases, the ligand properties are enhanced by the metalation [16–22].

Stable complexes have also been reported with technetium and rhenium [21, 23]. The coordination chemistry of technetium and rhenium studied so far involves mostly the [M^I^(CO)_3_]^+^ moiety [24–34]. With higher oxidation states (i.e., oxidation state V), fewer examples are reported, and it seems that tetradentate bis(thiosemicarbazonates) are basically preferred [23, 35–39]. In this context, our group has recently laid the foundation for the use of bis(thiosemicarbazones) as bifunctional chelators (BFCs) for the development of ^99m^TcN‐tagged biomolecules [37], demonstrating the high labelling efficiency of these ligands towards the technetium‐99m nitride core; to gain more detailed structural insights, we also successfully prepared and characterised a technetium‐99 g nitride complex. Conversely, all attempts to isolate the corresponding rhenium analogue were unsuccessful, indicating a significantly lower affinity of this chelating system for the rhenium nitride core. This reduced reactivity may limit the theranostic applicability of the platform.

**FIGURE 1:**
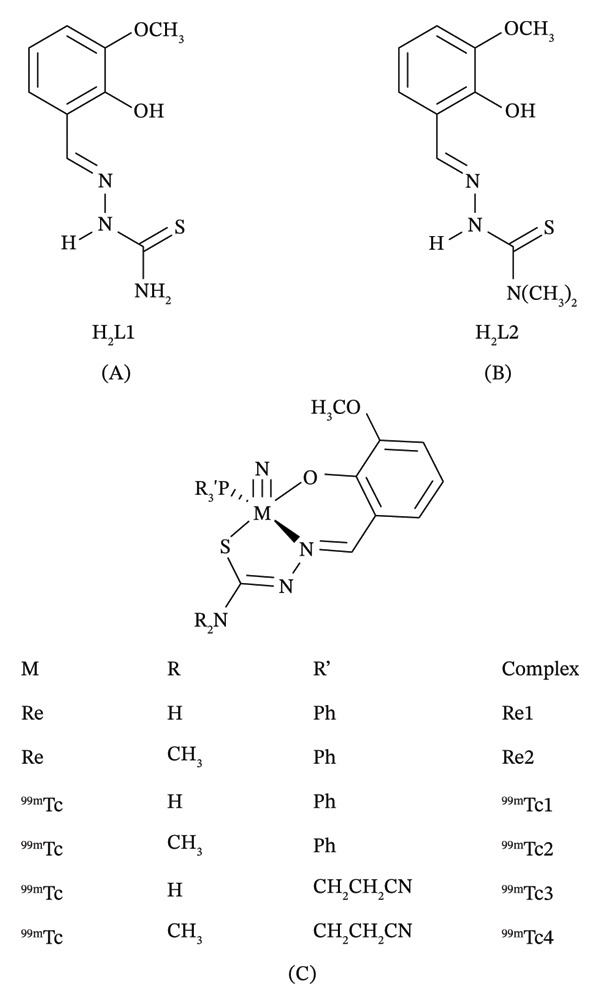
Structure of the thiosemicarbazone‐phenols proligands used in this work and the obtained rhenium and technetium‐99m complexes. (A) 3‐Methoxysalicylaldehyde thiosemicarbazone (**H**_**2**_ **L1**). (B) 3‐Methoxysalicylaldehyde N,N‐dimethyl‐thiosemicarbazone (**H**_**2**_ **L2**). (C) Complexes **Re1-2** and ^**99m**^ **Tc1-4**.

Basing on both literature reports and on our past research, we decided to explore more in depth the Tc/Re‐TSCs coordination chemistry potential, exploiting other polydentate TSCs, with the aim of developing novel platforms for the preparation of nitride‐M‐based radiopharmaceuticals. In this context, here we describe the reactivity of two tridentate thiosemicarbazone‐phenols, i.e., 3‐methoxysalicylaldehyde thiosemicarbazone, here named H_2_L1 (Figure 1A), and 3‐methoxysalicylaldehyde N,N‐dimethyl‐thiosemicarbazone, here named H_2_L2 (Figure 1B), with the M nitride core (M = rhenium, technetium‐99m), in the formation of mixed complexes along with monodentate phosphines (i.e., triphenylphosphine, PPh_3_, and tris(2‐cyanoethyl)phosphine, PCN). The general formula of the obtained complexes is reported in Figure 1C.

## 2. Materials and Methods

### 2.1. Chemicals and Reagents

All the common chemicals, triphenylphosphine (PPh_3_), tris(2‐cyanoethyl)phosphine (PCN) and ammonium perrhenate (NH_4_[ReO_4_]), were purchased from Sigma‐Aldrich/Merck (Milan, Italy). Ammonium pertechnetate‐99g NH_4_[^99g^Tc][TcO_4_] was purchased from Oak Ridge National Laboratories. Technetium‐99g is a weak β^−^ emitter (*E*_*β*_ *= *0.292 MeV; *t*_1/2_ *= *2.12 × 10_5_ years)*.* All manipulations were carried out in laboratories approved for low‐level radioactivity use. Handling milligram amounts of technetium‐99g does not present a serious health hazard because common laboratory glassware provides adequate shielding. Bremsstrahlung is not a significant problem because of the low energy of the β^−^ particles; however, proper radiation safety procedures must always be followed, and particular care should be taken when handling solid samples. Sodium pertechnetate‐99m Na[^99m^Tc][TcO_4_] was eluted from a ^99^Mo/^99m^Tc Ultra‐TechneKow generator by GE Healthcare (Milan, Italy). All the solvents were purchased from VWR International/Avantor (Milan, Italy). Aqueous solutions were prepared with Milli‐Q water (ionic purity: 18.2 MΩ ∙ cm), obtained using a Millipore system (Bedford, MA, USA). All the commercial chemicals and solvents were used without further purification. Deuterated solvents from the ampoule were used. Phosphate‐buffered saline (PBS) was obtained using the tablet formulation purchased from Sigma‐Aldrich/Merck (Milan, Italy). Rhenium nitride precursor complexes (dichloronitridobis(triphenylphosphine)nitridorhenium(V), [Re^V^NCl_2_(PPh_3_)_2_], and tetra‐n‐butylammonium tetrachloronitridorhenium(VI), (Bu_4_N)[ReN^VI^Cl_4_]) were synthesised following previously described procedures [40, 41].

Thiosemicarbazone proligands H_2_L1 and H_2_L2 were synthesised following previously described procedures [42, 43]. Solid‐phase extraction (SPE) Sep‐Pak RP‐C18 cartridges were purchased from Waters Corp. (Milford, MA, USA).

### 2.2. Instrumental Analyses

#### 2.2.1. Elemental Analysis

Carbon, hydrogen and nitrogen analyses were performed using a Vario MicroCube with autosampler (Elementar Analysensysteme GmbH).

#### 2.2.2. Mass Spectrometry

Electrospray ionization mass spectrometry (ESI–MS) analyses of rhenium nitride complexes were performed on two alternative instruments. Spectrometer A: ESI‐IT mass spectrometer, LCQ Fleet ion trap instrument (Thermo Fisher, San Jose, CA, USA), equipped with a heated‐electrospray ionisation (HESI) interface, operating in positive ion mode. The HESI parameters were as follows: source temperature: 35°C, source voltage: 4.0 kV and capillary temperature: 300°C. The ion trap mass spectrometer was operated in positive ion mode scanning in the range 50–1500 m/z. Helium was used as a buffer gas at a pressure of 1.1 × 10^−5^ Torr. Tandem mass spectrometric (MS^n^) experiments were performed by resonant excitation of the ion of interest through a supplementary r.f. voltage in the range 15%–20% of its maximum value (5 V peak‐to‐peak). Instrument control, data acquisition and processing were achieved with Qual Browser in Thermo Xcalibur 4.2.28.14. Spectrometer B: LCQ‐Duo Finnigan instrument (capillary voltage 4.5 kV; capillary temperature 200°C, helium buffer gas pressure of 1.1 × 10^−5^ Torr). Instrument control, data acquisition and processing were achieved with Qual Browser in Thermo Xcalibur 4.2.28.14. With both instruments, the compounds were dissolved in acetonitrile to obtain 10^−5^ M ca. solutions; the sample solutions were then analysed by direct infusion via a syringe pump at a flow rate of 8 µL/min.

#### 2.2.3. Infrared Spectroscopy

Fourier transform infrared (FT‐IR) spectra were recorded in the range 4000–400 cm^−1^ on a FT‐IR Tensor27 (Bruker) with OPUS software; peaks were described as s (intense), m (medium), w (weak) and br (broad).

#### 2.2.4. NMR Spectroscopy

^1^H, ^31^P{^1^H}, ^13^C{^1^H} and two‐dimensional NMR spectra were acquired in the indicated deuterated solvents at 298 K with a Bruker Avance 300 MHz (300.1 MHz, 121.5 MHz and 75 MHz for ^1^H, ^31^P and ^13^C, respectively) spectrometer with TopSpin 3.2 software. Chemical shifts are reported in ppm and referenced to internal residual solvent signal for ^1^H (CD_2_Cl_2_: 5.32 ppm and CD_3_CN: 1.93 ppm), and to deuterated solvent signal for ^13^C (CD_2_Cl_2_: 54.00 ppm and CD_3_CN: 1.3 and 117.9 ppm). Signal assignments were confirmed by 2D experiments (^1^H‐^13^C HSQC/HMQC, ^1^H‐^13^C HMBC, ^1^H‐^31^P HMBC) where necessary. Common abbreviations for signal multiplicity were used (s = singlet, d = doublets, t = triplets, q = quartets, etc.; dd = doublets of doublets; ddd = doublets of doublets of doublets; vt = virtual triplet; bs = broad singlet).

#### 2.2.5. Chromatography

Thin‐layer chromatography (TLC) analyses were performed on SiO_2_ 60 F_254_ plates or SiO_2_ 60 RP‐18 F_254S_ (Merck, Milan, Italy), using various mobile phases: Eluent A, dichloromethane/ethyl acetate/methanol (90:5:5); Eluent B, dichloromethane/ethyl acetate (95:5); Eluent C, acetonitrile with trifluoroacetic acid 0.1%. Radioactivity on TLC plates was detected and measured using a Cyclone Instrument equipped with phosphorus imaging screen and OptiQuant image analysis software (Packard, Meridian, CT). Radio/UV reverse phase high‐performance liquid chromatography (radio/UV RP‐HPLC) analyses were performed on a Dionex Ultimate 3000 instrument (Thermo Scientific, Rodano, Milan, Italy) equipped with a UV/Vis detector set at 310 nm and an additional radiometric detector Gabi Raytest, using a Waters Symmetry300TM C18 Guard Column (5.0 µm, 100 Å, 4.6 × 20 mm) and Waters Symmetry300TM RP18 Column (5.0 µm, 300 Å, 4.6 × 250 mm); Solvent A was H_2_O with trifluoroacetic acid 0.1%, Solvent B was acetonitrile with trifluoroacetic acid 0.1%; solvent flow was 1 mL/min; gradients used were as follows: (A) 0 min, %*B* = 50; 3 min, %*B* = 50; 30 min, %*B* = 70; 33 min, %*B* = 90; 39 min, %*B* = 90; 40 min, %*B* = 50; (B) 0 min, %*B* = 20; 3 min, %*B* = 20; 30 min, %*B* = 70; 33 min, %*B* = 90; 39 min, %*B* = 90; 40 min, %*B* = 20. Data were registered and elaborated by using Chromeleon 6.8 software. Radiochemical yield (RCY) values for no‐carrier‐added radiolabelled compounds were obtained by the integration of the relevant peaks as % relative area; retention times (Rt) and RCY values are given as mean ± s.d. with *n* ≥ 3 at least.

#### 2.2.6. Liquid Chromatography–Mass Spectrometry (LC–MS)

LC–MS analyses of the carrier‐added ^99g/99m^Tc complexes were performed on a HPLC Finnigan Surveyor Plus (Thermo Fisher, San Jose, CA, USA) equipped with a PDA detector and interfaced with the Spectrometer A. Instrument control, data acquisition and processing were achieved with Qual Browser in Thermo Xcalibur 4.2.28.14. Precolumn/column and gradients used were the same as indicated above for radio/UV‐RP‐HPLC analyses.

### 2.3. Synthesis of Rhenium Complexes

All syntheses were performed under N_2_ atmosphere, and the solvents and solutions were degassed and N_2_‐saturated prior to use.

#### 2.3.1. [ReNCl_2_(PCN)_2_] ∙ 4H_2_O (Re0)

In a two‐neck round‐bottom flask, a solution of PCN (92.0 mg, 0.475 mmol, 2.7 eq.) in acetone (3 mL) was brought to the boil under stirring, then a solution of (Bu_4_N)[ReNCl_4_] (103 mg, 0.175 mmol, 1 eq.) in dichloromethane (33 mL) was added in counterflow of N_2_. The resulting yellow mixture was refluxed for 75 min, during which time it turned dark orange/brown. After reflux, the reaction was quenched by dipping the flask in water‐ice. The solvent was gently removed with a N_2_ stream. The residue was dissolved in acetonitrile (6 mL), and the solution was filtered through a sintered‐glass filter (porosity 4). The product was crystallised from the filtrate by adding methanol (35 mL) and collecting the crystals by filtration. Crystallisation was repeated twice, and the crystalline powder product obtained was washed with methanol (40 mL) and diethyl ether (10 mL) and dried at reduced pressure. Yield: 50%. **Re0** appears as a natural sienna crystalline powder; it is soluble in acetonitrile, acetone, hot dichloromethane, and insoluble in methanol, ethanol, diethyl ether and hydrocarbons. Instability was observed in DMSO, with the formation of OPPh_3_. Elemental Analysis: Found: C, 30.01; H, 4.41; N, 13.32%. Calc. for C_18_H_24_N_7_P_2_Cl_2_Re ∙ 4H_2_O (MW: 675.50 Da): C, 29.63; H, 4.42; N 13.44%. ESI(þ)‐MS (on Spectrometer A): m/z 675.20 ([M + H_2_O + H]^+^ and [M + H_2_O]^+∙^, overlapped, 20%), 658.25 ([M + H]^+^ and [M]^+∙^, overlapped, 31%), 640.26 ([M + H_2_O – Cl]^+^, 61%), 622.24 ([M – Cl]^+^, 100%). M = C_18_H_24_N_7_P_2_Cl_2_Re. FT‐IR (KBr): ṽ_max_/cm^−1^ 2966 (m, ν_C-H_), 2928 (m, ν_C-H_), 2249 (m, ν_C ≡ N_), 1636 (m, br), 1420 (s, ν_C-P_), 1385 (w), 1057 (m, ν_Re-P_), 1011 (w, ν_Re ≡ N_), 953 (m), 795 (m), 741 (m). ^31^P{^1^H} NMR: δ_P_ (121.5 MHz, CD_3_CN) −2.88 (s, *P*(CH_2_CH_2_CN)_3_). ^1^H *NMR*: δ_H_ (300.1 MHz, CD_3_CN) 2.91 (m, 12H, CH_2_CN); 2.59 (m, 12H, CH_2_P), 2.15 (s, 8H, 4H_2_O, *hydration water*). ^13^C{^1^H} NMR: δ_C_ (75.5 MHz, CD_3_CN) 120.4 (*C*N); 20.2 (d, I 1PC = 14.4 Hz, P*C*H_2_); 12.9 (*C*H_2_CN).

#### 2.3.2. [ReN(L1)PPh_3_] (Re1)

##### 2.3.2.1. Procedure A

To a stirred suspension of [Re^V^NCl_2_(PPh_3_)_2_] (20.0 mg, 0.0251 mmol, 1 eq.) in dichloromethane (5 mL), a solution of H_2_L1 (14.1 mg, 0.0627 mmol, 2.5 eq.) in methanol/triethylamine (2/0.9 mL) was added in counterflow of N_2_. The obtained suspension was heated at reflux for 30 min under stirring. After 2 min of reflux, the suspension cleared, and its colour turned from bright yellow to orange. The reaction was then quenched by placing the flask in an ice‐water bath. At the end of the reaction, TLC analyses (SiO_2_ 60 F_254_ plates eluted with Solvent A and SiO_2_ 60 RP‐18 F_254S_ eluted with Solvent C) revealed the formation of the desired compound (vide infra), with apparently no by‐products.

The mixture was flushed with N_2_ to dryness, the residue was completely dissolved in dichloromethane (3 mL) and washed with aqueous sodium hydroxide (1 M, 4 × 5 mL) and water (4 × 5 mL); the extracted organic phase was dried with anhydrous sodium sulphate and then concentrated to 1 mL under a N_2_ current. The product was then crystallised by adding n‐hexane (5 mL); crystals were then collected by filtration and washed with *n*‐hexane (2 mL). Yield = 88%.

##### 2.3.2.2. Procedure B

To a stirred suspension of [Re^V^NCl_2_(PPh_3_)_2_] (20.0 mg, 0.0251 mmol, 1 eq.) in methanol (4 mL), a suspension of H_2_L1 (7.4 mg, 0.0327 mmol, 1.3 eq.) in methanol/dichloromethane (2:1, 3 mL) was added in counterflow of N_2_. The obtained mixture was heated at reflux for 2.5 h with stirring. After 5 min of reflux, the suspension cleared, and its colour turned from pale green to orange‐red, gradually darkening during the reaction. At the end of the reaction, TLC analyses (SiO_2_ 60 F_254_ plates eluted with eluent A and SiO_2_ 60 RP‐18 F_254S_ eluted with Eluent C) revealed the formation of the desired compound (vide infra) together with some by‐products. The mixture was dried in current of N_2_, and the residue was dissolved in dichloromethane (3 mL) and filtered on a sintered‐glass filter of porosity 4 to remove the insoluble products. The filtrate was washed with aqueous sodium hydroxide (1 M, 4 × 5 mL) and with water (4 × 5 mL); the extracted organic phase was dried with anhydrous sodium sulphate and then concentrated to 1 mL under an N_2_ current. The product was then crystallised by adding *n*‐hexane (5 mL); crystals were then collected by filtration and washed with *n*‐hexane (4 × 2 mL). Yield = 18%.

##### 2.3.2.3. Procedure C

To a stirred and boiling solution of (Bu_4_N)[Re^VI^NCl_4_] (13.0 mg, 0.0222 mmol, 1 eq.) and PPh_3_ (29.2 mg, 0.111 mmol, 5 eq.) in acetone (5 mL), a solution of H_2_L1 (12.5 mg, 0.0555 mmol, 2.5 eq.) in acetone/triethylamine (4/0.9 mL) was added in counterflow of N_2_. The solution was heated at reflux for 30 min under stirring. During this time, the solution colour turned from yellow to orange, and then, it became progressively darker. The reaction was then quenched by placing the flask in an ice‐water bath. At the end of the reaction, TLC analyses (SiO_2_ 60 F_254_ plates eluted with Solvent A and SiO_2_ 60 RP‐18 F_254S_ eluted with Solvent C) revealed the formation of the desired compound (vide infra) with some by‐products.

The reaction mixture was then treated as indicated in Procedure A. Yield: 29%.

**Re1** appears as an orange powder (or a red‐crystalline solid if aged in a crystallisation mixture of dichloromethane/*n*‐hexane); it is soluble in dichloromethane, methanol, acetone, acetonitrile, dimethyl sulfoxide, insoluble in water, diethyl ether and hydrocarbons. It appears to be instable when exposed to prolonged contact with silica gel, becoming darker/brownish and degrading in several compounds. That is why we avoided chromatographic purifications. Suitable red, prismatic crystals for XRD analysis were obtained by slow diffusion of diethyl ether into a dichloromethane solution, at 4°C in 5 days.

Elemental Analysis: Found: C, 47.11; H, 3.37; N, 7.91%. Calc. for C_27_H_24_N_4_O_2_PSRe (MW: 685.75 Da): C, 47.29; H, 3.53; N, 8.17%. ESI(+)‐MS (Spectrometer B): m/z 708.86 ([M + Na]^+^ 100%), 1394.54 ([2M + Na]^+^, 30%). M = C_27_H_24_N_4_O_2_PSRe. FT‐IR (KBr): ṽṽ_max_/cm^−1^ 3306 (w, ν_N-H_), 3175 (w, ν_N-H_), 2962 (m, ν_C-H_), 2935 (w, ν_C-H_), 2874 (w, ν_C-H_), 1720 (m, δs_NH2_), 1600 (s), 1555 (m), 1528 (s), 1434 (s, δ_CH2_), 1281 (m), 1254 (s), 1091 (s, ν_C-P_), 1076 (m, ν_Re ≡ N_), 968 (w), 910 (s), 744 (m, ν_C=C_), 694 (m, ν_C=C_), 532 (m), 509 (w).^31^P{^1^H} NMR: δ_P_ (121.5 MHz, CD_2_Cl_2_) 29.97 (s, *P*Ph_3_). ^1^H NMR: δ_H_ (300.1 MHz, CD_2_Cl_2_) 8.80 (d, I 4PH = 2.88 Hz, 1H, ReNC*H*); 7.79 (ddd, I 3PH = 11.63 Hz, I 3HH = 7.61 Hz, I 5HH = 1.78 Hz, 6H, *H*_*o*_ PPh_3_); 7.55–7.39 (m, 9H, *H*_*m*_ + *H*_*p*_ PPh_3_); 7.06 (dd, I 3HH = 7.91 Hz, I 5HH = 1.52 Hz, 1H, C_arom_ *H*(COMe) L1^2−^); 7.02 (dd, I 3HH = 7.88 Hz, I 5HH = 1.52 Hz, 1H, C_arom_ *H*(C_arom_H) L1^2−^); 6.83 (vt, I 3HH = 7.80 Hz, 1H, C_arom_ *H*(C_arom_H)_2_ L1^2−^); 5.44 (s, 2H, NH_2_); 3.63 (s, 3H, OCH_3_). ^13^C{^1^H} NMR: δ_C_ (75.5 MHz, CD_2_Cl_2_) 183.6 (*C*SRe); 159.5 (C_arom_ORe, N*C*H); 152.1 (C_arom_OMe); 135.0 (d, I 2CP = 11.48 Hz, C_orto_ PPh_3_); 133.3 (d, I 1CP = 53.28 Hz, C_ipso_ PPh_3_); 131.3 (d, I 4CP = 2.43 Hz, C_para_ PPh_3_); 129.1 (d, I 3CP = 10.59 Hz, C_meta_ PPh_3_); 126.3 (C_arom_ *H*(COMe) L1^2−^); 120.1 (C_arom_(C)_3_ L1^2−^); 118.4 (C_arom_H(C_arom_H)_2_ L1^2−^); 116.6 ((C_arom_)C_arom_H(C_arom_H) L1^2−^); 56,6 (OCH_3_). UV/vis‐RP‐HPLC (Method A, λ = 310 nm): *R*_*t*_= 12.48 min. TLC: SiO_2_, Eluent A, *R*_*f*_ = 0.91 (pale orange spot); SiO_2_ RP‐18, Eluent C, *R*_*f*_ = 0.70 (pale orange spot).

#### 2.3.3. [ReN(L2)PPh_3_] (Re2)

##### 2.3.3.1. Procedure A

To a stirred and boiling suspension of [Re^V^NCl_2_(PPh_3_)_2_] (20.0 mg, 0.0252 mmol, 1 eq.) in dichloromethane (5 mL), a yellow solution of H_2_L2 (15.82 mg, 0.0627 mmol, 2.5 eq.) in methanol/triethylamine (2/0.9 mL) was added in counterflow of N_2_. The mixture immediately cleared, and its colour turned from bright yellow to orange‐red. The reflux was maintained for 35 min, and then, the reaction was quenched by placing the flask in an ice‐water bath. TLC analyses (SiO_2_ 60 F_254_ plates eluted with solvent B and SiO_2_ 60 RP‐18 F_254S_ eluted with Solvent C) revealed formation of the desired compound (vide infra), together with some by‐products.

The mixture was brought to dryness in a current of N_2_, the residue was dissolved in dichloromethane (10 mL), and this solution was washed with aqueous sodium hydroxide (1 M, 4 × 5 mL) and with water (4 × 5 mL); the extracted organic phase was dried with anhydrous sodium sulphate and then concentrated to 5 mL under a N_2_ current. The solution was then loaded into a gravity chromatographic column (Ø 2 cm, height 12 cm) filled with SiO_2_ (grade 9385, porosity 60 Å), conditioned with dichloromethane and eluted with dichloromethane (43 mL) followed by dichloromethane/ethyl acetate 95/5 (40 mL), then dichloromethane/ethyl acetate 17/1 (60 mL). The isolated product, eluted from 56 to 120 mL, was dried in a current of N_2_ and then under vacuum. Yield: 63%.

##### 2.3.3.2. Procedure B

To a stirred suspension of [Re^V^NCl_2_(PPh_3_)_2_] (20.0 mg, 0.0251 mmol, 1 eq.) in methanol (3 mL), a solution of H_2_L2 (8.3 mg, 0.0330 mmol, 1.3 eq.) in methanol (3 mL) was added in counterflow of N_2_. The obtained suspension was heated at reflux for 7 h under stirring. After 5 min of reflux, the suspension cleared, and its colour changed from pale green to orange‐red and then became progressively darker. At the end of the reaction, TLC analyses (SiO_2_ 60 F_254_ plates eluted with Solvent B and SiO_2_ 60 RP‐18 F_254S_ eluted with Solvent C) revealed formation of the desired compound (vide infra), together with some by‐products.

The mixture was dried in a current of N_2_, and the residue was completely dissolved in dichloromethane (3 mL) and washed with aqueous sodium hydroxide (1 M, 4 × 5 mL) and water (4 × 5 mL); the extracted organic phase was dried with anhydrous sodium sulphate and then concentrated to 1 mL under a N_2_ current. The solution was then loaded into a gravity chromatographic column (Ø 1 cm, height 20 cm) filled with SiO_2_ (grade 9385, porosity 60 Å), conditioned with dichloromethane/ethyl acetate (95:5) and eluted with the same solvent. The isolated product, eluted after 10 mL of eluent, was dried in a current of N_2_ and then under vacuum. Yield: 23%.

**Re2** appears as an orange powder; it is soluble in dichloromethane, methanol, acetone, acetonitrile, dimethyl sulfoxide and insoluble in water, diethyl ether and hydrocarbons. Suitable orange, tabular crystals for XRD analysis were obtained by slow diffusion of *n*‐hexane into a dichloromethane solution, at 4°C in 2 days.

Elemental Analysis: Found: C, 48.77; H, 3.85; N, 7.56%. Calc. for C_29_H_28_N_4_O_2_PSRe (MW: 713.80 Da): C, 48.80; H, 3.95; N, 7.85%. ESI(+)‐MS (Spectrometer B): m/z 713.97 (M^+∙^, 100%), 1163.88 ([2M–PPh_3_]^+∙^, 20%), 1490.88 ([2M + MeCN + Na]^+^, 20%); M = C_29_H_28_N_4_O_2_PSRe. FT‐IR (KBr): *ṽ*_max_/cm^−1^ 3055 (w, ν_C-H_), 3004 (w, ν_C-H_), 2924 (m, br, ν_C-H_), 1593 (s, ν_C=N_), 1555 (m), 1528 (s), 1435 (s, δ_CH2_), 1385 (m), 1354 (w), 1285 (m), 1250 (s), 1138 (m), 1091 (m, ν_P-C_), 1076 (s, ν_Re ≡ N_), 980 (w), 906 (m), 860 (w), 744 (m, ν_C=C_), 694 (s, ν_C=C_), 571 (w), 532 (s), 509 (m). ^31^P{^1^H} NMR: δ_P_ (121.5 MHz, CD_2_Cl_2_) 31.24 (s, *P*Ph_3_). ^1^H NMR: δ_H_ (300.1 MHz, CD_2_Cl_2_) 8.84 (d, I 4PH = 2.73 Hz, 1H, NC*H*); 7.80 (ddd, I 3PH = 11.56 Hz, I 3HH = 7.55 Hz, I 5HH = 1.72 Hz, 6H, C_orto_H PPh_3_); 7.54–7.39 (m, 9H, C_meta_ *H* + C_para_ *H* PPh_3_); 7.05 (dd, I 3HH = 7.79 Hz, I 5HH = 1.48 Hz, 1H, C_arom_ *H*(C_ar_ *H*) L2^2-^); 6.98 (dd, I 3HH = 7.62 Hz, I 5HH = 1.48 Hz, 1H, C_arom_ *H*(COMe) L2^2−^); 6.82 (vt, I 3HH = 7.81 Hz, 1H, C_arom_ *H*(C_arom_ *H*)_2_ L2^2−^); 3.59 (s, 3H, OCH_3_); 3.36 (s, 6H, N(CH3)_2_). ^13^C{^1^H} NMR: δ_C_ (75.5 MHz, CD_2_Cl_2_) 185.2 (d, I 3CP = 3.71 Hz, *C*SRe); 158.2 (C_arom_ORe); 156.6 (N*C*H); 151.4 (C_arom_OMe); 134.5 (d, I 2CP = 11.28 Hz, C_orto_ PPh_3_); 132.9 (d, I 1CP = 52.68 Hz, C_ipso_ PPh_3_); 130.6 (d, I 5CP = 2.29 Hz, C_para_ PPh_3_); 128.5 (d, I 3CP = 10.68 Hz, C_meta_ PPh_3_); 125.4 ((C_arom_)C_arom_H(C_arom_H) L2^2−^); 120.1 (C_arom_(C)_3_ L2^2−^); 117.7 (C_arom_H(C_arom_H)_2_ L2^2−^); 115.5 (C_arom_H(COMe) L2^2−^); 56.0 (OCH_3_); 41.3 (NCH_3_). UV/vis‐RP‐HPLC (method A, λ = 310 nm): *R*_*t*_ = 20.84 min. TLC: SiO_2_, Eluent B, *R*_*f*_ = 0.89 (pale orange spot); SiO_2_ RP‐18, Eluent C, *R*_*f*_ = 0.62 (pale orange spot).

### 2.4. XRD Analysis

The measurements were collected at room temperature on a SuperNova diffractometer (Rigaku/Oxford Diffraction) equipped with a microfocus source Mo Kα radiation (λ = 0.71073 Å, diameter of beam section 120 µm, electric potential 50 kV and current intensity 0.12 mA), and with an area detector Pilatus 200K (Dectris). The diffraction intensities were corrected for Lorentz and polarisation effects and for absorption. Empirical multiscan absorption corrections using equivalent reflections were performed with the scaling algorithm SCALE3 ABSPACK. Data collection, reduction and finalisation were done with the CrysAlisPro CrysAlisPro1.171.42.49 software (Rigaku Oxford Diffraction, 2022). Accurate unit cell parameters were determined by least‐squares refinement of the 18,255 (H_2_L2), 38,984 (**Re1**), 4223 (**Re2**) strongest reflections chosen from the whole experiment. The structures were solved by means of the intrinsic phasing method using SHELXT [44] (H_2_L2 and **Re1**) and the Patterson method using SHELXS [45] (**Re2**) and refined on |Fo|^2^ with SHELXL [46]. The solution and refinement programmes were used through the interface Olex2 [47]. See Supporting Information for detailed crystal data (Table S1).

### 2.5. Synthesis of Technetium‐99m Complexes: General Procedures

All the solvents and solutions were degassed and N_2_‐saturated before use.

#### 2.5.1. Preliminary Operations

The following solutions were freshly prepared. *Solution A*: 0.0052 mmol of the relevant phosphine in 0.775 mL of a solvent prepared by mixing 3.375 mL of ethanol and 0.500 mL of HCl 1 M; *Solution B*: 0.0040 mmol of the relevant thiosemicarbazone ligand in 0.075 mL of a solvent obtained by mixing 1.00 mL of ethanol and 0.040 mL of aqueous NaOH 1 M.

##### 2.5.1.1. Procedure A

 
*Step 1.* To a capped and N_2_‐saturated vial containing succinic dihydrazide (5.0 mg) was added a 1 mg/mL suspension of tin(II) chloride in physiologic saline solution (0.1 mL), followed by freshly eluted Na[^99m^Tc][TcO_4_] saline solution (0.500 mL, 50−1500 MBq). The mixture was kept at room temperature for 15 min.
 
*Step 2.* To the mixture obtained in *Step 1* were added, in sequence: 0.500 mL of aqueous NaH_2_PO_4_ 0.2 M, pH 5, *Solution A* and *Solution B.* Then, the pH of the resulting mixture was adjusted to 5 by dropping aqueous NaOH 1 M. The final mixture was heated at 100°C for 30 min.

##### 2.5.1.2. Procedure B

 
*Step 1.* To a capped and N_2_‐saturated vial containing succinic dihydrazide (5.0 mg), were added, in sequence, *Solution A* and a freshly eluted Na[^99m^Tc][TcO_4_] saline solution (0.500 mL, 50−1500 MBq). The mixture was heated at 100°C for 15 min.
 
*Step 2.* To the mixture obtained in *Step 1* were added, in sequence, 0.500 mL of aqueous NaH_2_PO_4_ 0.2 M pH 5 and *Solution B.* Then, the pH of the labelling mixture was adjusted to 5 by dropping aqueous NaOH 1 M. The final mixture was heated at 100°C for 30 min.

 [^99m^Tc][TcN(L1)PPh_3_] (^**99m**^ **Tc1**). RCY = 95.98 ± 2.75%. HPLC R_t_ (Gradient A): 13.07 ± 0.23 min.
 [^99m^Tc][TcN(L2)PPh_3_] (^**99m**^ **Tc2**). RCY = 87.67 ± 1.82%. HPLC R_t_ (Gradient A): 22.01 ± 0.61 min.
 [^99m^Tc][TcN(L1)PCN] (^**99m**^ **Tc3**). RCY = 98.15 ± 1.06%. HPLC R_t_ (Gradient B): 13.50 ± 0.38 min.
 [^99m^Tc][TcN(L2)PCN] (^**99m**^ **Tc4**). RCY = 92.70 ± 1.31%. HPLC R_t_ (Gradient B): 18.33 ± 0.10 min.

#### 2.5.2. Purification of Technetium‐99m Nitride Complexes

A reversed‐phase C18 SPE cartridge (Sep‐Pak, Waters) was preconditioned with ethanol (5.0 mL) followed by HPLC grade water (5.0 mL).

The relevant labelling mixture was diluted with water (20 mL) and loaded on the cartridge. Almost all the activity present in the mixture was retained on the cartridge. The loaded cartridge was then washed with HPLC grade water (20 mL), ethanol 20% v/v in HPLC grade water (10 mL) and ethanol 30% v/v in HPLC grade water (5 mL). Final elution of complexes was performed using 2 mL of a mixture of ethanol/physiologic saline solution 90/10 (for ^**99m**^ **Tc1** and ^**99m**^ **Tc2**) or 70/30 (for ^**99m**^ **Tc3** and ^**99m**^ **Tc4**). 85%–90% of the loaded activity was collected. The radiochemical purity (RCP) of the complexes after SPE purification was almost 100%.

**FIGURE 2:**
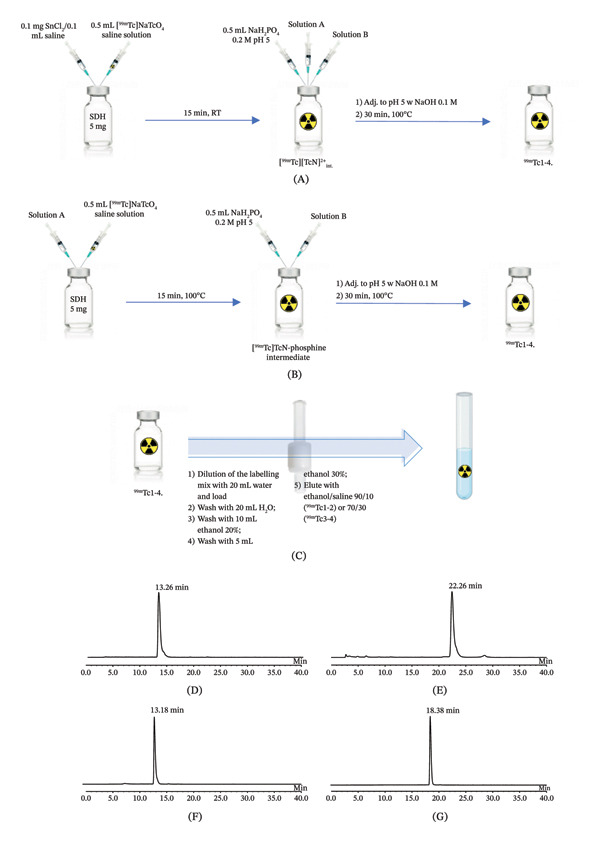
Synthesis and purification of _**99m**_ **Tc1-4**. (A) Scheme of Procedure A. (B) Scheme of Procedure B. (C) Scheme of the SPE purification on C18 cartridge. (D–G) radio‐RP‐HPLC chromatograms of _**99m**_ **Tc1-4** after purification. For specifications about solution A, solution B, and RP‐HPLC column and gradients, see main text.

A scheme of the whole procedures of synthesis and purification of ^**99m**^ **Tc1-4** is depicted in Figure 2.

### 2.6. Synthesis of Carrier‐Added Technetium‐99g/99m Complexes: General Procedure

All the solvents and solutions were degassed and N_2_‐saturated before use.

#### 2.6.1. Preliminary Operations

The following solutions were freshly prepared. Solution A: 0.010 mmol of the relevant phosphine in 0.775 mL of a solvent prepared by mixing 3.375 mL of ethanol and 0.500 mL of HCl 1 M; Solution B: 0.0079 mmol of the relevant thiosemicarbazone ligand in 0.075 mL of a solvent obtained by mixing 1.00 mL of ethanol and 0.040 mL of aqueous NaOH 1 M.

 
*Step 1.* To a capped and N_2_‐saturated vial containing succinic dihydrazide (15.0 mg) and tin(II) chloride (2.0 mg) were added, in sequence: physiologic saline solution (0.1 mL), freshly eluted Na[^99m^Tc][TcO_4_] saline solution (0.350 mL, 50−1500 MBq) and a 1 mg/mL solution of (NH_4_)[^99g^Tc][TcO_4_] in physiologic saline (0.150 mL). The mixture was kept at room temperature for 30 min; during this time, it turned to pale yellow.
 
*Step 2.* To the mixture obtained in Step 1 were added, in sequence: 0.500 mL of aqueous NaH_2_PO_4_ 0.2 M pH 5, Solution A and Solution B. Then, the pH of the labelling mixture was adjusted to 5 by dropping aqueous NaOH 1 M. The final mixture was heated at 100°C for 30 min.

The reaction mixtures were first analysed by radio/UV RP‐HPLC to ascertain the formation of the desired products; then, they were purified as described above, mainly to remove excess SDH, tin chloride and buffers. Purified products were analysed by radio/UV‐RP‐HPLC and LC–MS.

### 2.7. TLC and HPLC Comparison of Re1/^99m^Tc1 and Re2/^99m^Tc2

TLC comparison of the obtained rhenium complexes, **Re1** and **Re2**, and the corresponding technetium‐99m radiocomplexes, ^**99m**^ **Tc1** and ^**99m**^ **Tc2**, were performed by eluting with the appropriate eluent (A, B or C) on the same TLC plate (SiO_2_ 60 F_254_ or SiO_2_ 60 RP‐18 F_254S_), each pure rhenium compound and the labelling mixture of the corresponding technetium‐99m complex.

UV/radio‐HPLC comparison was performed by mixing 0.5 mL of a methanolic solution (5 mM) of the relevant pure rhenium complex with 0.02 mL of the labelling mixture of the corresponding technetium‐99m complex, injecting 0.01 mL of the resulting mixture into the HPLC instrument and running the proper elution program.

Chromatographic data and yields for all the rhenium and technetium‐99m complexes are summarised in Table 1.

**TABLE 1:** Chromatographic data and yields for the complexes Re1‐2 and^99m^Tc1‐4.

	TLC (*R*_*f*_)	HPLC (*R*_*t*_, min)	Yield (%)	RCY (%)
**Re1**	0.91^1^	12.48 (12.39)^4^ ^,^ ^6^	88^7^	—
	0.70^2^			
**^99m^Tc1**	0.94^1^	13.07 ± 0.23^4^	—	95.98 ± 2.75
	0.66^2^			
**Re2**	0.89^3^	20.84 (20.76)^4^ ^,^ ^6^	63^7^	—
	0.62^2^			
**^99m^Tc2**	0.90^3^	22.01 ± 0.61^4^	—	87.67 ± 1.82
	0.58^2^			
**^99m^Tc3**	—	13.50 ± 0.38^5^	—	98.15 ± 1.06
**^99m^Tc4**	—	18.33 ± 0.10^5^	—	92.70 ± 1.31

*Note:* For values expressed as mean ± s.d., *n* ≥ 3 at least.

^1^SiO_2_, Eluent A.

^2^SiO_2_ RP‐18, Eluent C.

^3^SiO_2_, Eluent B.

^4^Gradient A.

^5^Gradient B.

^6^Data obtained from a single run, in brackets, the value of the corresponding technetium‐99m complex obtained by co‐elution as detailed in the Materials and Methods.

^7^Procedure B.

### 2.8. Stability Studies on Technetium‐99m Nitride Complexes

All the assays described in this paragraph were performed in triplicate by using the radiocomplexes purified as described above.

#### 2.8.1. Stability in PBS

An aliquot (0.100 mL) of the relevant technetium‐99m nitride complex solution was added to a propylene test tube containing PBS pH 7.4 (0.900 mL). The mixture was vortexed and then incubated at 37°C for 24 h. At 0.5, 1, 3 and 24 h, aliquots of the reaction mixture were withdrawn and analysed by radio‐HPLC, using the appropriate elution gradient, evaluating the RCP of the compound as the per cent relative area of the relevant peak.

#### 2.8.2. Cysteine and Glutathione Challenge

An aliquot (0.100 mL) of an aqueous stock solution of cysteine hydrochloride (10 mM) was added to a propylene test tube containing PBS (0.800 mL, pH 7.4) and the relevant technetium‐99m nitride complex solution (0.100 mL). The mixture was vortexed and then incubated at 37°C for 24 h. A control batch containing an equal volume of water instead of cysteine hydrochloride was studied in parallel. At 0.5, 1, 3 and 24 h, aliquots of the reaction mixture were withdrawn and analysed by radio‐HPLC using the appropriate elution method, assessing the RCP of the compound as the per cent relative area of the relevant peak. A similar procedure was applied using a glutathione stock solution (0.100 mL, 10 mM) as challenger.

#### 2.8.3. Stability in Human Serum

In a propylene test tube, 0.100 mL of the relevant technetium‐99m nitride complex solution was added to 0.900 mL of human serum. The resulting mixture was vortexed and then incubated at 37°C for 24 h. At 0, 40, 80, 120, 160, 200 min and 24 h, 0.200 mL of the mixture was withdrawn, mixed with 0.200 mL of cold acetonitrile with 0.1% trifluoroacetic acid and ultracentrifuged (3000 rpm for 10 min); then, 0.100 mL of the supernatant was conveniently diluted with water and analysed by radio‐HPLC using the appropriate elution method, assessing the RCP of the compound as the per cent relative area of the relevant peak.

#### 2.8.4. Binding to Plasma Proteins

The same mixture prepared for the human serum stability (vide supra), incubated at 37°C, was also used for this assessment. At 15, 40, 80, 180 min and 24 h, 0.025 mL of the mixture was withdrawn and loaded on a pre‐spun (735 g for 2 min) Sephadex G‐50 mini‐column. The column was centrifuged at 735 g for 1 min. The collected eluate and the column were counted in a NaI‐scintillation counter. The protein‐bound complex was calculated as (eluate activity/total activity) × 100.

## 3. Results and Discussion

### 3.1. Synthesis of Rhenium Complexes

One of the most common rhenium(V) nitride precursors is the complex [ReNCl_2_(PPh_3_)_2_], where the chlorides and monodentate phosphine ligands can be easily exchanged with other chelators [40, 48, 49]. The reaction between this precursor and salicylthiosemicarbazone H_2_L1 and H_2_L2 leads to the final compounds of general formula [ReN(L)PPh_3_] (L = L1^2−^, **Re1**; L2^2−^, **Re2**) in high to good yield. The yield depends on the reaction conditions used. A mandatory requirement is moderate heating, since no reaction occurs at room temperature. At reflux, best results are achieved with an excess of salicylthiosemicarbazone ligand (2.5 equivalents) and a large excess of triethylamine, by using a mixture of dichloromethane and methanol 2.5:1 *ca.* as solvent. Under the optimised conditions of Procedure A (see Materials and Methods), **Re1** was obtained with an 88% yield and **Re2** with 63% yield. Conversely, according to Procedure B, using a neat methanol solution or a methanol/dichloromethane mixture with a reduced dichloromethane content, and/or lacking triethylamine, and/or employing a lower amount of thiosemicarbazone ligand, reaction resulted in significantly lower yields, and the formation of several by‐products was observed. The superior performance of the 2.5:1 dichloromethane/methanol mixture is probably attributable to its lower reflux temperature (*ca.* 40°C). The higher reflux temperature of pure methanol (65°C) appears to promote degradation, leading to the observed by‐products. The role of triethylamine is evident: it assists the deprotonation of the thiosemicarbazone ligand and scavenges the chloride ions. For both procedures A and B, the formation of the complexes [ReN(L)PPh_3_] is readily detectable through a colour change of the reaction mixtures, shifting from bright yellow (Procedure A) or pale green (Procedure B) to red/orange. TLC, detailed in Experimental, was used to detect the reaction progress and completion, as well as by‐product formation. Procedure B required a longer reaction time and yielded a higher number and amount of by‐products. The work‐up procedure for **Re1** entails a simple filtration/extraction (filtration is necessary only for Procedure B), while for **Re2**, a gravity column chromatography is needed, even with the more performant Procedure A. Complex **Re1** was also synthesised starting from the rhenium(VI) precursor (Bu_4_N)[ReNCl_4_] (Materials and Methods, Procedure C). In this approach, triphenylphosphine served as both ligand and reducing agent. Due to the observed instability of (Bu_4_N)[ReNCl_4_] in methanol, the methanol/dichloromethane solvent mixture utilised in procedures A and B was replaced with acetone, a more suitable solvent for H_2_L1. The work‐up procedure was analogous to that described for procedure A, involving the extraction of the unreacted precursor and the ligand excess with alkaline water. However, this procedure yielded a lower amount of product than Procedure A.

In principle, Procedure C used to obtain **Re1** offers a way for broadening this complex class by using diverse monodentate phosphines. To explore this, tris(2‐cyanoethyl)phosphine (PCN) was chosen as a co‐ligand. PCN is an air‐stable, easy‐to‐handle alkyl‐phosphine with reduced lipophilicity. This feature might be useful for future possible applications. Nevertheless, the complex [ReN(L)PCN] was never obtained, despite several attempts performed under different reaction conditions; these efforts included the use of the newly synthesised precursor nitridodichlorobis[tris(2‐cyanoethyl)phosphine] rhenium(V), [Re^V^NCl_2_(PCN)_2_] (**Re0**), and also in this case, the desired product was not formed (see the Supporting information (available here) for details). Notably, technetium‐99m PCN‐based complexes, both with L1^2−^ and L2^2−^, were successfully obtained (*vide infra*).

### 3.2. Characterisation of Rhenium Complexes

Rhenium **Re0-2** complexes were characterised by elemental analysis, ESI–MS, FT‐IR spectroscopy (in the 4000–400 cm^−1^ region) and NMR spectroscopy.

#### 3.2.1. Elemental Analysis

Elemental analysis of **Re0** is compatible with the tetrahydrate, [ReNCl_2_(PCN)_2_] ∙ 4H_2_O; data for **Re1** and **Re2** match the proposed formula [ReNL(PPh_3_)] (L = L1^2−^ or L2^2−^).

#### 3.2.2. ESI–MS

ESI(+)–MS analysis of Re0‐2 was performed on 10^−5^ M acetonitrile solutions. The spectrum of **Re0** (Figures S1–S6) displays a base peak at m/z 622.24, which is compatible with the ion generated from the loss of a chloride ligand, [M–Cl]^+^ (M = C_18_H_25_Cl_2_N_7_P_2_Re, consistent with [ReNCl_2_(PCN)_2_]). Minor peaks at m/z 657 and 658 correspond to [M]^+∙^ and [M + H]^+^; isotopic envelopes are overlapped, and the simulation suggests that the two ions are in a 0.3:1 ratio. Hydrate‐related ions are observed at m/z 640.26 ([M–Cl + H_2_O]^+^), m/z 675 [M + H_2_O]^+∙^ and m/z 676 [M + H_2_O + H]^+^; the isotopic envelopes of [M + H_2_O]^+∙^ and [M + H_2_O + H]^+^ are overlapped, and simulation suggests a 1:1 ratio. These species likely arise from the hydration water, in agreement with the elemental analysis. Tandem MS^n^ experiments (Figures S7–S12) support the proposed ion structures and the overall formulation of **Re0**.

**Re1** (Figures S13–S14) exhibits a base peak at m/z 708.86 ([M + Na]^+^, M = C_27_H_24_N_4_O_2_PSRe, consistent with [ReN(L1)(PPh_3_)]), and a dimeric ion at m/z 1394.54 ([2M + Na]^+^).

**Re2** (Figures S15 and S16) displays the molecular ion at m/z 713.97 ([M]^+∙^, M = C_29_H_28_N_4_O_2_PSRe, consistent with [ReN(L2)(PPh_3_)]), along with signals at m/z 1163.88 ([2M–PPh_3_]^+∙^) and 1490.88 ([2M + CH_3_CN + Na]^+^).

All signal assignments are confirmed by agreement between experimental and simulated isotopic patterns.

#### 3.2.3. FT‐IR Spectroscopy

The FT‐IR spectrum of **Re0** clearly displays the diagnostic absorptions of the C≡N (2249 cm^−1^) and Re≡N (1057 cm^−1^) stretching vibrations. For **Re1** and **Re2**, comparison with the spectra of the free ligands is very informative. The hydrazinic N–H stretching band (3171 cm^−1^ in H_2_L1 and 3302 cm^−1^ in H_2_L2) is absent, confirming deprotonation upon chelation. In **Re1**, the two thioamide N–H bands are shifted to lower frequencies relative to the free ligand, as typically observed upon coordination [21]. Absorptions between 1720 and 1460 cm^−1^ arise from vibrations of groups directly involved in metal coordination (δ_N–H_, ν_C–N_), accounting for the pronounced differences between the complexes and the free ligands in this region. The Re≡N and P–C stretching modes appear at 1076 and 1091 cm^−1^, respectively. The 821–767 cm^−1^ region is less crowded in the spectra of the complexes, due to the disappearance of phenolic O–H out‐of‐plane bending and the restricted motion of the aromatic C–O bond upon chelation. The **Re1** spectrum also exhibits the δ_s_(NH_2_) band, which is absent in **Re2**. Spectra are reported in Figures S17, S19 and S21; comparisons with the free ligands are shown in Figures S18, S20 and S22.

#### 3.2.4. NMR Spectroscopy

**Re0-2** were characterised by ^31^P, ^1^H and ^13^C NMR spectroscopy at 298 K. Two‐dimensional homo‐ and heteronuclear experiments were performed when required for reliable resonance assignments. Spectra are reported as Supporting Information (Figures S23–S38).

As expected, the complexes are diamagnetic and give spectra with well‐resolved, sharp peaks in narrow windows.

**Re0** spectra were recorded in acetonitrile‐d_3_. The ^31^P NMR spectrum displays a singlet at −2.88 ppm, downfield from free PCN (−24.09 ppm, Figures S39), reflecting coordination to the rhenium nitride core. This value is unusual for phosphines bound to the same core, which typically resonate between 20 and 50 ppm [50–52]. The ^1^H spectrum shows two expected methylene multiplets and a water peak integrating for eight protons, consistent with four water molecules as suggested by elemental analysis. In the ^13^C spectrum, resonances appear at 120.4 ppm (singlet, nitrile C), 20.2 ppm (doublet, J 1PC = 14.4 Hz, CH_2_ directly bound to P) and 12.9 ppm (CH_2_ in beta position to the P atom). ^31^P‐^1^H and ^1^H‐^13^C HMBC correlations confirm these assignments.

Spectra of **Re1** and **Re2** were recorded in dichloromethane‐d_2_. Their phosphorus resonances at 29.97 and 31.24 ppm (*vs.* −5.5 ppm for free PPh_3_ in the same solvent [53]) are consistent with coordination of the phosphine to the rhenium nitride core [50–52]. The ^1^H spectra are diagnostic of coordination of both thiosemicarbazonate‐phenolate and phosphine ligands, consistent with the formulation [ReN(L) (PPh_3_)]. Integration gives 24 and 28 protons for **Re1** and **Re2,** respectively, corresponding to one triphenylphosphine and one doubly deprotonated L (L1^2−^ or L2^2−^) bound to the rhenium centre. The phenolic and hydrazinic proton signals of the free ligands (broad singlets at *ca.* 11 and 9 ppm; Figure S40 for H_2_L2 as an example) disappear upon complexation. The iminic proton shifts downfield upon coordination, whereas the methyl protons of H_2_L2 shift upfield due to increased electron density after phenolic deprotonation. Furthermore, in both **Re1** and **Re2**, the iminic signal appears as a doublet rather than a singlet. Indeed, ^31^P‐^1^H HMBC spectra reveal a clear correlation of this nucleus with the phosphorus one, evidenced by a scalar I 4 coupling mediated by metal orbitals, thus confirming the coexistence of triphenylphosphine and L^2−^ as ligands. A comparison of the ^1^H spectra of H_2_L2 and **Re2** is provided in Figure S41. The ^13^C NMR data are consistent with the proposed structures, with full assignments obtained from 2D HMQC/HSQC and HMBC experiments, although they provide less diagnostic information than the proton spectra.

### 3.3. Single‐Crystal X‐Ray Diffractometry of H_2_L2, Re1 and Re2

Green tabular crystals of the proligand H_2_L2, belonging to the monoclinic crystal system (space group *P*2_1_ */n*, Figure S42), grew in an NMR tube by slow evaporation of a CD_2_Cl_2_ solution at 4°C. The asymmetric unit contains three independent molecules of the proligand and a molecule of water (Figure S43).

**FIGURE 3:**
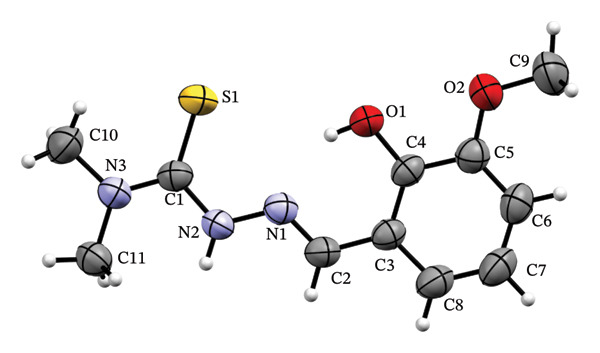
ORTEP diagram of **H**_**2**_ **L2** molecule. Ellipsoids at 50% of probability, H atoms drawn as fixed‐size spheres with a diameter of 0.15 Å.

**FIGURE 4:**
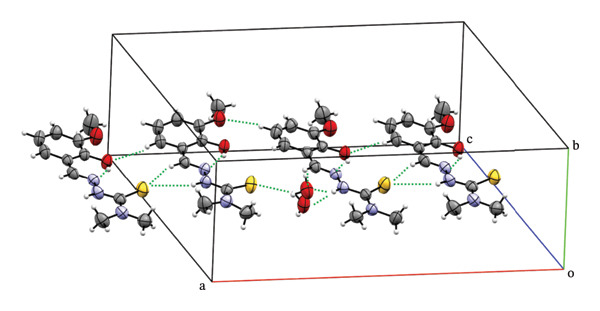
Packing diagram showing the molecule alignment along the *a* axis. Ellipsoids at 50% of probability, H atoms drawn as fixed‐size spheres with a diameter of 0.15 Å. The hydrogen bridges are drawn in green.

An ORTEP diagram is shown in Figure 3. All the bonds in the TSC moiety, except the thionic one, have a partial double‐bond character (Table S2) and lie in the same plane, due to bond conjugation, as observed in other TSCs [54]. The thiourea bond C1–N2 is in the unusual *Z* configuration, likely due to intramolecular interactions. Indeed, most reported TSC structures present none or only one substituent on the thioamide nitrogen (here N3), so that the hydrogen bridge between the iminic nitrogen and the hydrogen atom at this site promotes the typical *E* configuration. In H_2_L2, the presence of the methyl groups prevents this interaction; instead, a hydrogen bond is established between the iminic N1 atom and the phenolic O1‐H group, leading to the less hindered *Z* configuration. The TSC and water molecules are connected through a hydrogen‐bond framework that results in (i) two alternative positions of the water molecule and (ii) alignment of the molecules in rows along the crystallographic *a* axis (Figure 4, Figures S44 and S45), which may account for the observed tabular habit.

**FIGURE 5:**
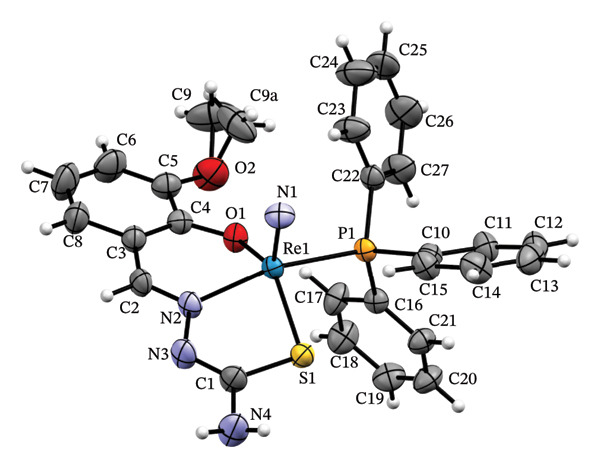
ORTEP diagram of the asymmetric unit of **Re1** crystal. Ellipsoids at 50% of probability, H atoms drawn as fixed‐size spheres with a diameter of 0.15 Å. Both alternate positions of the carbon atom (C9/C9a) in the disordered methoxy group are shown.

For **Re1**, red, prismatic and monoclinic (space group *P*2_1_ */n,* Figure S42) crystals were obtained by slow infusion of Et_2_O vapour in a CH_2_Cl_2_ solution at 4°C; the asymmetric unit (Figure 5) is made by one of the four efficiently packed complex molecules forming the unit cell.

**FIGURE 6:**
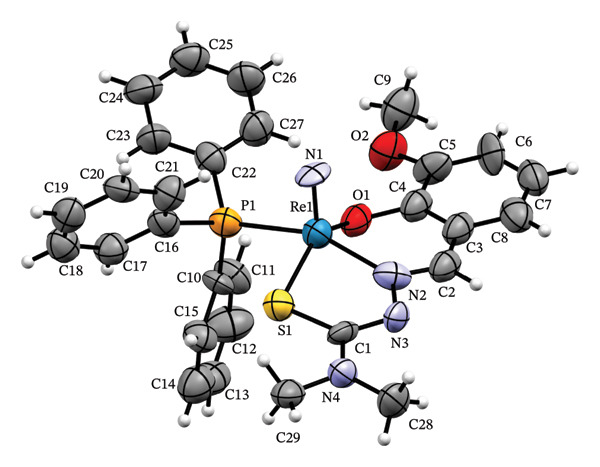
ORTEP diagram of C enantiomer of the asymmetric unit of **Re2** crystal. Ellipsoids at 30% of probability, H atoms drawn as fixed‐size spheres with a diameter of 0.15 Å.

For **Re2**, small, orange, tabular and triclinic crystals (space group Pī, Figure S42) were obtained by slow infusion of *n*‐hexane vapour in a CH_2_Cl_2_ solution at 4°C. The unit cell again incorporates four efficiently packed molecules; the asymmetric unit is made of an enantiomeric pair (Figures 6 and S46). Crystals had low diffracting ability, and the refinement proved difficult (see Supporting Information [available here]). However, the value of the final R indexes and the agreement with the other spectroscopic information and with elemental analysis make us hold that the reported structural parameters are acceptable.

In **Re1** and **Re2,** the metal environment has a distorted square pyramidal geometry, with the rhenium atom located 0.57 Å above the basal plane. The trigonality index *τ*5 is 0.34 for Re1 and 0.25 for Re2, the nitride ion takes apical position, and the Re‐N distance has triple bond character (1.654(2) Å and 1.588(15) Å for **Re1** and **Re2,** respectively).

The bis‐deprotonated TSC tridentate ligands (κ‐S,N,O) coordinate the metal centre and form 5‐ and6‐membered metallacycles showing *envelope* and *half-chair* conformations, respectively (the rhenium atom is in *endo* position in both cases). The phosphine P atom completes the basal plane. Small bond length variations to uncoordinated from coordinated ligands suggest a lower conjugation level of TSC moieties in bound ligands (Tables S2–S4 and reference [55]); in the proligands, the hydrazine N‐N bond exhibits a partial double‐bond character, whereas in the complexes, it is elongated and better described as a single bond (e.g., 1.367(4) Å in H_2_L1 vs. 1.415(3) Å in **Re1**). However, the lower level of conjugation hinted by bond lengths is not matched by a loss of planarity of thiourea nitrogen N4 (Tables S5 and S6).

Re‐P bond (2.39 Å) is shorter compared to a mean value of 2.43 Å found in similar rhenium–nitride arylphosphine complexes [56–58] (CSD‐CCDC consulted on March 14, 2025). This suggests that within the salicylthiosemicarbazonic coordination environment, rhenium requires a relatively strong interaction with the phosphorous atom to achieve stability. In contrast, a longer average metal‐P distance (2.48 Å) is found in transition metal nitrido‐complexes with alkylphosphines and a κ‐S,N,O donor set, indicating a weaker Re‐P bond [59–62] (CSD–CCDC consulted on March 14, 2025). This may explain why alkylphosphines are unable to stabilise a [ReN(L)(P)] complex, and consequently, why the [ReN(L)(PCN)] species could not be obtained.

### 3.4. Synthesis of Technetium‐99m Complexes

The technetium(V)‐99m nitride core is readily prepared from sodium pertechnetate‐99m reduction in the presence of an excess of succinic dihydrazide as nitride ion (N^3−^) donor; the reduction can be achieved using tin(II) chloride or a phosphine, as employed in this study [10, 12, 63, 64]. Accordingly, the complexes ^**99m**^ **Tc1-4**, with the general formula [^99m^Tc][TcN(L)P] (L = L1^2−^ or L2^2−^; P=PPh_3_ or PCN), were prepared following a two‐step synthesis. In the first step, the [^99m^Tc][TcN]^2+^ core was formed by reacting freshly eluted sodium pertechnetate‐99m with succinic dihydrazide and tin(II) chloride (Procedure A) or the relevant phosphine (Procedure B) for 15 min. Procedure A was conducted at autogenous pH and room temperature, whereas Procedure B required acidic pH and heating (100°C), consistent with the activation of phosphines as reductants [10]. In the second step, the technetium(V)‐99m nitride core was converted into the final complex by reacting with the relevant salicylthiosemicarbazonic ligand in alkaline ethanolic solution and phosphine in ethanolic acidic solution (Procedure A), or with the pertinent salicylthiosemicarbazonic ligand (Procedure B), in the presence of NaH_2_PO_4_ (0.2 M, pH 5) and adjusting the pH of the reaction mixture to 5 with sodium hydroxide. The reaction was heated at 100°C for 30 min.

The formation of the technetium‐99m nitride core is a well‐established reaction, using either tin(II) chloride or phosphines as reductants [64]; thus, the initial step of both procedures was not subjected to further optimisation. Instead, the second step, specific for the formation of the final mixed‐ligand complex, was optimised in terms of temperature, pH, incubation time and ligand amounts. Therefore, using the ^**99m**^ **Tc4** synthesis as a pilot reaction, and adhering to the amounts of phosphine and TSC reported in the Material and Methods, we found that a temperature lower than 100°C negatively impacts the RCY. A pH of 5 was found optimal, with both more acidic (2–4) and slightly alkaline pH (7 < pH < 8) values leading to lower RCYs. Highly alkaline pH (10–12) completely inhibited the labelling. At optimal pH and temperature, the reaction is completed after 30 min. Under these standardised conditions, the radiolabelling efficiency for Procedure A was assessed by scaling down the amount of PCN and H_2_L2, while for Procedure B, only the amount of H_2_L2 was reduced. The ligand concentration has a significant impact on the reaction yield: RCY ≥ 90% was obtained only using 5.2 and 4 µmol of phosphine and L, respectively.

The optimal pH/temperature/ligand amount conditions set up for ^**99m**^ **Tc4** were efficiently applied to the other ^99m^Tc compounds. In general, the RCYs achieved for the L1^2−^‐based complexes are higher than those of L2^2−^‐based ones, and among the tested situations, the PPh_3_/L2^2−^ combination of ligands is the least performant.

#### 3.4.1. Synthesis and Characterisation of Carrier‐Added Technetium‐99g/99m Complexes

**FIGURE 7:**
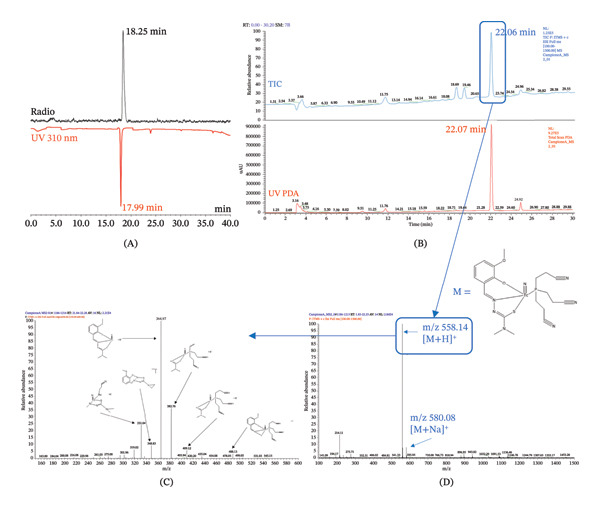
HPLC and LC–MS analyses of the SPE‐purified carrier‐added preparation of ^**99g/99m**^ **Tc4**. (A) radio/UV‐RP‐HPLC chromatogram. (B) TIC/UV‐RP‐HPLC chromatogram (mass spectrometer in positive mode). (C) ESI(+)–MS spectrum of the peak at 22.06 min in the TIC chromatogram. (D) MS^2^ spectrum of the peak at m/z 558.

For a better characterisation of technetium complexes obtained at the tracer level, we performed carrier‐added syntheses using a mixture of fresh eluted sodium pertechnetate‐99m and ammonium pertechnetate‐99g as starting reagents, as specified in the Material and Methods. Radio/UV‐HPLC was then used for a first check, to detect the formation of the relevant ^99g^Tc complexes by direct comparison of the UV and radio chromatograms of the labelling mixture. Then, LC–MS was used to assess the mass spectra of the ^99g^Tc complexes, performing MS/MS experiments to gather more information related to their possible structure. Carrier‐added syntheses gave the desired ^99g^Tc complexes, whose UV chromatograms perfectly match the radiochromatograms of the corresponding ^99m^Tc complexes, considering that the instrument detectors are in series, with the UV detector coming first. Each total ion current (TIC) chromatogram, in positive mode, displays a peak matching with the UV peak, revealing that ^**99g/99m**^ **Tc1-4** are ionisable species. The corresponding mass spectra show a base peak assignable to the molecular ion [M+H]^+^, with M consistent with the general formula [TcN(L) (P)] (L = L1^2−^ or L2^2−^; P=PCN or PPh_3_). The [M+Na]^+^ or [2M + H]^+^ ions are also detected. Finally, MS^2^ spectra of the base peaks display fragments which agree well with the given molecular structure. In Figure 7, the UV/radio/TIC chromatograms and MS/MS^2^ spectra for ^**99g/99m**^ **Tc4** are displayed for illustrative purposes; the others are disclosed in the Supporting Information (Figures S47–S49).

### 3.5. HPLC Comparison Between Technetium‐99m Complexes and the Corresponding Rhenium Complexes

**FIGURE 8:**
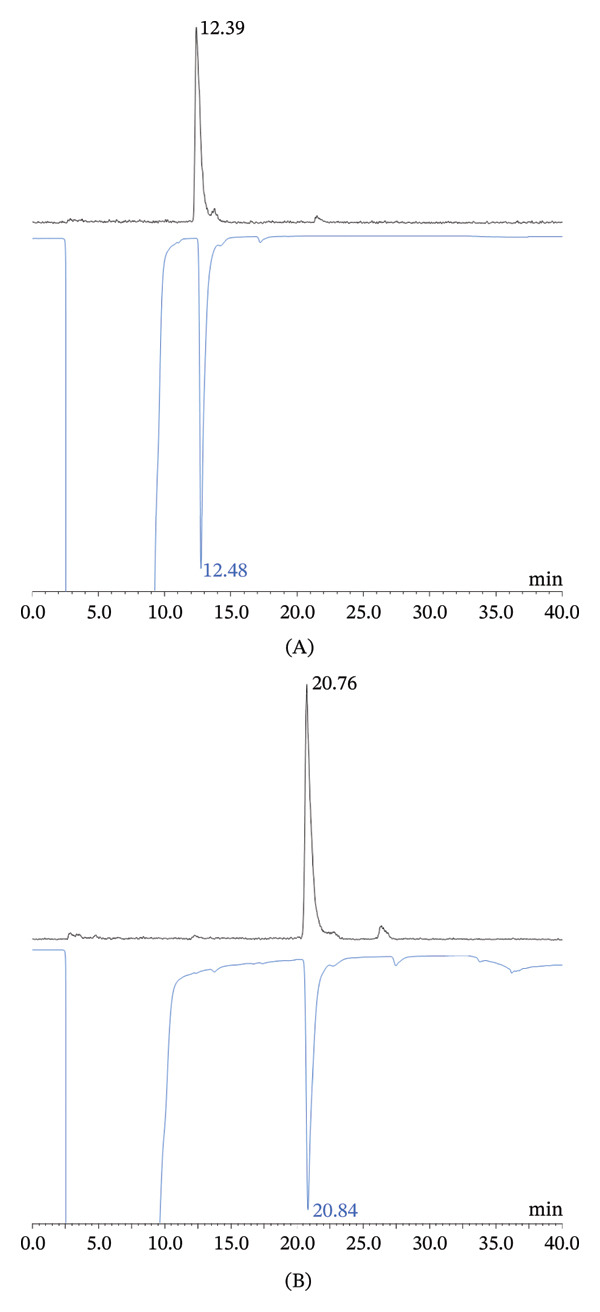
Radio/UV‐RP‐HPLC comparison of analogous technetium and rhenium complexes. (A) ^**99m**^ **Tc1** (black trace) and **Re1** (blue trace). (B) ^**99m**^ **Tc2** (black trace) and **Re2** (blue trace). The large out‐of‐scale peak between 0 and 10 min is given by the other compounds in the labelling mixture.

We also performed a chromatographic comparison (both TLC and radio/UV‐HPLC) of the corresponding **Re1**/^**99m**^ **Tc1** and **Re2**/^**99m**^ **Tc2** (see Materials and Methods). The almost full coincidence of the Rf and Rt values (see Table 1; Figure 8 displays the radio/UV‐RP‐HPLC comparison), together with the LC–MS results for the technetium carrier‐added products, makes it reasonable to suggest the identical coordination sphere for rhenium and technetium complexes.

### 3.6. Stability Studies of Technetium‐99m Complexes

Preliminary stability studies for radiocomplexes ^**99m**^ **Tc1-4** were performed by incubating SPE‐purified compound solutions in PBS, in the presence of cysteine and glutathione, and in human serum, at 37°C for 24 h. RCP of the complexes was checked by radio‐HPLC over time (0 vs 24 h), and results are summarised in Figure S50. Notably, PPh_3_‐based compounds exhibited a greater stability compared to the corresponding PCN‐containing complexes. After PBS dilution, RCPs measured at 24 h were in the range 80%–70% for ^**99m**^ **Tc1-2** and 40%–35% for ^**99m**^ **Tc3-4**. Stability experiments, involving small‐molecule challengers to the metal coordination sphere, showed a slower degradation rate of ^**99m**^ **Tc1-2** (RCP 60%–50% at 200 min) than ^**99m**^ **Tc3-4** complexes (RCP < 10% at 200 min). However, in all cases, no complexes were detected in the incubation mixtures at 24 h. After incubation in human serum, ^**99m**^ **Tc1-2** was sufficiently stable within 120 min of incubation (RCP 80%). On the contrary, a significant drop in RCP of ^**99m**^ **Tc3-4** was assessed at 80 min (50%–40%). At 24 h, ^**99m**^ **Tc1-4** complexes were degraded. For all the complexes, a high binding to protein was found (60%–70%), which reaches its maximum value after 80 min of incubation.

The HPLC chromatograms of the cysteine‐ and glutathione‐incubated samples show the formation of more hydrophilic species, consistent with the occurrence of transchelation. The chromatograms of PBS and human serum incubates display the formation of other more hydrophilic species; reoxidation of technetium to pertechnetate can be confidently excluded, as the nitride core is known to be highly stable; therefore, further studies are needed to elucidate whether the degradation products are a result of hydrolysis processes (likely involving the L ligands) or transchelation with some components of the media.

### 3.7. Remarks

On the basis of the preliminary ^99m^Tc‐labelling results, [^99m^Tc][TcN(L)P] (L = thiosemicarbazonate‐phenolate; P = monophosphine) emerges as a possible platform for the development of new technetium‐99m‐based radiotracers for radiopharmaceutical applications. However, improvements in labelling efficiency and complex stability are essential in view of the possible application in the development of tumour target‐specific agents. In this connection, the exploration of related tridentate TSC derivatives with varied donor atom sets, such as species bearing phosphinic groups in place of phenolic hydroxyls, is particularly appealing.

A notable divergence between technetium‐99m and rhenium chemistry is the lack of accessible PCN‐based rhenium analogues, suggesting that this ligand is less effective than PPh_3_ in stabilising the final complexes. Whether this behaviour reflects specific steric and electronic features of PCN or more general limitations of the monophosphine framework remains to be clarified. Further studies, including investigations at tracer‐level rhenium (e.g., rhenium‐188) and complementary computational analyses, will be important to distinguish between thermodynamic and kinetic contributions and to define the electronic requirements for stabilising the rhenium nitride core in this 3 + 1 coordination environment.

These aspects are directly relevant for identifying which other monodentate phosphines, beyond PPh_3_, can yield isostructural technetium and rhenium complexes at the tracer level, a prerequisite for the genuine translation of this platform to theranostic applications. In particular, establishing whether such isostructurality is limited to fully aromatic monodentate phosphines is essential, as this constraint might impact pharmacokinetic properties requiring compensatory structural modifications. From a radiopharmaceutical perspective, this divergence suggests that coordination environments optimised for technetium may not be directly transferable to rhenium, pointing to the need for metal‐specific ligand design strategies. Studies in this direction are currently underway.

## 4. Conclusion

In this study, we have shown that heteroleptic ‘3 + 1’ complexes of the type [M^V^N(L)P] (L = thiosemicarbazonate‐phenolate; P = monophosphine) can be successfully synthesised with M as either natural rhenium or technetium‐99m. The results demonstrate that the [TcN(L)P] system can be accessed under both carrier‐added and no‐carrier‐added conditions using different monophosphine ligands, supporting its potential as a platform for the development of technetium‐99m‐based radiotracers.

In contrast, the inability to obtain PCN‐based rhenium analogues highlights a difference in the stabilisation of the metal nitride core between technetium and rhenium under their respective experimental conditions. This behaviour points to the need for further studies to better define the applicability limits of this platform in theranostic contexts.

## Author Contributions

Conceptualisation: Cristina Bolzati, Nicola Salvarese. Data curation: Cristina Bolzati, Nicola Salvarese, Davide Lucchini, Alessandro Dolmella. Formal analysis: Cristina Bolzati, Nicola Salvarese, Davide Lucchini, Alessandro Dolmella, Carolina Gobbi. Funding acquisition: Cristina Bolzati. Investigation: Davide Lucchini, Carolina Gobbi, Nicola Salvarese. Methodology: Cristina Bolzati, Nicola Salvarese, Davide Lucchini, Marco Baron. Project administration: Cristina Bolzati. Resources: Cristina Bolzati, Dominga Rogolino, Mauro Carcelli. Supervision: Cristina Bolzati, Nicola Salvarese, Marco Baro. Validation: Nicola Salvarese. Writing–original draft: Nicola Salvarese, Davide Lucchini, Alessandro Dolmella, Cristina Bolzati. Writing–review and editing: all authors.

## Funding

The authors also acknowledge Associazione Italiana per la Ricerca sul Cancro (AIRC) (AIRC, IG 2020 ID 24528), the International Atomic Energy Agency (CRP F22077: Development of new generation of Tc‐99m kits) and the University of Parma (‘Bando di Ateneo 2023 per la ricerca’) for financial support. Dominga Rogolino and Mauro Carcelli are grateful for the use of facilities acquired in the framework of the COMP‐HUB and COMP‐R initiatives, funded by the ‘Departments of Excellence’ programme of the Italian Ministry for University and Research (MUR, 2023–2027).

## Conflicts of Interest

The authors declare no conflicts of interest.

## Supporting Information

Additional supporting information can be found online in the Supporting Information section.

## Supporting information


**Supporting Information** Supporting Information is available as separate files. This information provides additional data related to the study presented in the main manuscript, essential for providing further evidence for the chemical identities of the obtained compounds and supporting the conclusions. It includes a detailed pdf file containing the following material: ESI–MS, FT‐IR, one‐dimensional ^1^H, ^13^C, ^31^P NMR and two‐dimensional NMR spectra of rhenium complexes **Re0**, **Re1** and **Re2**; crystallographic data and diagrams for **H**_**2**_ **L2** and rhenium complexes **Re1** and **Re2**; radio/UV‐HPLC and LC–MS data for ^**99g/99m**^ **Tc1-3**; stability of ^**99m**^ **Tc1-4** in phosphate buffer saline, cysteine 1 mM, glutathione 1 mM and human serum type AB; experimental details of the attempts to obtain PCN‐based rhenium complexes. Crystallographic data in the form of .cif files (file names: ‘32_xx1_twin_nowob_nofried_twin1_hklf4_088.cif’, ‘33_DC_auto.cif’ and ‘test_mr24fp.cif’), and checkcif as PDF files (file names: ‘32_xx1_twin_nowob_nofried_twin1_hklf4_088 cifreport.pdf’, ‘33_DC_auto_cifreport.pdf’ and ‘test_mr24fp_cifreport.pdf’), are also included.

## Data Availability

The data supporting this article have been included as part of the Supporting Information File (pdf). Crystallographic data are also available in the form of .cif files as supporting information (file names: ‘32_xx1_twin_nowob_nofried_twin1_hklf4_088.cif’, ‘33_DC_auto.cif’ and ‘test_mr24fp.cif’), checkcif are included as PDF files (file names: ‘32_xx1_twin_nowob_nofried_twin1_hklf4_088 cifreport.pdf’, ‘33_DC_auto_cifreport.pdf’ and ‘test_mr24fp_cifreport.pdf’). Crystallographic data for **H**_**2**_ **L2**, **Re1** and **Re2** have been deposited at the CCDC under 2346537, 2345940 and 2346950, respectively.
