# Supplementary material for: Synthesis, Characterisation and Structural Analysis of Rhenium and Technetium Nitride Complexes With Tridentate Thiosemicarbazone‐Phenols and Phosphine Ligands: Potential Applications in Technetium‐99m Radiotracer Development
Source: Bioinorg Chem Appl. 2026 Jul 22;2026:3202767. doi: 10.1155/bca/3202767 (PMC13390190; doi:10.1155/bca/3202767)

checkCIF/PLATON report

Structure factors have been supplied for datablock(s) 33_dc_auto

THIS REPORT IS FOR GUIDANCE ONLY. IF USED AS PART OF A REVIEW PROCEDURE FOR PUBLICATION, IT SHOULD NOT REPLACE THE EXPERTISE OF AN EXPERIENCED CRYSTALLOGRAPHIC REFEREE.

No syntax errors found. CIF dictionary Interpreting this report

Datablock: 33_dc_auto

Bond precision:	C-C = 0.0052 A		Wavelength=0.71073
Cell:	a=9.6608(3)	b=16.7436(6)	c=16.2967(5)
	alpha=90	beta=100.231(3)	gamma=90
Temperature:	293 K		

	Calculated	Reported
Volume	2594.18(15)	2594.18(15)
Space group	P 21/n	P 1 21/n 1
Hall group	-P 2yn	-P 2yn
Moiety formula	C27 H24 N4 O2 P Re S	C27 H24 N4 O2 P Re S
Sum formula	C27 H24 N4 O2 P Re S	C27 H24 N4 O2 P Re S
Mr	685.74	685.73
Dx,g cm-3	1.756	1.756
Z	4	4
Mu (mm-1)	4.859	4.859
F000	1344.0	1344.0
F000'	1341.33	
h,k,lmax	14,24,24	14,24,23
Nref	8840	8411
Tmin,Tmax	0.421,0.615	0.303,1.000
Tmin'	0.230	

Correction method= # Reported T Limits: Tmin=0.303 Tmax=1.000
AbsCorr = MULTI-SCAN

Data completeness= 0.951 Theta(max)= 31.767

R(reflections)= 0.0294(7249) wR2(reflections)= 0.0662(8411)

S = 1.042 Npar= 338

The following ALERTS were generated. Each ALERT has the format
test-name_ALERT_alert-type_alert-level.
Click on the hyperlinks for more details of the test.

Alert level C

```
PLAT420_ALERT_2_C D-H Bond Without Acceptor  N4       --H4B      .     Please Check
PLAT906_ALERT_3_C Large K Value in the Analysis of Variance ......      3.078 Check
PLAT911_ALERT_3_C Missing FCF Refl Between Thmin & STh/L=    0.600          4 Report
                0  0 14,   2  1 16,   0  0 18,  -1  0 19,
PLAT971_ALERT_2_C Check Calcd Resid. Dens.  0.78Ang From Re1             1.82 eA-3
PLAT971_ALERT_2_C Check Calcd Resid. Dens.  0.83Ang From Re1             1.52 eA-3
```

Alert level G

```
PLAT007_ALERT_5_G Number of Unrefined Donor-H Atoms .............           2 Report
              H4A   H4B
PLAT012_ALERT_1_G No       _shelx_res_checksum Found in CIF ......     Please Check
PLAT187_ALERT_4_G The CIF-Embedded .res File Contains RIGU Records          1 Report
PLAT199_ALERT_1_G Reported _cell_measurement_temperature ..... (K)        293 Check
PLAT200_ALERT_1_G Reported   _diffrn_ambient_temperature ..... (K)        293 Check
PLAT232_ALERT_2_G Hirshfeld Test Diff (M-X)  Re1      --S1       .        5.8 s.u.
PLAT301_ALERT_3_G Main Residue  Disorder .............(Resd    1)          3% Note
PLAT720_ALERT_4_G Number of Unusual/Non-Standard Labels ..........          3 Note
              H9AA    H9AB    H9AC
PLAT912_ALERT_4_G Missing # of FCF Reflections Above STh/L=  0.600        419 Note
PLAT933_ALERT_2_G Number of HKL-OMIT Records in Embedded .res File          7 Note
               -1  0 19,   0  0 14,   0  0 18,   1  0 19,   2  1 16,   2  1 20,
                3  3 20,
PLAT969_ALERT_5_G The 'Henn et al.' R-Factor-gap value ..........        3.46 Note
              Predicted wR2: Based on SigI**2  1.91 or SHELX Weight  6.48
PLAT978_ALERT_2_G Number C-C Bonds with Positive Residual Density.          1 Info
```

0 **ALERT level A** = Most likely a serious problem - resolve or explain
0 **ALERT level B** = A potentially serious problem, consider carefully
5 **ALERT level C** = Check. Ensure it is not caused by an omission or oversight
12 **ALERT level G** = General information/check it is not something unexpected

3 ALERT type 1 CIF construction/syntax error, inconsistent or missing data
6 ALERT type 2 Indicator that the structure model may be wrong or deficient
3 ALERT type 3 Indicator that the structure quality may be low
3 ALERT type 4 Improvement, methodology, query or suggestion
2 ALERT type 5 Informative message, check

It is advisable to attempt to resolve as many as possible of the alerts in all categories. Often the minor alerts point to easily fixed oversights, errors and omissions in your CIF or refinement strategy, so attention to these fine details can be worthwhile. In order to resolve some of the more serious problems it may be necessary to carry out additional measurements or structure refinements. However, the purpose of your study may justify the reported deviations and the more serious of these should normally be commented upon in the discussion or experimental section of a paper or in the "special_details" fields of the CIF. checkCIF was carefully designed to identify outliers and unusual parameters, but every test has its limitations and alerts that are not important in a particular case may appear. Conversely, the absence of alerts does not guarantee there are no aspects of the results needing attention. It is up to the individual to critically assess their own results and, if necessary, seek expert advice.

Publication of your CIF in IUCr journals

A basic structural check has been run on your CIF. These basic checks will be run on all CIFs submitted for publication in IUCr journals (*Acta Crystallographica*, *Journal of Applied Crystallography*, *Journal of Synchrotron Radiation*); however, if you intend to submit to *Acta Crystallographica Section C* or *E* or *IUCrData*, you should make sure that full publication checks are run on the final version of your CIF prior to submission.

Publication of your CIF in other journals

Please refer to the *Notes for Authors* of the relevant journal for any special instructions relating to CIF submission.

PLATON version of 06/01/2024; check.def file version of 05/01/2024

Datablock 33_dc_auto - ellipsoid plot

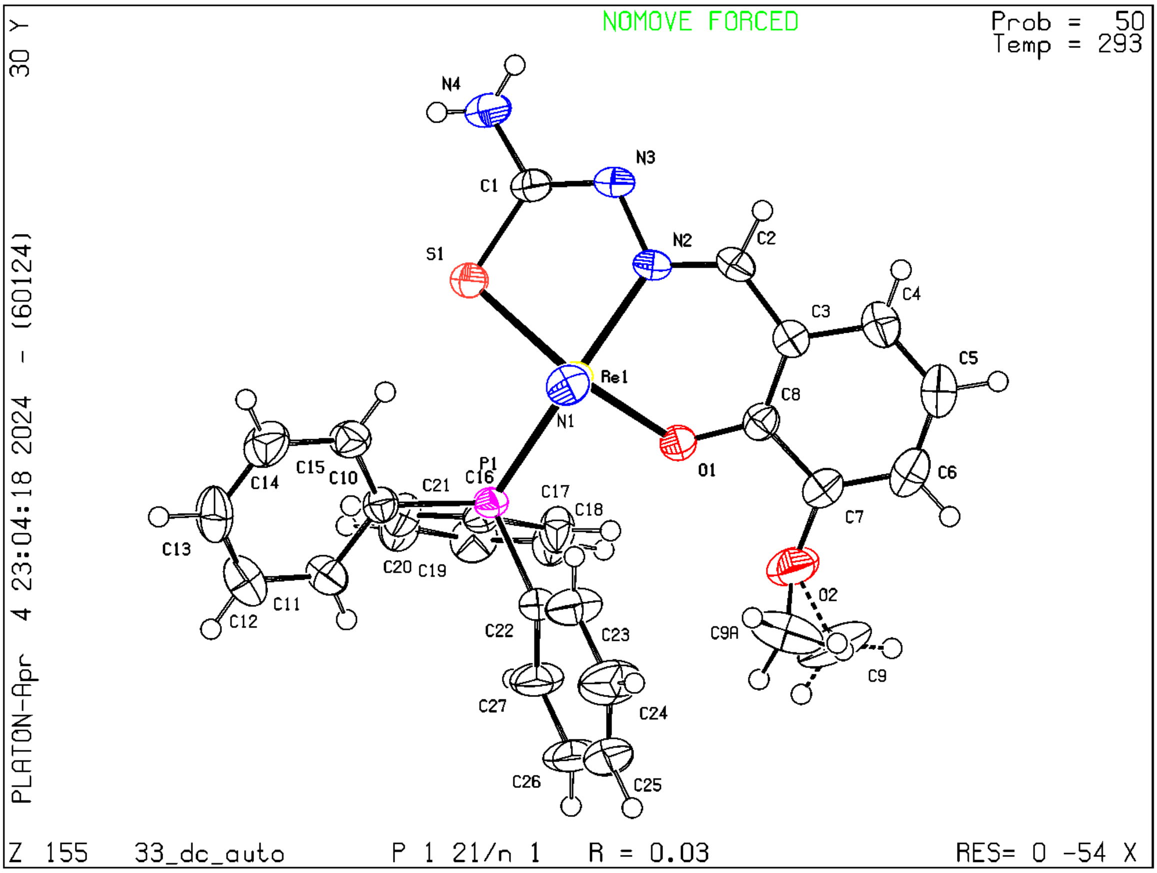

Supplement: Supplementary file 1 — Supporting Information Supporting Information is available as separate files. This information provides additional data related to the study presented in the main manuscript, essential for providing further evidence for the chemical identities of the obtained compounds and supporting the conclusions. It includes a detailed pdf file containing the following material: ESI–MS, FT‐IR, one‐dimensional 1H, 13C, 31P NMR and two‐dimensional NMR spectra of rhenium complexes Re0, Re1 and Re2; crystallographic data and diagrams for H 2 L2 and rhenium complexes Re1 and Re2; radio/UV‐HPLC and LC–MS data for 99g/99m Tc1-3; stability of 99m Tc1-4 in phosphate buffer saline, cysteine 1 mM, glutathione 1 mM and human serum type AB; experimental details of the attempts to obtain PCN‐based rhenium complexes. Crystallographic data in the form of .cif files (file names: ‘32_xx1_twin_nowob_nofried_twin1_hklf4_088.cif’, ‘33_DC_auto.cif’ and ‘test_mr24fp.cif’), and checkcif as PDF files (file names: ‘32_xx1_twin_nowob_nofried_twin1_hklf4_088 cifreport.pdf’, ‘33_DC_auto_cifreport.pdf’ and ‘test_mr24fp_cifreport.pdf’), are also included. [file BCA-2026-3202767-s001.zip › 33_DC_auto_cifreport.pdf]
