# Supplementary material for: Synthesis, Characterisation and Structural Analysis of Rhenium and Technetium Nitride Complexes With Tridentate Thiosemicarbazone‐Phenols and Phosphine Ligands: Potential Applications in Technetium‐99m Radiotracer Development
Source: Bioinorg Chem Appl. 2026 Jul 22;2026:3202767. doi: 10.1155/bca/3202767 (PMC13390190; doi:10.1155/bca/3202767)

checkCIF/PLATON report

Structure factors have been supplied for datablock(s) test_mr24fp

THIS REPORT IS FOR GUIDANCE ONLY. IF USED AS PART OF A REVIEW PROCEDURE FOR PUBLICATION, IT SHOULD NOT REPLACE THE EXPERTISE OF AN EXPERIENCED CRYSTALLOGRAPHIC REFEREE.

No syntax errors found. CIF dictionary Interpreting this report

Datablock: test_mr24fp

```
Bond precision:   C-C = 0.0031 A           Wavelength=0.71073

Cell:            a=20.8659(5)     b=7.24227(16)        c=25.5024(5)
                 alpha=90         beta=90.3003(19)     gamma=90
Temperature:     293 K

                Calculated                    Reported
Volume          3853.78(15)                   3853.78(15)
Space group     P 21/n                        P 1 21/n 1
Hall group      -P 2yn                        -P 2yn
Moiety formula  3(C11 H15 N3 O2 S), H2 O      3(C11 H15 N3 O2 S), H2 O
Sum formula     C33 H47 N9 O7 S3              C33 H47 N9 O7 S3
Mr              777.98                        777.97
Dx,g cm-3       1.341                         1.341
Z               4                             4
Mu (mm-1)       0.250                         0.250
F000            1648.0                        1648.0
F000'           1650.12
h,k,lmax        24,8,29                       24,8,28
Nref            6178                          5289
Tmin,Tmax       0.965,0.998                   0.863,1.000
Tmin'           0.928

Correction method= # Reported T Limits: Tmin=0.863 Tmax=1.000
AbsCorr = MULTI-SCAN

Data completeness= 0.856           Theta(max)= 24.166

                                                      wR2(reflections)=
R(reflections)= 0.0386( 4023)                         0.1031( 5289)

S = 1.051                    Npar= 497
```

The following ALERTS were generated. Each ALERT has the format
test-name_ALERT_alert-type_alert-level.
Click on the hyperlinks for more details of the test.

Alert level A

```
PLAT029_ALERT_3_A _diffrn_measured_fraction_theta_full value Low .      0.856 Why?
```

Author Response: The selected item was a very thin plate of a merely organic material. The intensities of diffraction peaks markedly declined at theta values around 20 deg, where also the F2/sigmaF2 ratio lowered below 3.0. This situation prevented us to explore higher theta values. Despite this, the analysis of electron density maps does not show unassigned features and the proposed solution looks acceptable. This accounts also for the Alert B below.

Alert level B

```
PLAT911_ALERT_3_B Missing FCF Refl Between Thmin & STh/L=    0.576        877 Report
                0  8  0,   1  8  0,   2  8  0,   3  8  0,   4  8  0,   5  8  0,
                6  8  0,   8  7  0,  10  7  0,  11  7  0,  12  7  0,  13  6  0,
               13  7  0,  14  6  0,  15  6  0,  16  5  0,  16  6  0,  17  5  0,
               18  5  0,  19  5  0,  20  4  0,  21  4  0,  23  2  0, -23  2  1,
              -21  4  1, -20  4  1, -19  5  1, -18  5  1, -17  5  1, -16  6  1,
              -15  6  1, -14  6  1, -13  6  1, -13  7  1, -12  7  1, -11  7  1,
              -10  7  1,  -8  7  1,  -6  8  1,  -5  8  1,  -4  8  1,  -3  8  1,
               -2  8  1,  -1  8  1,   0  8  1,   1  8  1,   2  8  1,   3  8  1,
                4  8  1,   5  8  1,   6  8  1,   8  7  1,  10  7  1,  11  7  1,
               12  7  1,  13  6  1,  13  7  1,  14  6  1,  15  6  1,  16  5  1,
               16  6  1,  17  5  1,  18  5  1,  19  5  1,  21  4  1,  23  2  1,
              -21  4  2, -20  4  2, -19  5  2, -18  5  2, -17  5  2, -16  6  2,
              -15  6  2, -14  6  2, -13  6  2, -12  7  2, -11  7  2, -10  7  2,
               -7  7  2,  -6  8  2,  -5  8  2,  -4  8  2,  -3  8  2,  -2  8  2,
               -1  8  2,   0  8  2,   1  8  2,   4  8  2,   5  8  2,   6  8  2,
                9  7  2,  10  7  2,  11  7  2,  12  7  2,  14  6  2,  15  6  2,
```

Author Response: See answer for Alert A above.

Alert level C

```
THETM01_ALERT_3_C  The value of sine(theta_max)/wavelength is less than 0.590
            Calculated sin(theta_max)/wavelength =    0.5760
PLAT420_ALERT_2_C D-H Bond Without Acceptor  N2A      --H3A      .     Please Check
PLAT420_ALERT_2_C D-H Bond Without Acceptor  N2B      --H3B      .     Please Check
PLAT906_ALERT_3_C Large K Value in the Analysis of Variance ......      4.383 Check
```

Alert level G

```
PLAT005_ALERT_5_G No Embedded Refinement Details Found  in the CIF     Please Do !
PLAT007_ALERT_5_G Number of Unrefined Donor-H Atoms .............         10 Report
```

```
              H1    H3    H1A   H3A   H1B   H3B   H1WA  H1WB  H1WC  H1WD
PLAT199_ALERT_1_G Reported _cell_measurement_temperature ..... (K)        293 Check
PLAT200_ALERT_1_G Reported   _diffrn_ambient_temperature ..... (K)        293 Check
PLAT302_ALERT_4_G Anion/Solvent/Minor-Residue Disorder (Resd    4)       100% Note
PLAT302_ALERT_4_G Anion/Solvent/Minor-Residue Disorder (Resd    5)       100% Note
PLAT304_ALERT_4_G Non-Integer Number of Atoms in ..... (Resd    4)       1.59 Check
PLAT304_ALERT_4_G Non-Integer Number of Atoms in ..... (Resd    5)       1.41 Check
PLAT720_ALERT_4_G Number of Unusual/Non-Standard Labels .........          11 Note
              H9AC    H9AA    H9AB    H9BC    H9BA    H9BB    H1WA    H1WB
              O1W'    H1WC    H1WD
PLAT910_ALERT_3_G Missing # of FCF Reflection(s) Below Theta(Min).          2 Note
               -1  0  1,   1  0  1,
PLAT953_ALERT_1_G Reported (CIF) and Actual (FCF) Hmax Differ by .          1 Units
PLAT969_ALERT_5_G The 'Henn et al.' R-Factor-gap value ..........      4.393 Note
              Predicted wR2: Based on SigI**2  2.35 or SHELX Weight  9.81
PLAT978_ALERT_2_G Number C-C Bonds with Positive Residual Density.          0 Info
```

---

1 **ALERT level A** = Most likely a serious problem - resolve or explain
1 **ALERT level B** = A potentially serious problem, consider carefully
4 **ALERT level C** = Check. Ensure it is not caused by an omission or oversight
13 **ALERT level G** = General information/check it is not something unexpected

3 ALERT type 1 CIF construction/syntax error, inconsistent or missing data
3 ALERT type 2 Indicator that the structure model may be wrong or deficient
5 ALERT type 3 Indicator that the structure quality may be low
5 ALERT type 4 Improvement, methodology, query or suggestion
3 ALERT type 5 Informative message, check

It is advisable to attempt to resolve as many as possible of the alerts in all categories. Often the minor alerts point to easily fixed oversights, errors and omissions in your CIF or refinement strategy, so attention to these fine details can be worthwhile. In order to resolve some of the more serious problems it may be necessary to carry out additional measurements or structure refinements. However, the purpose of your study may justify the reported deviations and the more serious of these should normally be commented upon in the discussion or experimental section of a paper or in the "special_details" fields of the CIF. checkCIF was carefully designed to identify outliers and unusual parameters, but every test has its limitations and alerts that are not important in a particular case may appear. Conversely, the absence of alerts does not guarantee there are no aspects of the results needing attention. It is up to the individual to critically assess their own results and, if necessary, seek expert advice.

Publication of your CIF in IUCr journals

A basic structural check has been run on your CIF. These basic checks will be run on all CIFs submitted for publication in IUCr journals (*Acta Crystallographica*, *Journal of Applied Crystallography*, *Journal of Synchrotron Radiation*); however, if you intend to submit to *Acta Crystallographica Section C* or *E* or *IUCrData*, you should make sure that full publication checks are run on the final version of your CIF prior to submission.

Publication of your CIF in other journals

Please refer to the *Notes for Authors* of the relevant journal for any special instructions relating to CIF submission.

PLATON version of 02/02/2025; check.def file version of 02/02/2025

Datablock test_mr24fp - ellipsoid plot

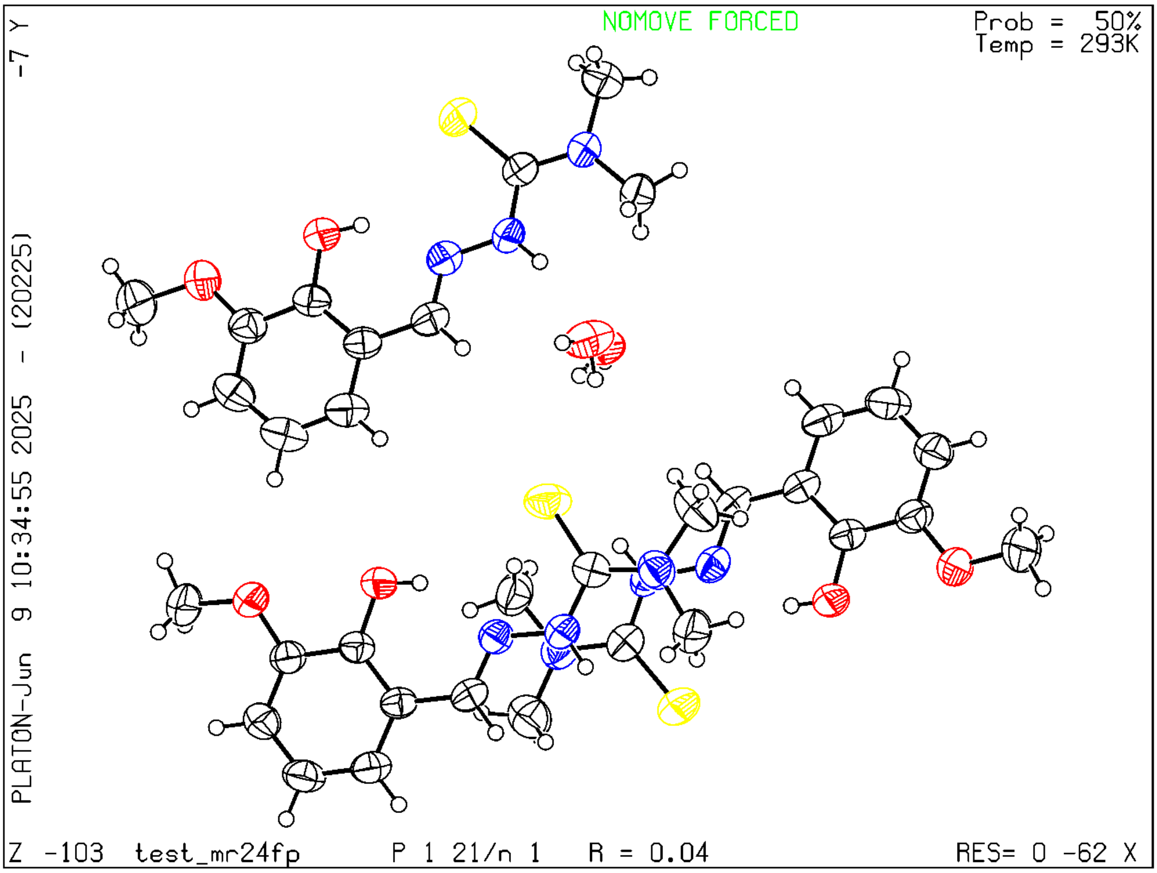

Supplement: Supplementary file 1 — Supporting Information Supporting Information is available as separate files. This information provides additional data related to the study presented in the main manuscript, essential for providing further evidence for the chemical identities of the obtained compounds and supporting the conclusions. It includes a detailed pdf file containing the following material: ESI–MS, FT‐IR, one‐dimensional 1H, 13C, 31P NMR and two‐dimensional NMR spectra of rhenium complexes Re0, Re1 and Re2; crystallographic data and diagrams for H 2 L2 and rhenium complexes Re1 and Re2; radio/UV‐HPLC and LC–MS data for 99g/99m Tc1-3; stability of 99m Tc1-4 in phosphate buffer saline, cysteine 1 mM, glutathione 1 mM and human serum type AB; experimental details of the attempts to obtain PCN‐based rhenium complexes. Crystallographic data in the form of .cif files (file names: ‘32_xx1_twin_nowob_nofried_twin1_hklf4_088.cif’, ‘33_DC_auto.cif’ and ‘test_mr24fp.cif’), and checkcif as PDF files (file names: ‘32_xx1_twin_nowob_nofried_twin1_hklf4_088 cifreport.pdf’, ‘33_DC_auto_cifreport.pdf’ and ‘test_mr24fp_cifreport.pdf’), are also included. [file BCA-2026-3202767-s001.zip › test_mr24fp_cifreport.pdf]
