# Supplementary material for: Synthesis, Characterisation and Structural Analysis of Rhenium and Technetium Nitride Complexes With Tridentate Thiosemicarbazone‐Phenols and Phosphine Ligands: Potential Applications in Technetium‐99m Radiotracer Development
Source: Bioinorg Chem Appl. 2026 Jul 22;2026:3202767. doi: 10.1155/bca/3202767 (PMC13390190; doi:10.1155/bca/3202767)
Supplement: Supplementary file 1 — Supporting Information Supporting Information is available as separate files. This information provides additional data related to the study presented in the main manuscript, essential for providing further evidence for the chemical identities of the obtained compounds and supporting the conclusions. It includes a detailed pdf file containing the following material: ESI–MS, FT‐IR, one‐dimensional 1H, 13C, 31P NMR and two‐dimensional NMR spectra of rhenium complexes Re0, Re1 and Re2; crystallographic data and diagrams for H 2 L2 and rhenium complexes Re1 and Re2; radio/UV‐HPLC and LC–MS data for 99g/99m Tc1-3; stability of 99m Tc1-4 in phosphate buffer saline, cysteine 1 mM, glutathione 1 mM and human serum type AB; experimental details of the attempts to obtain PCN‐based rhenium complexes. Crystallographic data in the form of .cif files (file names: ‘32_xx1_twin_nowob_nofried_twin1_hklf4_088.cif’, ‘33_DC_auto.cif’ and ‘test_mr24fp.cif’), and checkcif as PDF files (file names: ‘32_xx1_twin_nowob_nofried_twin1_hklf4_088 cifreport.pdf’, ‘33_DC_auto_cifreport.pdf’ and ‘test_mr24fp_cifreport.pdf’), are also included. [file BCA-2026-3202767-s001.zip › bca3202767-suppinfo-def.pdf]

Supporting Information for

Synthesis, Characterization, and Structural Analysis of Rhenium and Technetium Nitride Complexes with Tridentate Thiosemicarbazone-Phenols and Phosphine Ligands: Potential Applications in Technetium-99m Radiotracer Development.

Nicola Salvarese,*[a1] Davide Lucchini,*[b1] Carolina Gobbi,[a] Marco Baron,[b] Alessandro Dolmella,[c] Dominga Rogolino,[d] Mauro Carcelli [d] and Cristina Bolzati*[a1]

[a] Institute of Condensed Matter Chemistry and Energy Technologies – National Research Council, Corso Stati Uniti 4, 35127 Padua, Italy. E-mail: nicola.salvarese@cnr.it, cristina.bolzati@cnr.it.

[b] Department of Chemical Sciences, University of Padua, Via F. Marzolo 1, 35131 Padua, Italy, E-mail: davide.lucchini@phd.unipd.it

[c] Department of Chemistry, Life Sciences and Environmental Sustainability, University of Parma, Parco Area Delle Scienze 17/A, Parma, 43124, Italy

[d] Department of Pharmaceutical and Pharmacological Sciences, University of Padua, Via F. Marzolo 5, 35131 Padua, Italy

*Corresponding authors. Nicola Salvarese, nicola.salvarese@cnr.it); Davide Lucchini, davide.lucchini@phd.unipd.it; Cristina Bolzati, cristina.bolzati@cnr.it.

[1] Present address: Department of Pharmaceutical and Pharmacological Sciences, University of Padua, Via F. Marzolo 5, 35131 Padua, Italy.

INDEX

INDEX 2

FIGURES 5

Figure S1. ESI(+)-MS analysis of **Re0**. Full from m/z 250 to m/z 1000. 6

Figure S2. ESI(+)-MS analysis of **Re0**. Up: zoom scan, profile mode, from m/z 612 to m/z 652. Bottom: zoom scan, profile mode, from m/z 648 to m/z 688. 7

Figure S3. ESI(+)-MS analysis of **Re0**. Up: zoom scan, profile mode, from m/z 612 to m/z 652. Bottom: simulation for $C_{18}H_{24}ClN_7P_2Re^+$, $[M–Cl]^+$ (M = $C_{18}H_{24}Cl_2N_7P_2Re$). 8

Figure S4. ESI(+)-MS analysis of **Re0**. Up: zoom scan, profile mode, from m/z 612 to m/z 652. Bottom: simulation for $C_{18}H_{26}ClN_7OP_2Re^+$, $[M+H_2O–Cl]^+$ (M = $C_{18}H_{24}Cl_2N_7P_2Re$). 9

Figure S5. ESI(+)-MS analysis of **Re0**. Up: zoom scan, profile mode, from m/z 648 to m/z 688. Bottom: simulation for a mixture of $C_{18}H_{24}Cl_2N_7P_2Re^{+\cdot}$ ($[M]^{+\cdot}$, m/z 657) and $C_{18}H_{25}Cl_2N_7P_2Re^+$ ($[M+H]^+$, m/z 658), ratio 0.3:1 10

Figure S6. ESI(+)-MS analysis of **Re0**. Up: zoom scan, profile mode, from m/z 648 to m/z 688. Bottom: simulation for a mixture of $C_{18}H_{26}Cl_2N_7OP_2Re^{+\cdot}$ ($[M+H_2O]^{+\cdot}$) and $C_{18}H_{27}Cl_2N_7OP_2Re^+$ ($[M+H_2O+H]^+$), ratio 1:1. 11

Figure S7. ESI(+)-MS analysis of **Re0**. ESI(+)-MS^2 (up) and ESI(+)-MS^3 (bottom), ion at m/z 658 12

Figure S8. Hypothetic fragmentation pathway for the ion at m/z 658 according to the MS^n spectra in Figure S7. 13

Figure S9. ESI(+)-MS analysis of **Re0**. ESI(+)-MS^2 (up) and ESI(+)-MS^3 (bottom), ion at m/z 640 14

Figure S10. Hypothetic fragmentation pathway for the ion at m/z 640 according to the MS^n spectra in Figure S9. 15

Figure S11. ESI(+)-MS analysis of **Re0**. ESI(+)-MS^2 (up), ESI(+)-MS^3 (middle) and ESI(+)-MS^4 (bottom), ion at m/z 622 16

Figure S12. Hypothetic fragmentation pathway for the ion at m/z 622 according to the MS^n spectra in Figure S11. 17

Figure S13. ESI(+)-MS analysis of **Re1**. Full from m/z 150 to m/z 2000. 18

Figure S14. ESI(+)-MS analysis of **Re1**. Simulation for $C_{27}H_{24}N_4O_2PSRe+Na^+$, $[M+Na]^+$ (M = $C_{27}H_{24}N_4O_2PSRe$). 19

Figure S15. ESI(+)-MS analysis of **Re2**. Full from m/z 150 to m/z 2000. 20

Figure S16. ESI(+)-MS analysis of **Re2**. Simulation for $C_{29}H_{28}N_4O_2PSRe^+$, $[M]^{+\cdot}$ (M = $C_{29}H_{28}N_4O_2PSRe$). 21

Figure S17. FT-IR spectrum (in KBr) of **Re0**. 22

Figure S18. Blue: FT-IR spectrum (in KBr) of **Re0**. Black: FT-IR spectrum (in KBr) of PCN. . 23

Figure S19. FT-IR spectrum (in KBr) of **Re1**. 24

Figure S20. Blue: FT-IR spectrum (in KBr) of **Re1**. Red: FT-IR spectrum (in KBr) of **H_2L1**....25
Figure S21. FT-IR spectrum (in KBr) of **Re2**. 26
Figure S22. Blue: FT-IR spectrum (in KBr) of **Re2**. Red: FT-IR spectrum (in KBr) of **H_2L2**....27
Figure S23. $^{31}P\{^1H\}$ NMR spectrum in CD_3CN of **Re0**. 28
Figure S24. ^1H NMR spectrum in CD3CN of **Re0**. 29
Figure S25. $^{13}C\{^1H\}$ NMR spectrum in CD_3CN of **Re0**. 30
Figure S26. ^1H-^{13}C HMBC in CD_3CN of **Re0**. 31
Figure S27. $^{31}P\{^1H\}$ NMR spectrum in CD_2Cl_2 of **Re1**. 32
Figure S28. ^1H NMR spectrum in CD_2Cl_2 of **Re1**. 33
Figure S29. $^{13}C\{^1H\}$ NMR spectrum in CD_2Cl_2 of **Re1**. 34
Figure S30. ^1H-^{13}C HMQC in CD_2Cl_2 of **Re1**. 35
Figure S31. ^1H-^{13}C HMBC in CD_2Cl_2 of **Re1**. 36
Figure S32. ^1H-^{31}P HMBC in CD_2Cl_2 of **Re1**. 37
Figure S33. $^{31}P\{^1H\}$ NMR spectrum in CD_2Cl_2 of **Re2**. 38
Figure S34. ^1H NMR spectrum in CD_2Cl_2 of **Re2**. 39
Figure S35. $^{13}C\{^1H\}$ NMR spectrum in CD_2Cl_2 of **Re2**. 40
Figure S36. ^1H-^{13}C HSQC in CD_2Cl_2 of **Re2**. 41
Figure S37. ^1H-^{13}C HMBC in CD_2Cl_2 of **Re2**. 42
Figure S38. ^1H-^{31}P HMBC in CD_2Cl_2 of **Re2**. 43
Figure S39. $^{31}P\{^1H\}$ NMR spectrum in CD_3CN of PCN. 44
Figure S40. ^1H NMR spectrum in CD_2Cl_2 of **H_2L2**. 45
Figure S41. Comparison between ^1H NMR spectra (in CD_2Cl_2) of **H_2L2** (red) and **Re2** (blue). Top: 11.6 ppm – 6.6 ppm region; green lines underline the acidic protons of **H_2L2** (absent in Re2), the arrow indicates the downfield shift of the azomethinic proton signal in **Re2**. Bottom: 5.6 – 2.6 ppm region; the arrow indicates the upfield shift of the methoxyl proton signal in **Re2**. 46
Figure S42. Crystals used in the analysis reported in the main text. Left to right: **H_2L2**, **Re1**, **Re2**. Capillary diameter of 100 μm. 47
Figure S43. ORTEP diagram of the asymmetric unit of **H_2L2**. Ellipsoids at 50% of probability, H atoms omitted for clarity. 48
Figure S44. Packing diagram of **H_2L2** viewed along *a* axis. Non-H ellipsoids at 50% of probability, H omitted for clarity. Molecules aligned into a row are equally coloured. 49
Figure S45. Packing diagram of **H_2L2** viewed along *b* axis. Non-H ellipsoids at 50% of probability, H omitted for clarity. Molecules aligned into a row are equally coloured. 50
Figure S46. ORTEP diagram of asymmetric unit of **Re2** crystal. Ellipsoids at 30% of probability, H atoms omitted for clarity. Left, the C ("clockwise"), right, the A ("anticlockwise") enantiomers. 51
Figure S47. HPLC and LC-MS analyses of the SPE-purified carrier added preparation of $^{99g/99m}$Tc1. (A) radio/UV-RP-HPLC chromatogram. (B) TIC/UV-RP-HPLC chromatogram (mass spectrometer in positive mode). (C) ESI(+)-MS spectrum of the peak at 12.09 min in the TIC chromatogram. (D) MS^2 spectrum of the peak at m/z 599. 52

Figure S48. HPLC and LC-MS analyses of the SPE-purified carrier added preparation of $^{99g/99m}$Tc2. (A) radio/UV-RP-HPLC chromatogram. (B) TIC/UV-RP-HPLC chromatogram (mass spectrometer in positive mode). (C) ESI(+)-MS spectrum of the peak at 21.35 min in the TIC chromatogram. (D) MS^2 spectrum of the peak at m/z 627. 53

Figure S49. HPLC and LC-MS analyses of the SPE-purified carrier added preparation of $^{99g/99m}$Tc3. (A) radio/UV-RP-HPLC chromatogram. (B) TIC/UV-RP-HPLC chromatogram (mass spectrometer in positive mode). (C) ESI(+)-MS spectrum of the peak at 15.95 min in the TIC chromatogram. (D) MS^2 spectrum of the peak at m/z 530. 54

Figure S50. Stability of ^{99m}Tc1-4 in: phosphate buffer saline (PBS); cysteine (Cys), 1 mM; glutathione (GSH), 1 mM; human serum, type AB (HS). 55

TABLES 56

Table S1. Principal crystallographic data for **H_2L2**, **Re1** and **Re2**. 57

Table S2. Bond lengths in asymmetric unit of **H_2L2**. 58

Table S3. Selected bond lengths in asymmetric unit of **Re1** 59

Table S4. Selected bond lengths in asymmetric unit of **Re2** 60

Table S5. Bond angles in asymmetric unit of **Re1**. 61

Table S6. Bond angles in asymmetric unit of **Re2**. 62

OTHER INFORMATION 64

Attempts of synthesis of [ReN(L1)PCN] 65

FIGURES

Figure S1. ESI(+)-MS analysis of **Re0**. Full from m/z 250 to m/z 1000.
*See Figure S5. **See Figure S6.

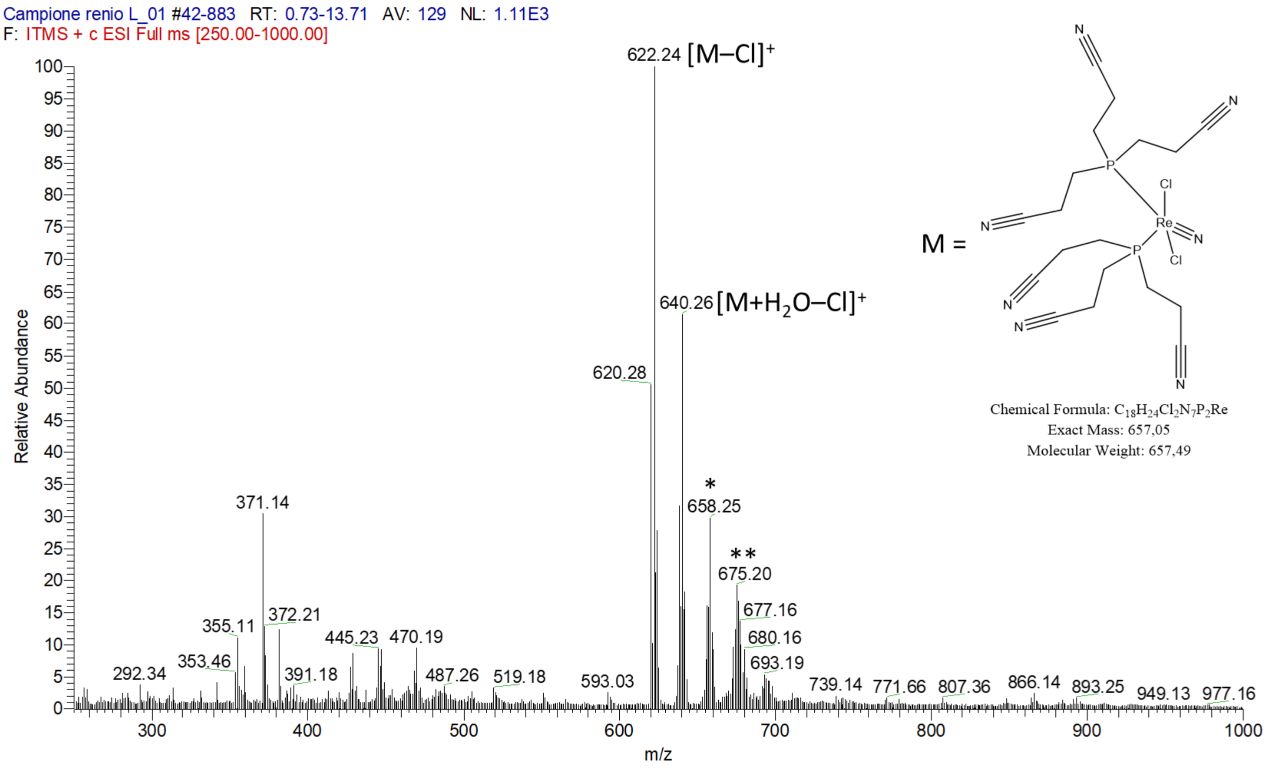

Figure S2. ESI(+)-MS analysis of **Re0**. Up: zoom scan, profile mode, from m/z 612 to m/z 652. Bottom: zoom scan, profile mode, from m/z 648 to m/z 688.
*See Figure S5. **See Figure S6.

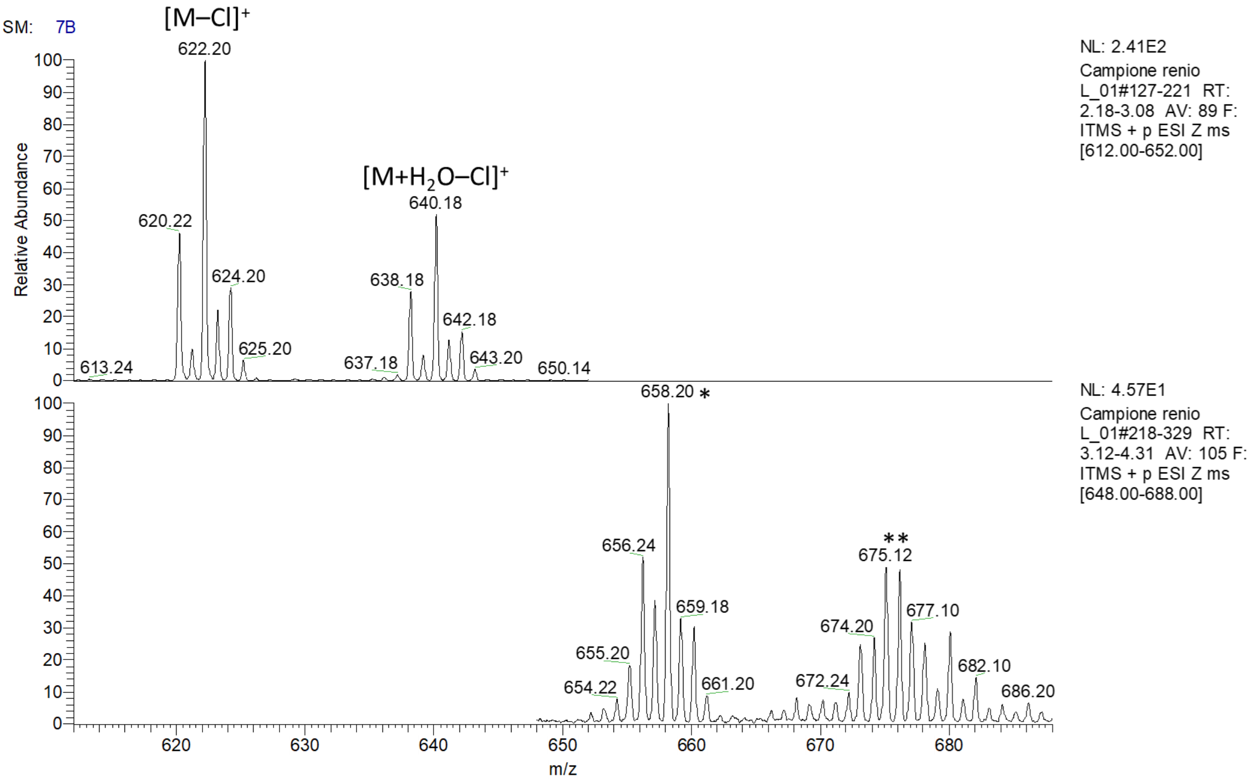

Figure S3. ESI(+)-MS analysis of **Re0**. Up: zoom scan, profile mode, from m/z 612 to m/z 652. Bottom: simulation for $C_{18}H_{24}ClN_7P_2Re^+$, $[M–Cl]^+$ (M = $C_{18}H_{24}Cl_2N_7P_2Re$).

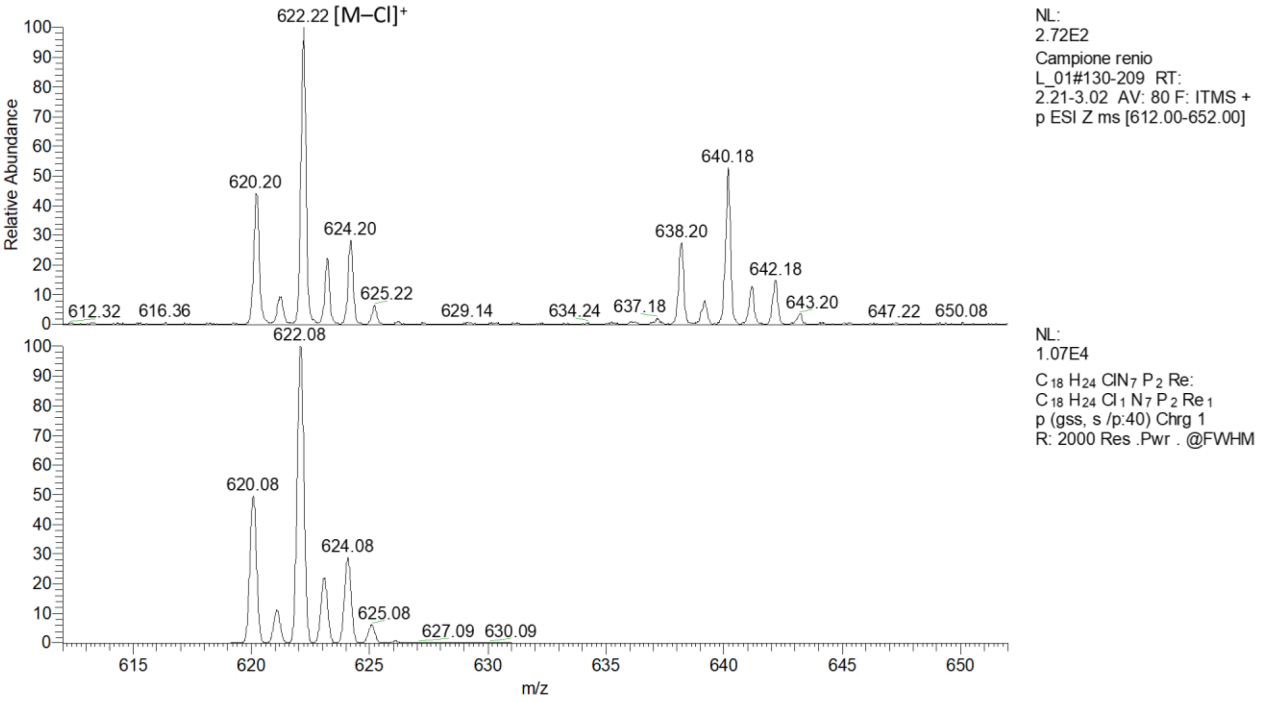

Figure S4. ESI(+)-MS analysis of **Re0**. Up: zoom scan, profile mode, from m/z 612 to m/z 652. Bottom: simulation for $C_{18}H_{26}ClN_7OP_2Re^+$, $[M+H_2O–Cl]^+$ (M = $C_{18}H_{24}Cl_2N_7P_2Re$).

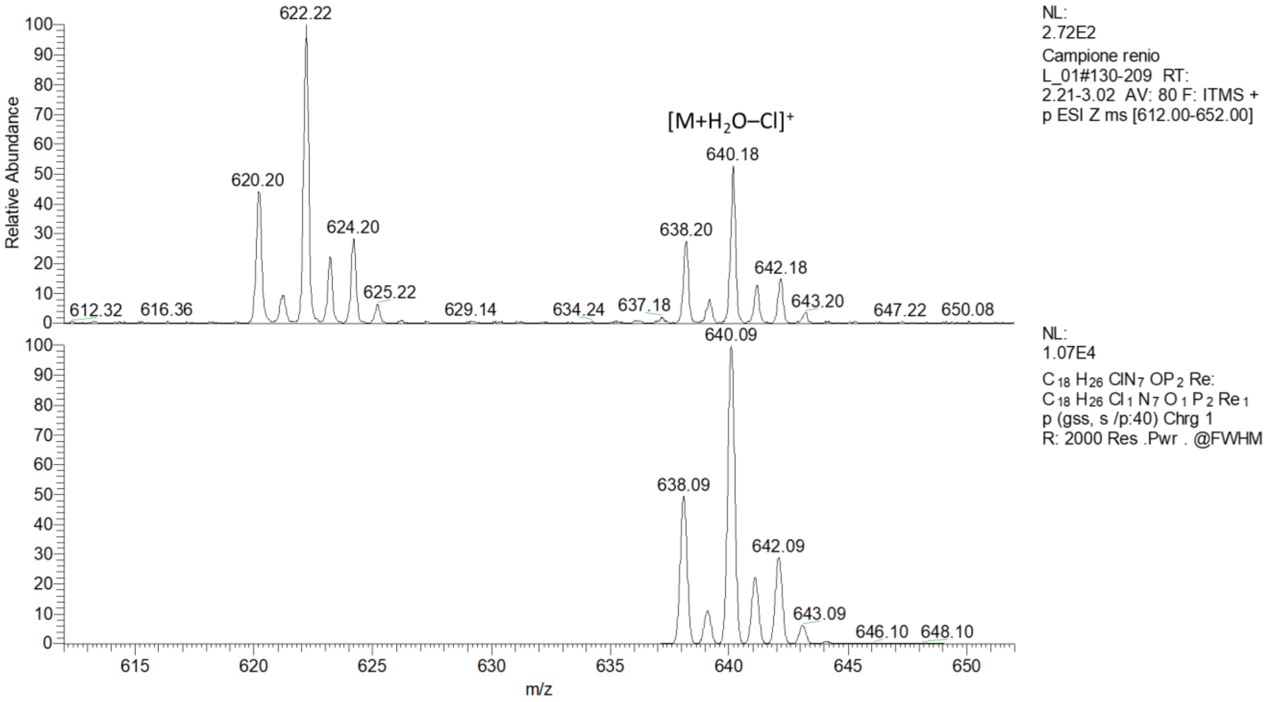

Figure S5. ESI(+)-MS analysis of **Re0**. Up: zoom scan, profile mode, from m/z 648 to m/z 688. Bottom: simulation for a mixture of $C_{18}H_{24}Cl_2N_7P_2Re^{+\cdot}$ ($[M]^{+\cdot}$, m/z 657) and $C_{18}H_{25}Cl_2N_7P_2Re^{+}$ ($[M+H]^{+}$, m/z 658), ratio 0.3:1.

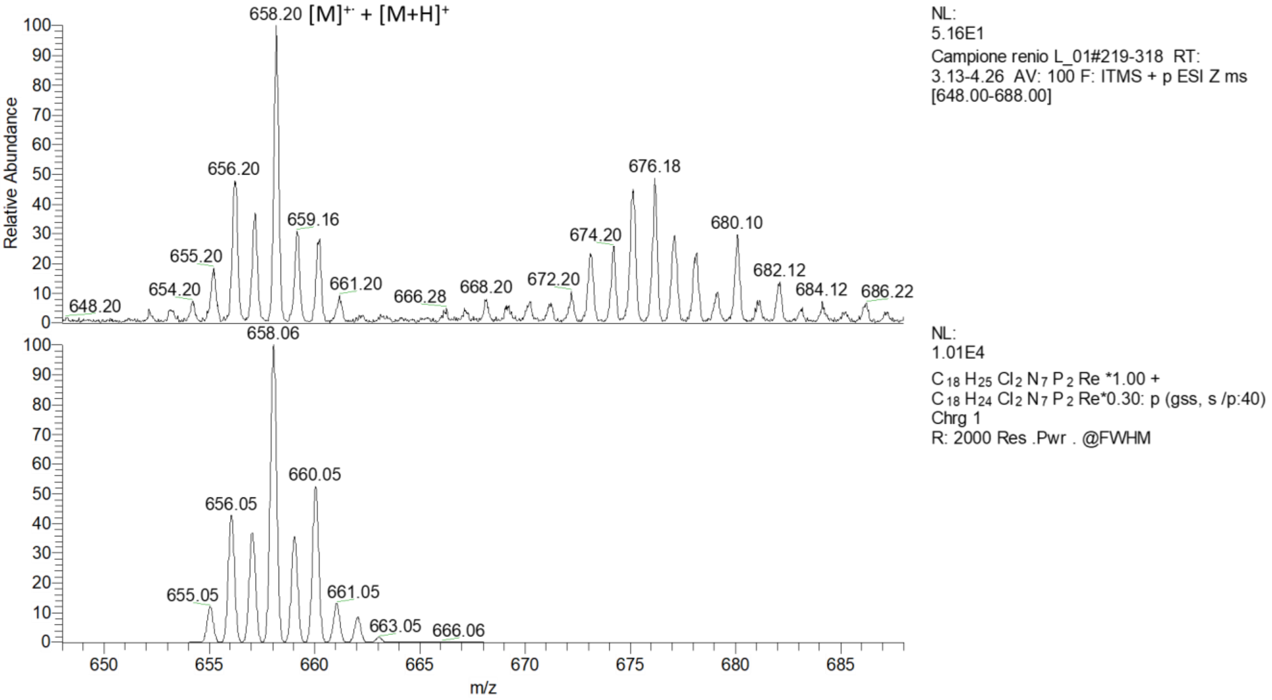

Figure S6. ESI(+)-MS analysis of **Re0**. Up: zoom scan, profile mode, from m/z 648 to m/z 688. Bottom: simulation for a mixture of $C_{18}H_{26}Cl_2N_7OP_2Re^{+\cdot}$ ($[M+H_2O]^{+\cdot}$) and $C_{18}H_{27}Cl_2N_7OP_2Re^{+}$ ($[M+H_2O+H]^{+}$), ratio 1:1.

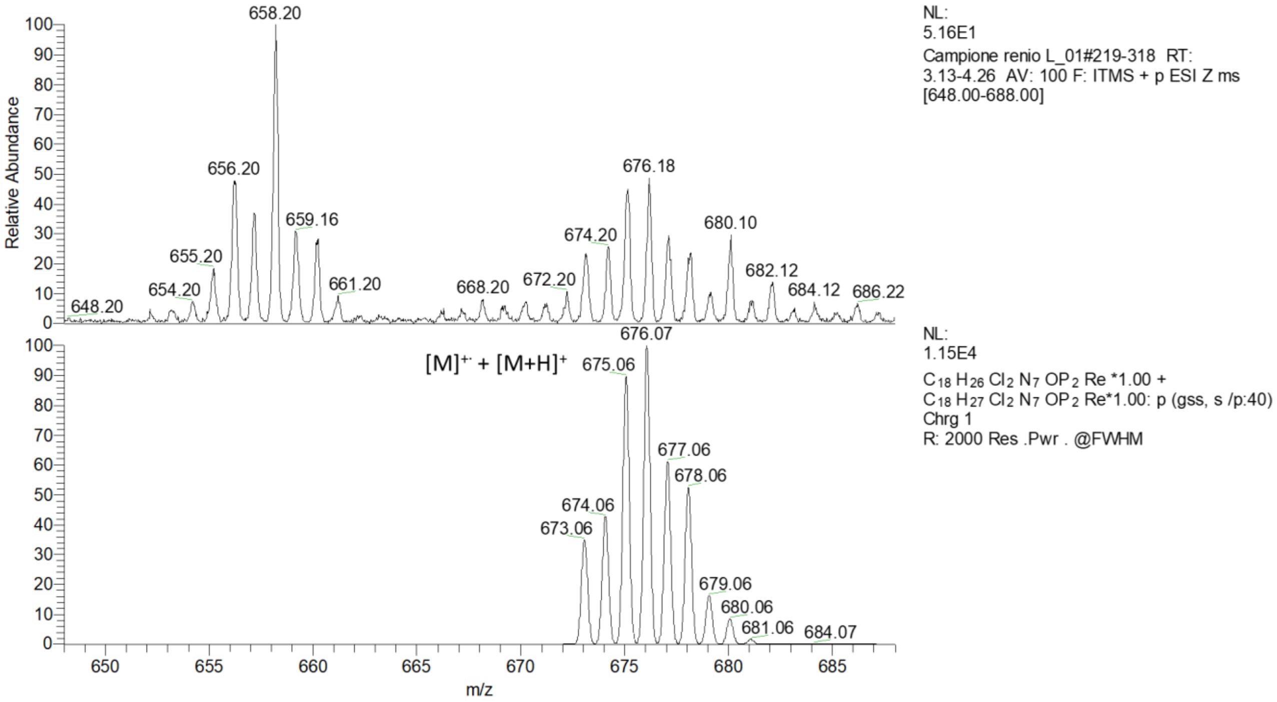

Figure S7. ESI(+)-MS analysis of **Re0**. ESI(+)-MS^2 (up) and ESI(+)-MS^3 (bottom), ion at m/z 658.

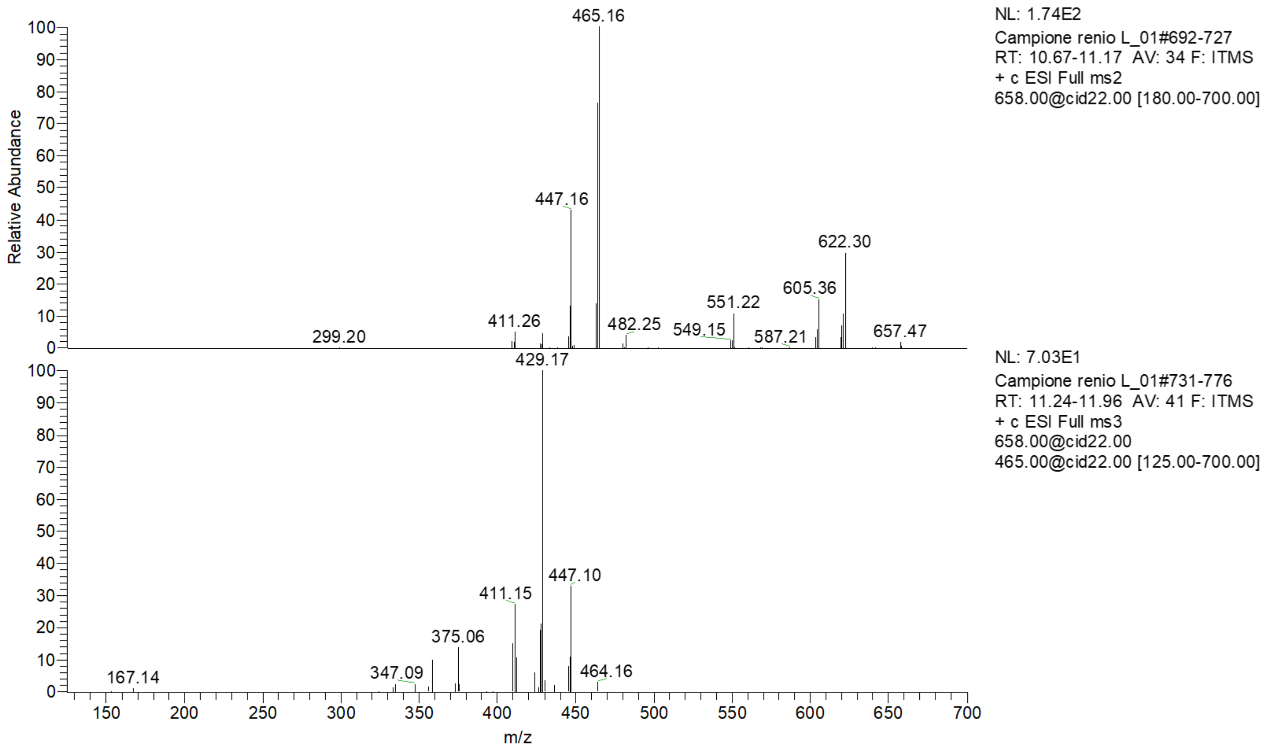

Figure S8. Hypothetic fragmentation pathway for the ion at m/z 658 according to the MS^n spectra in Figure S7.

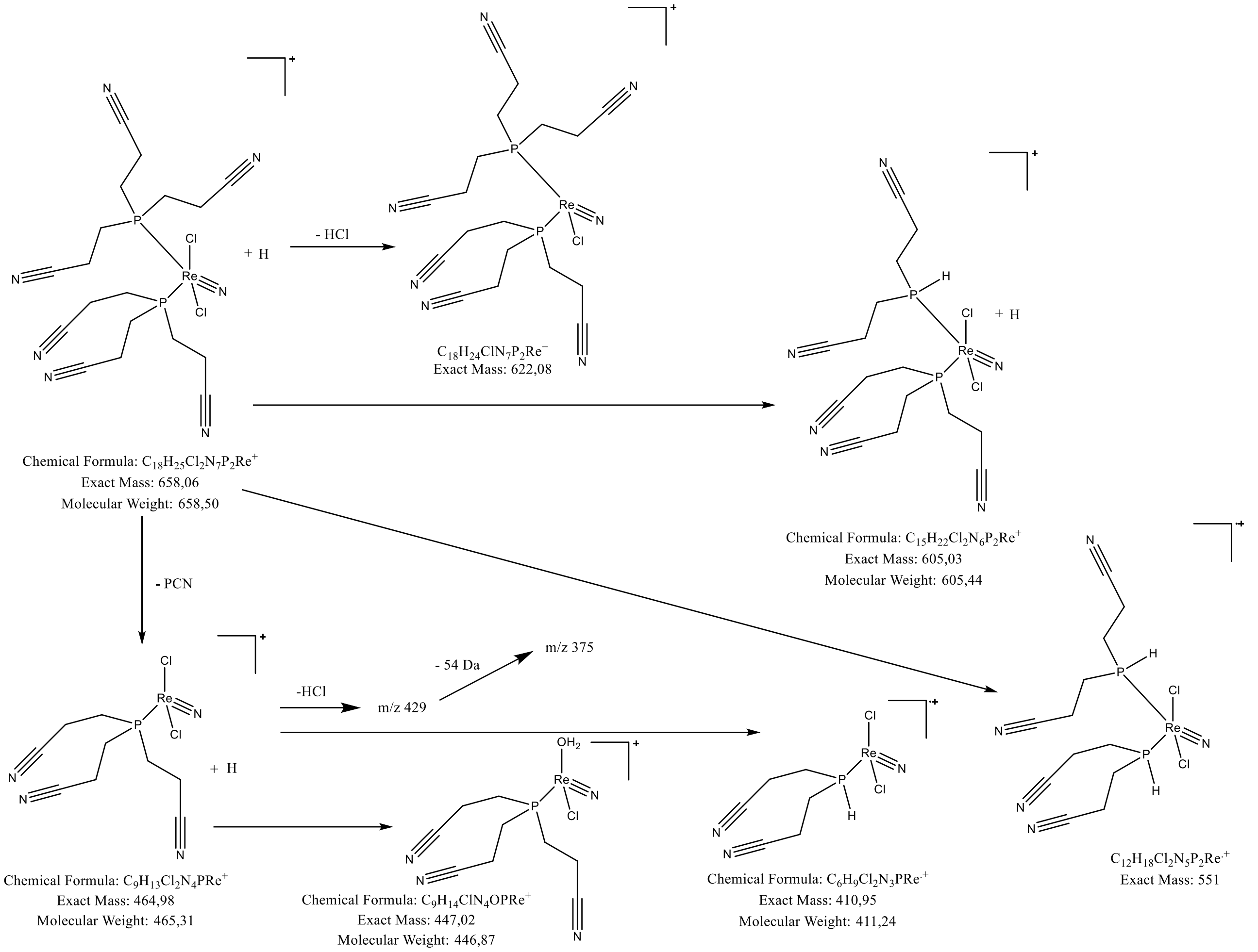

Figure S9. ESI(+)-MS analysis of **Re0**. ESI(+)-MS^2 (up) and ESI(+)-MS^3 (bottom), ion at m/z 640.

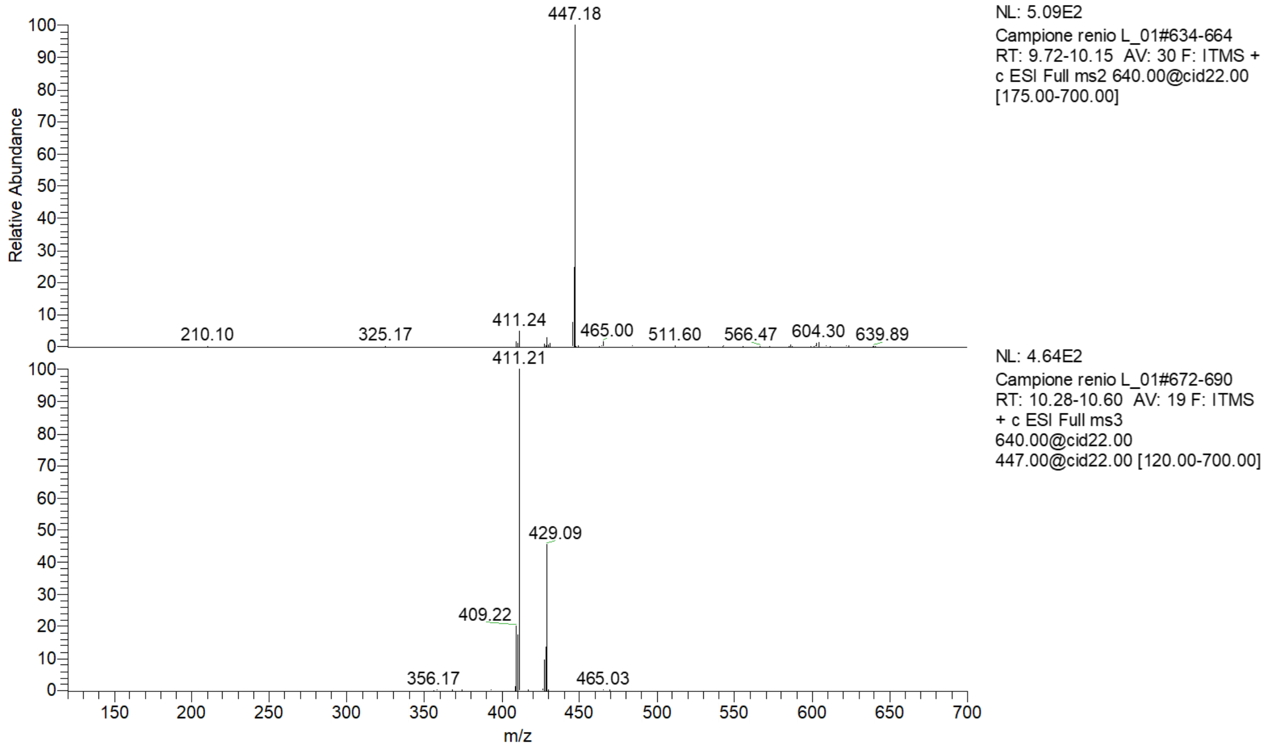

Figure S10. Hypothetic fragmentation pathway for the ion at m/z 640 according to the MS^n spectra in Figure S9.

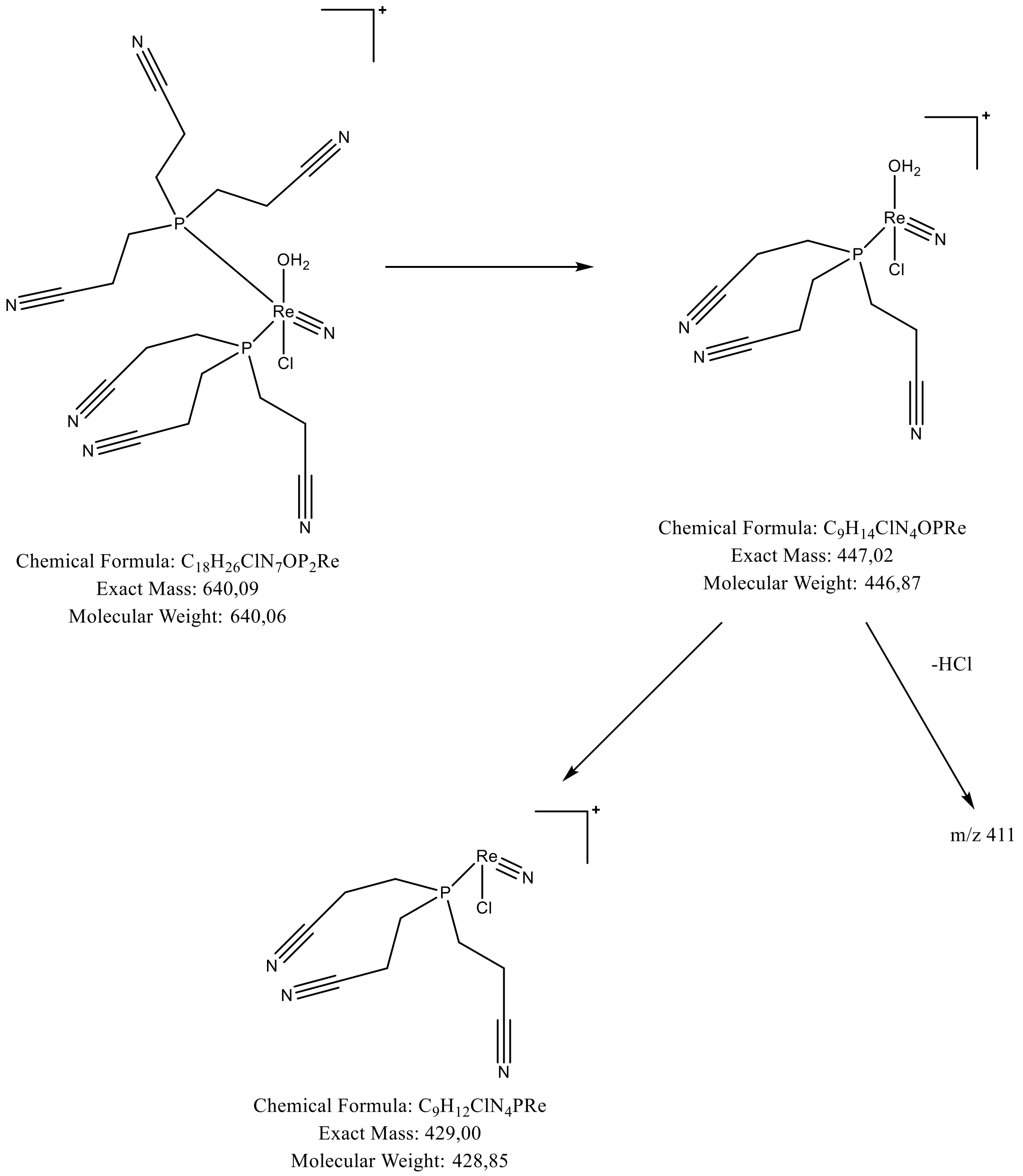

Figure S11. ESI(+)-MS analysis of **Re0**. ESI(+)-MS^2 (up), ESI(+)-MS^3 (middle) and ESI(+)-MS^4 (bottom), ion at m/z 622.

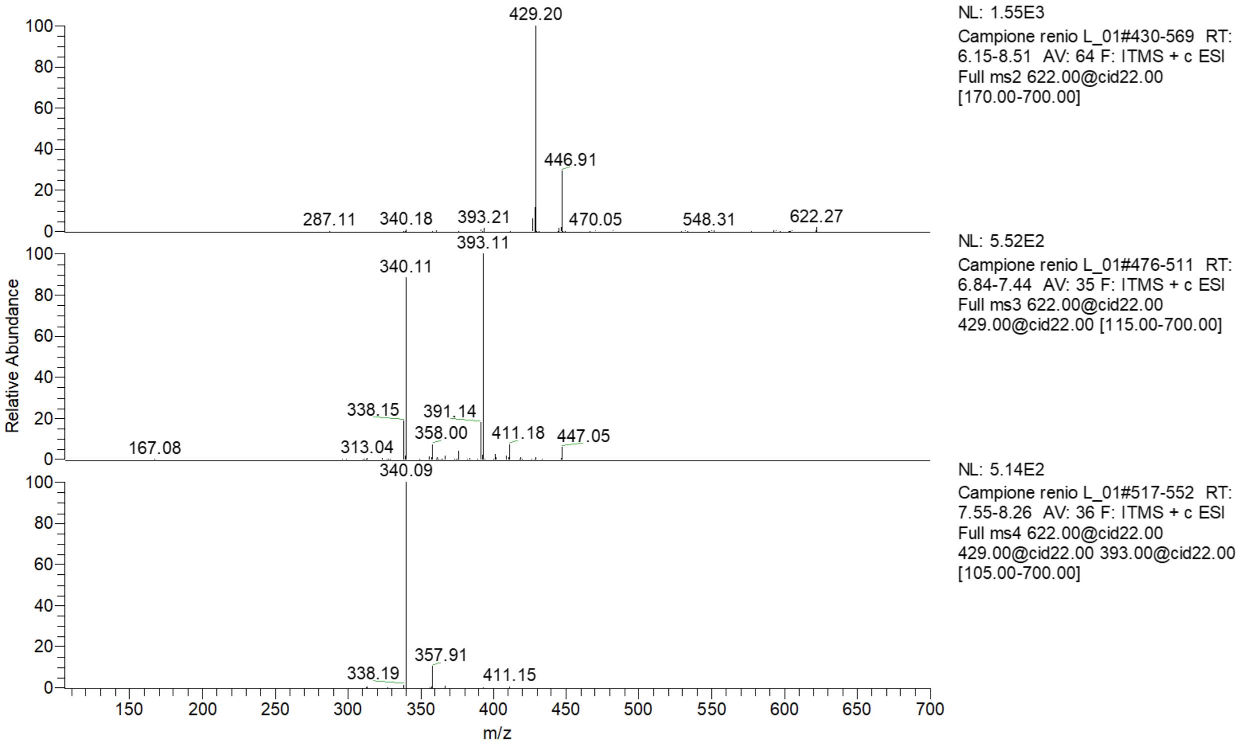

Figure S12. Hypothetic fragmentation pathway for the ion at m/z 622 according to the MS^n spectra in Figure S11.

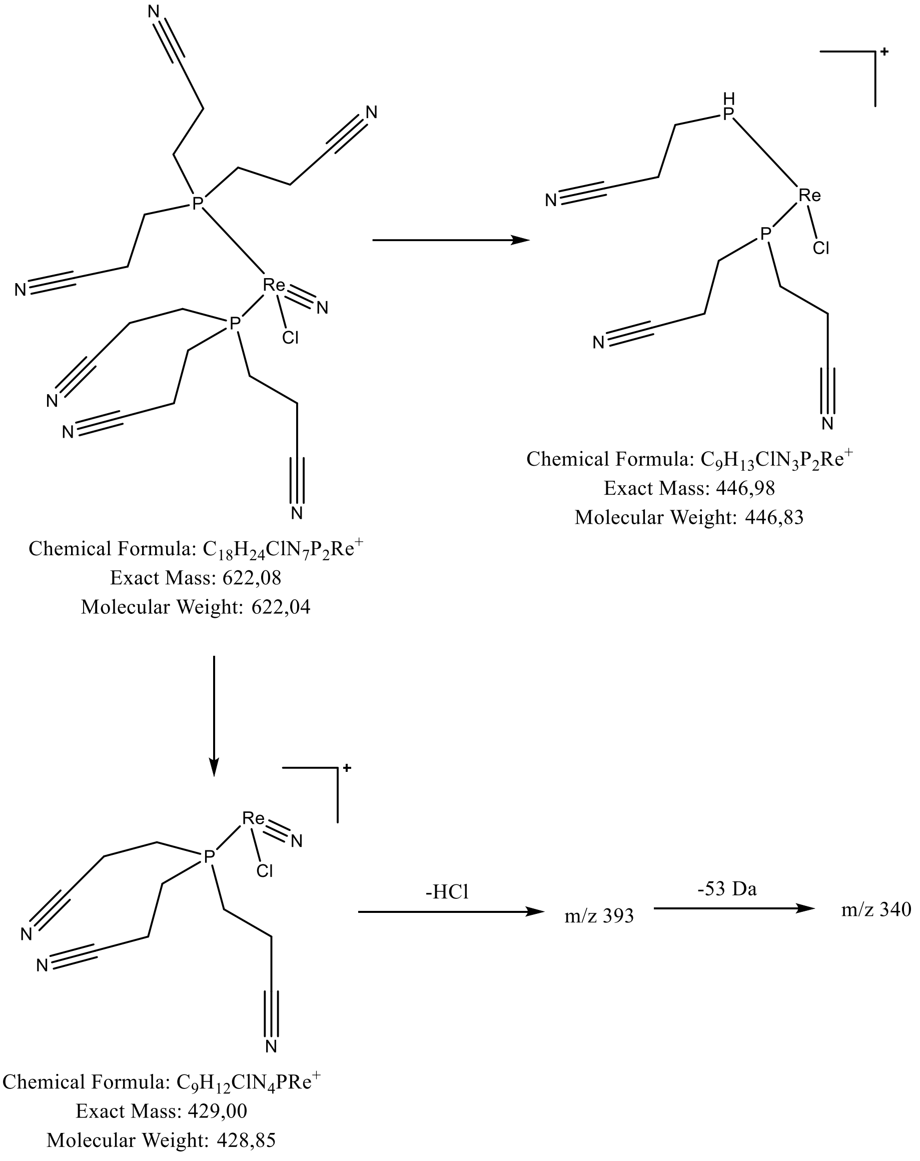

Figure S13. ESI(+)-MS analysis of **Re1**. Full from m/z 150 to m/z 2000.

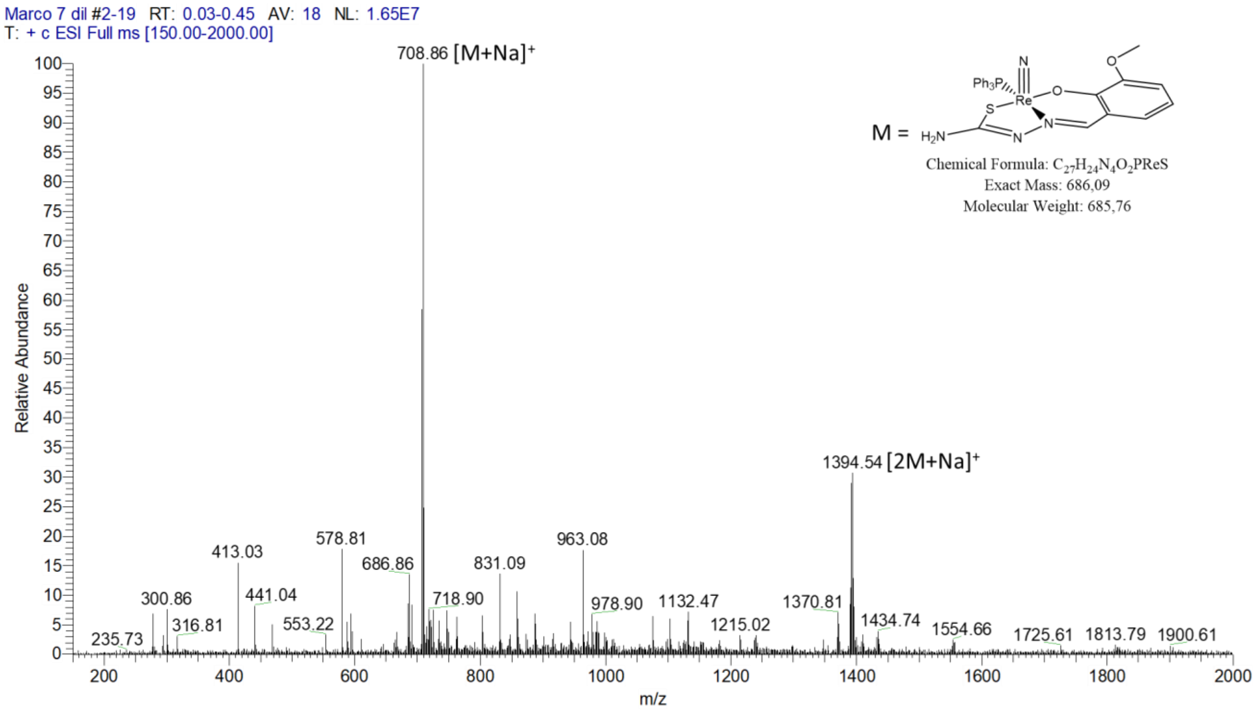

Figure S14. ESI(+)-MS analysis of **Re1**. Simulation for $C_{27}H_{24}N_4O_2PSRe+Na^+$, $[M+Na]^+$ (M = $C_{27}H_{24}N_4O_2PSRe$).

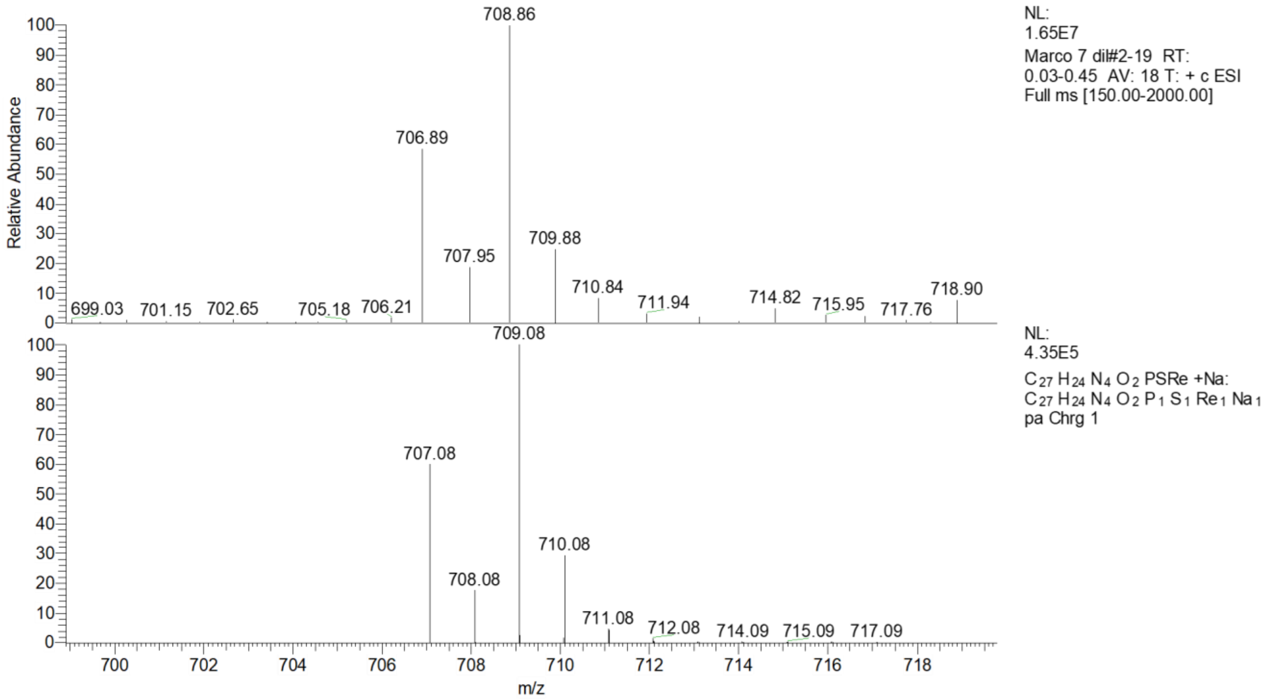

Figure S15. ESI(+)-MS analysis of **Re2**. Full from m/z 150 to m/z 2000.

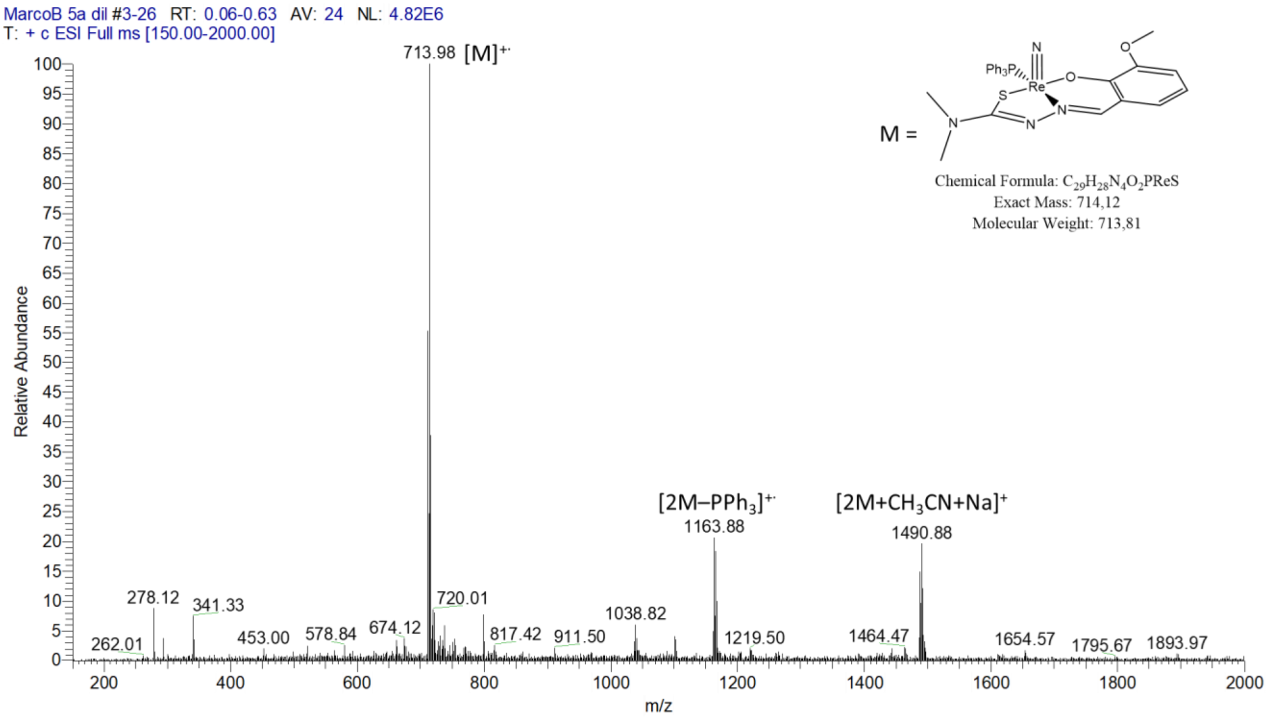

Figure S16. ESI(+)-MS analysis of **Re2**. Simulation for $C_{29}H_{28}N_4O_2PSRe^+$, $[M]^{+\cdot}$ (M = $C_{29}H_{28}N_4O_2PSRe$).

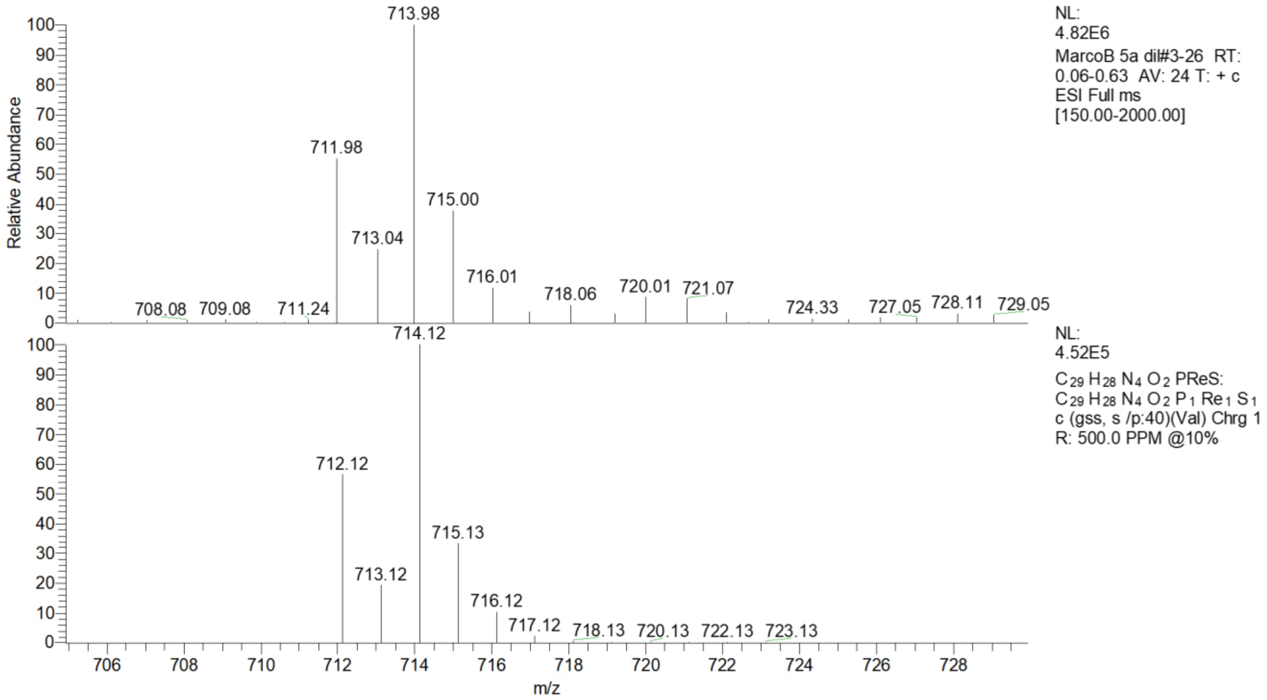

Figure S17. FT-IR spectrum (in KBr) of **Re0**.

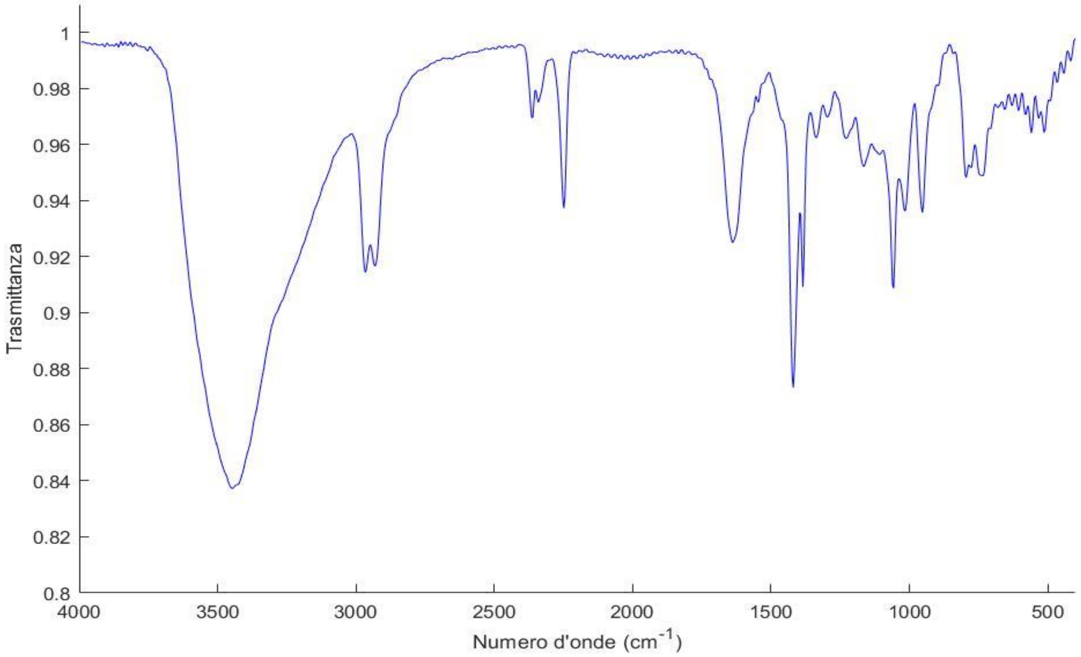

Figure S18. Blue: FT-IR spectrum (in KBr) of **Re0**. Black: FT-IR spectrum (in KBr) of **PCN**.

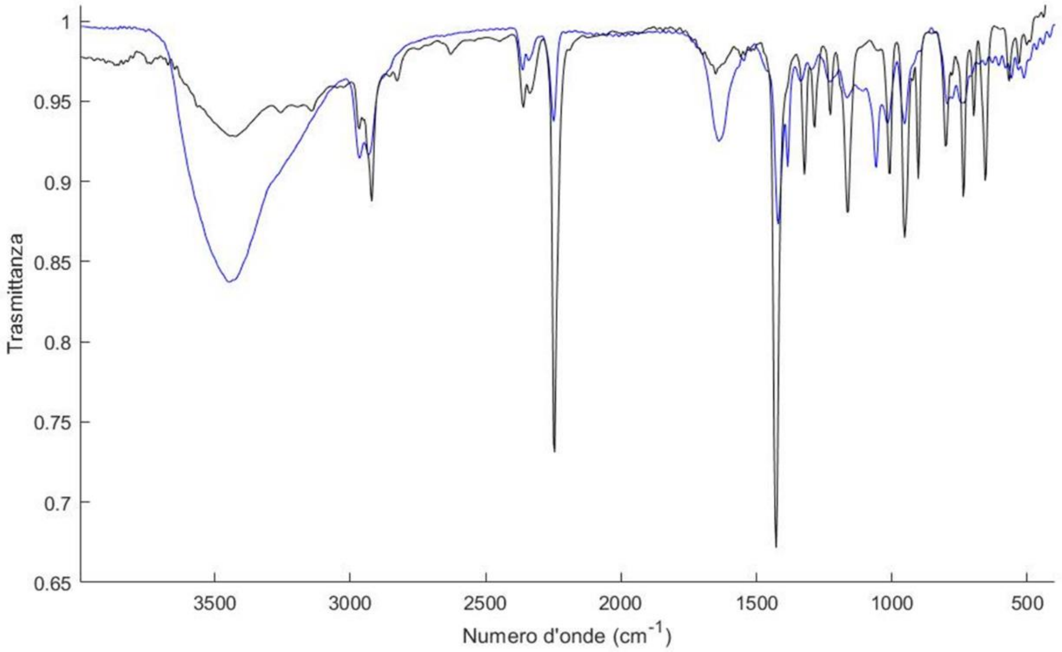

Figure S19. FT-IR spectrum (in KBr) of **Re1**.

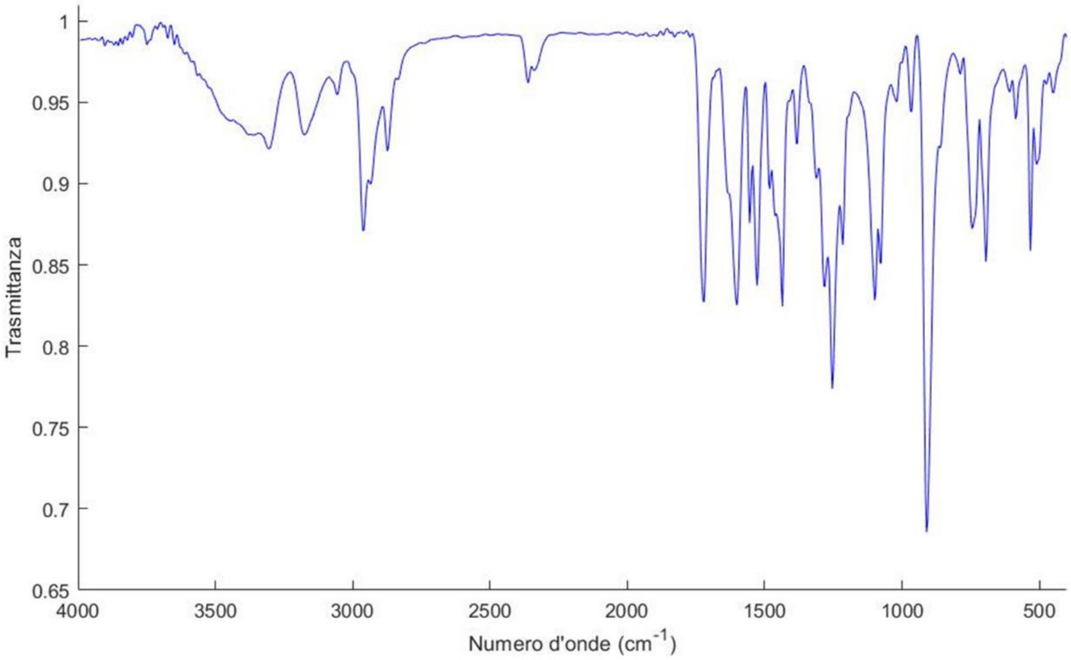

Figure S20. Blue: FT-IR spectrum (in KBr) of **Re1**. Red: FT-IR spectrum (in KBr) of **H_2L1**.

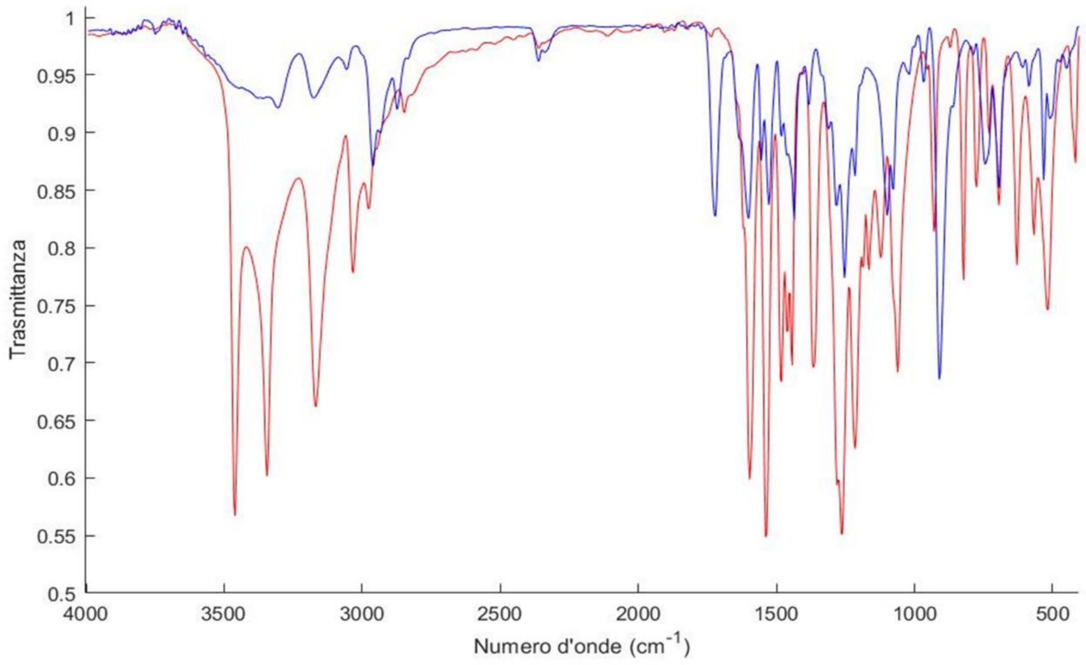

Figure S21. FT-IR spectrum (in KBr) of **Re2**.

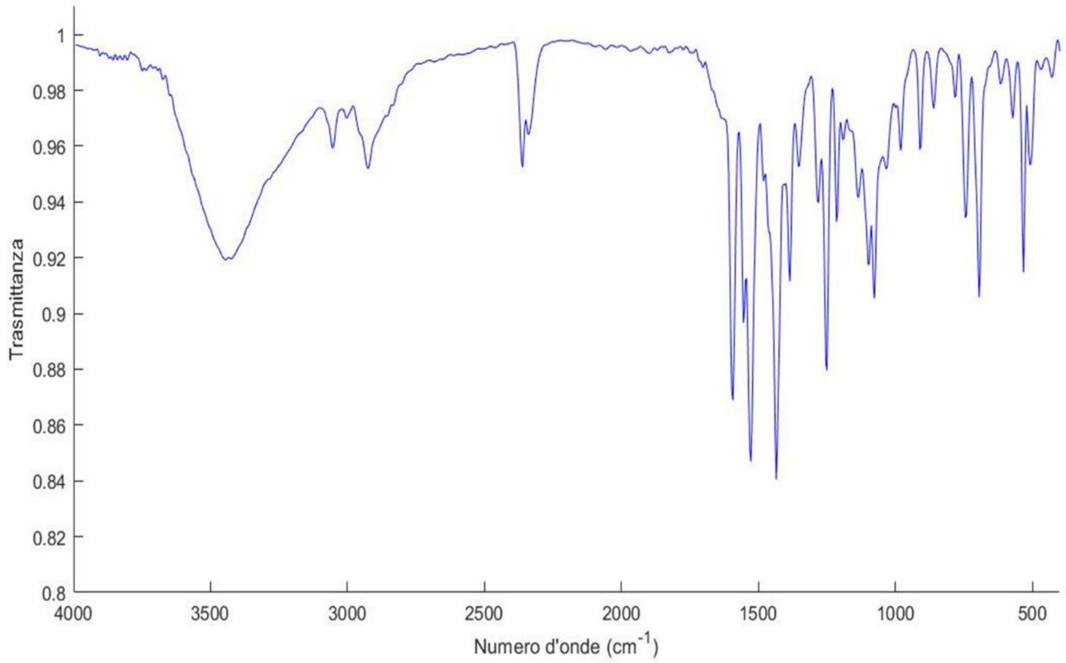

Figure S22. Blue: FT-IR spectrum (in KBr) of **Re2**. Red: FT-IR spectrum (in KBr) of **H_2L2**.

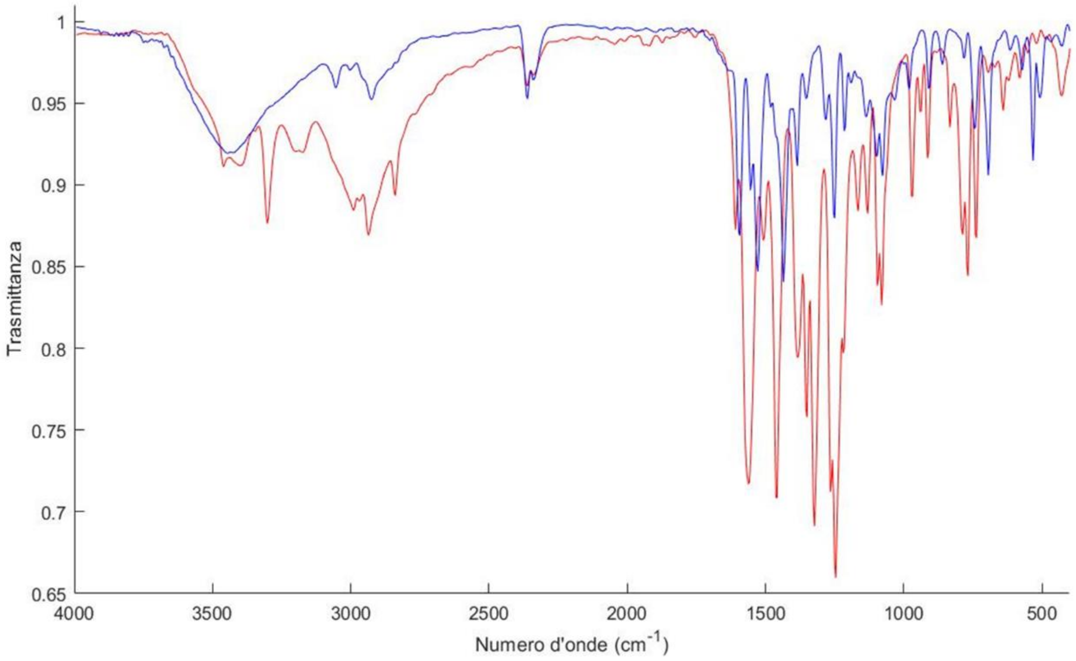

Figure S23. $^{31}P\{^{1}H\}$ NMR spectrum in CD_3CN of **Re0**.

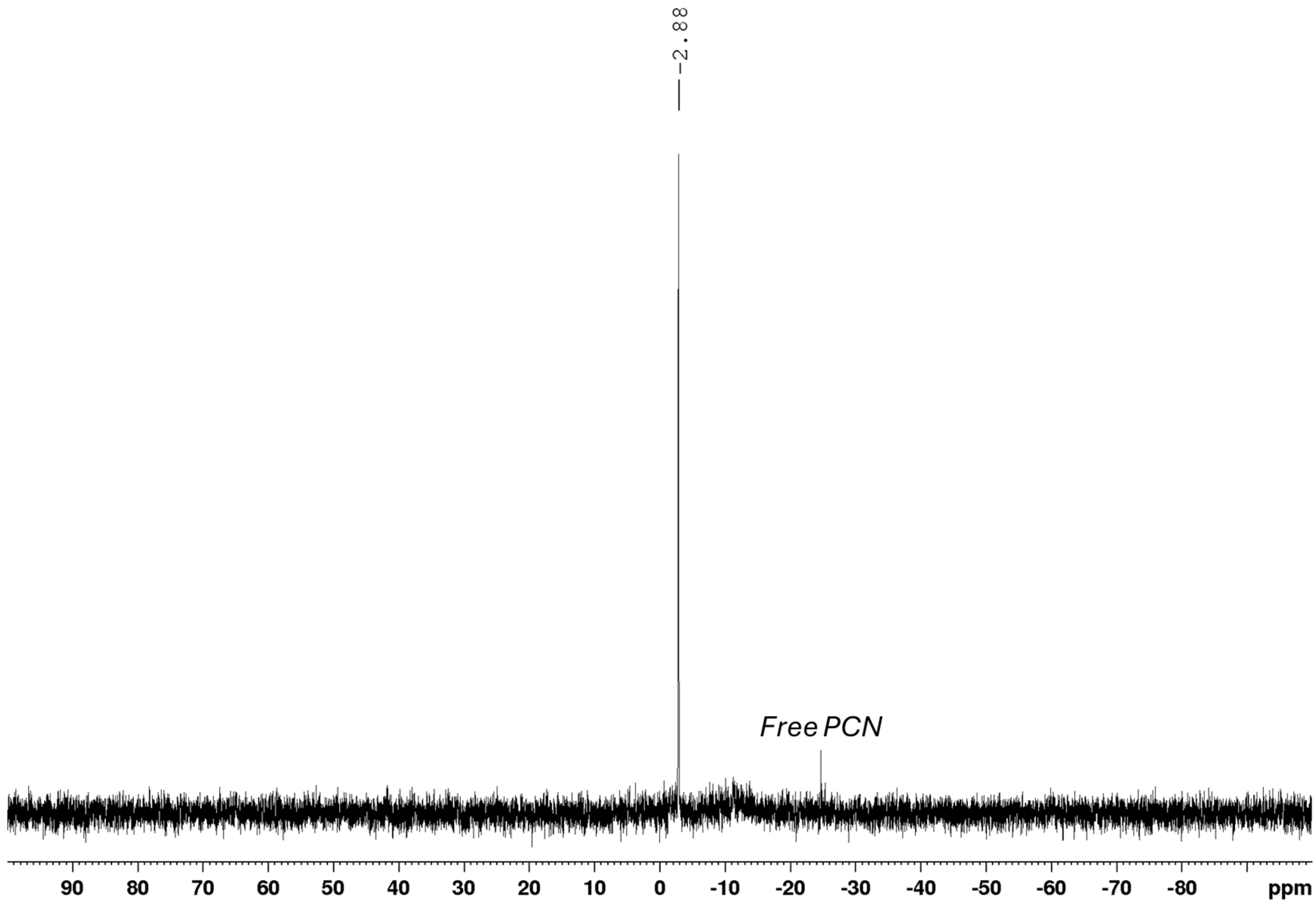

Figure S24. ^1H NMR spectrum in CD3CN of **Re0**.

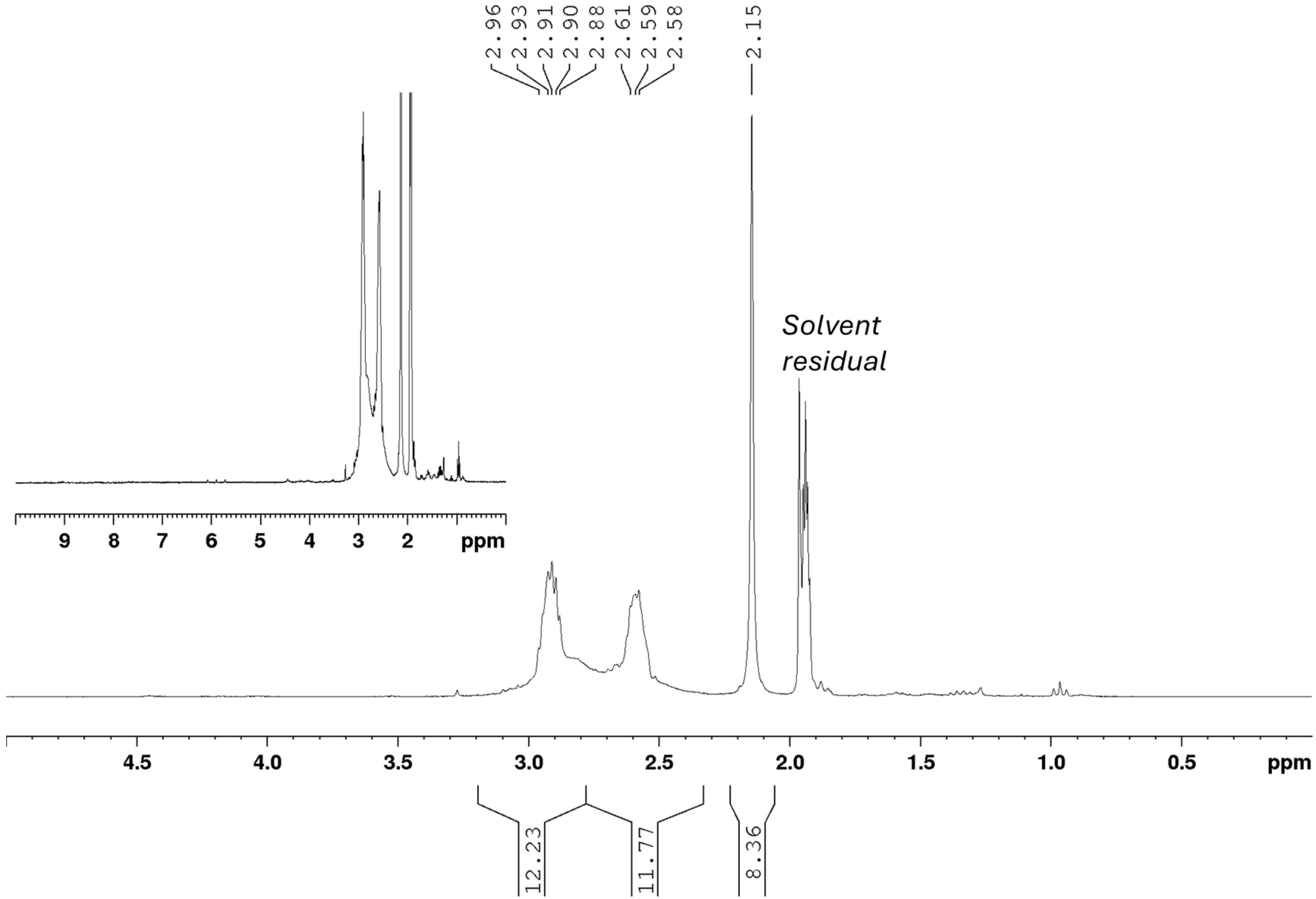

Figure S25. $^{13}C\{^1H\}$ NMR spectrum in CD_3CN of **Re0**.

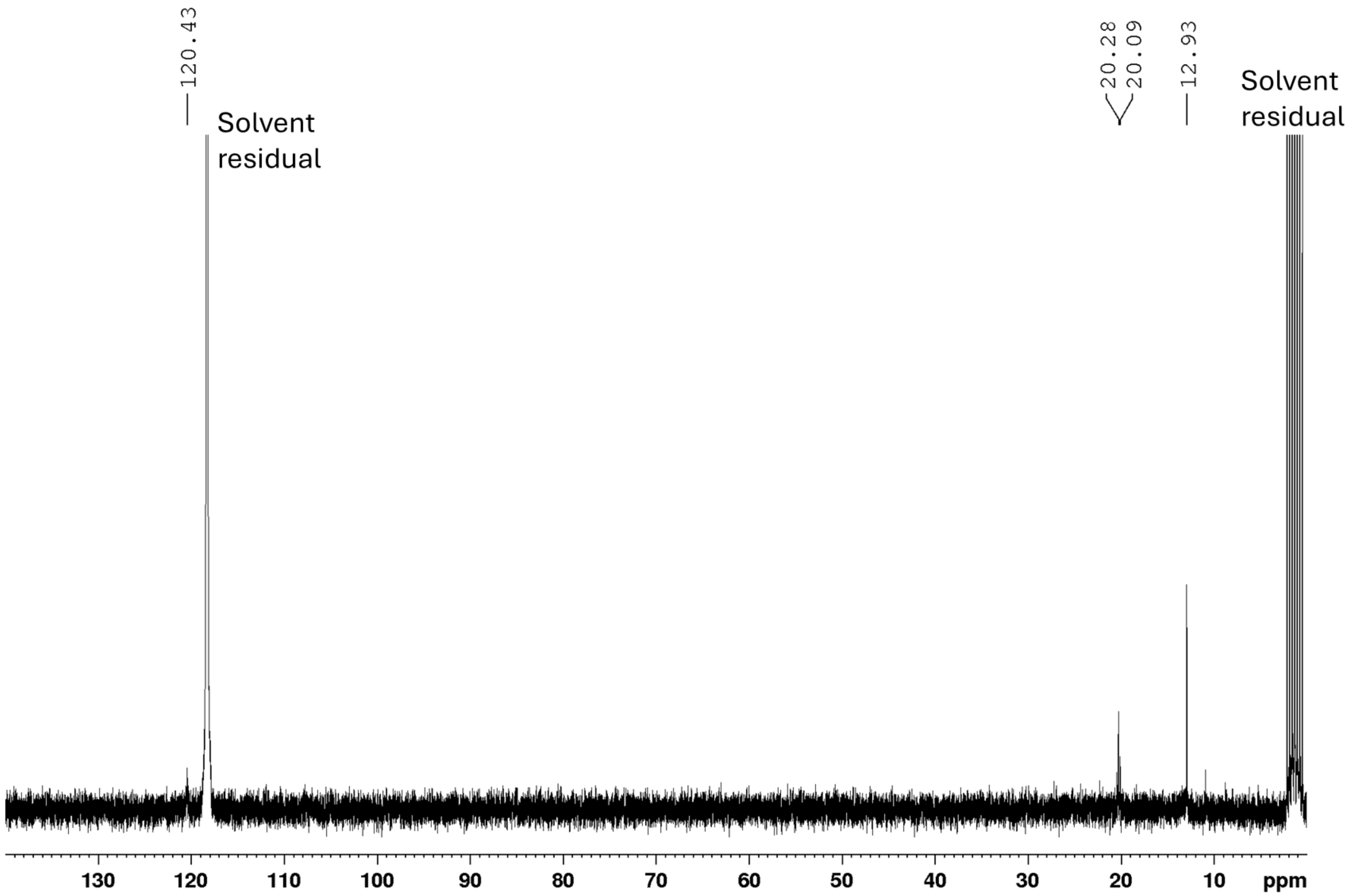

Figure S26. ^{1}H-^{13}C HMBC in CD_3CN of **Re0**.

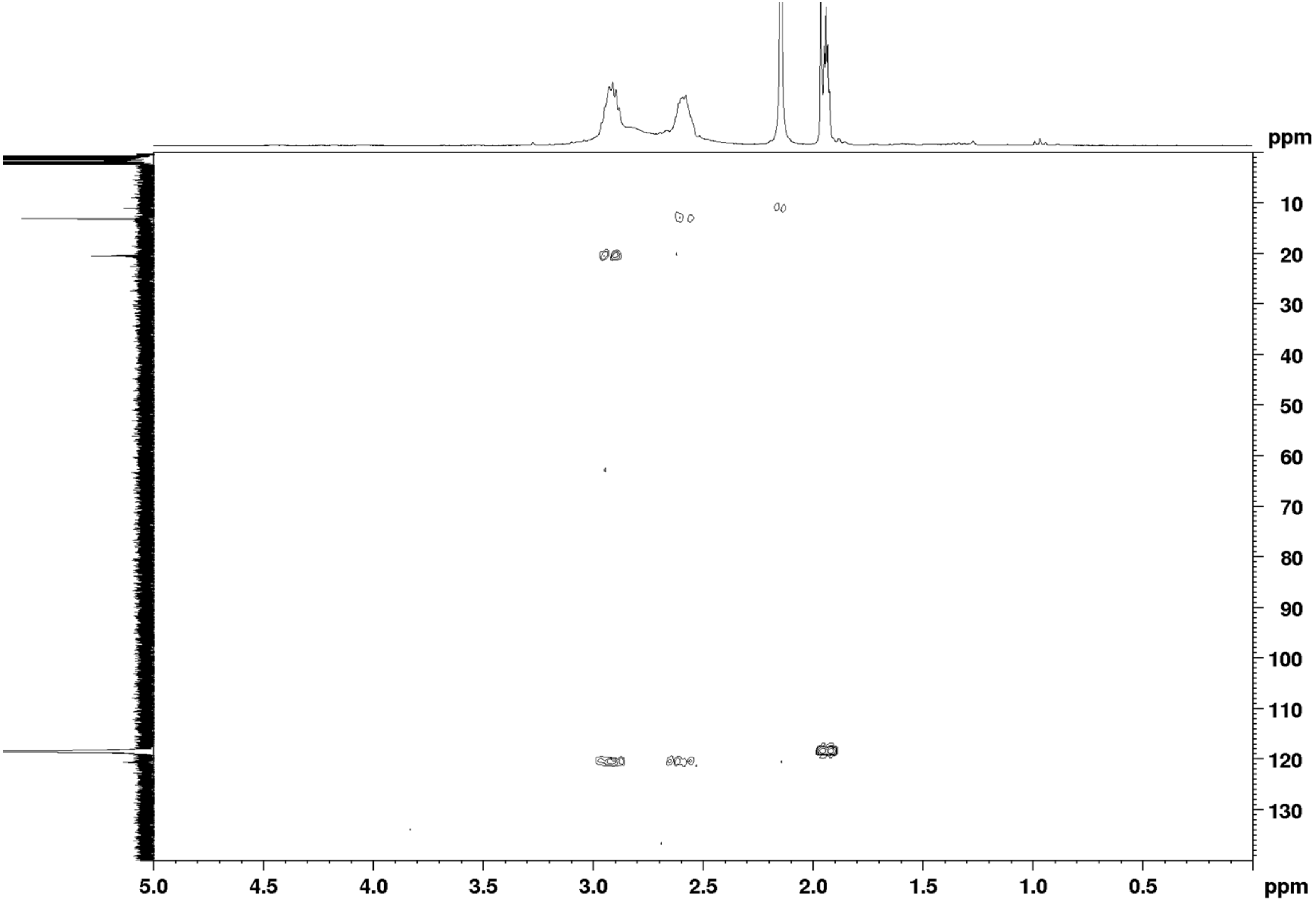

Figure S27. $^{31}P\{^{1}H\}$ NMR spectrum in CD_2Cl_2 of **Re1**.

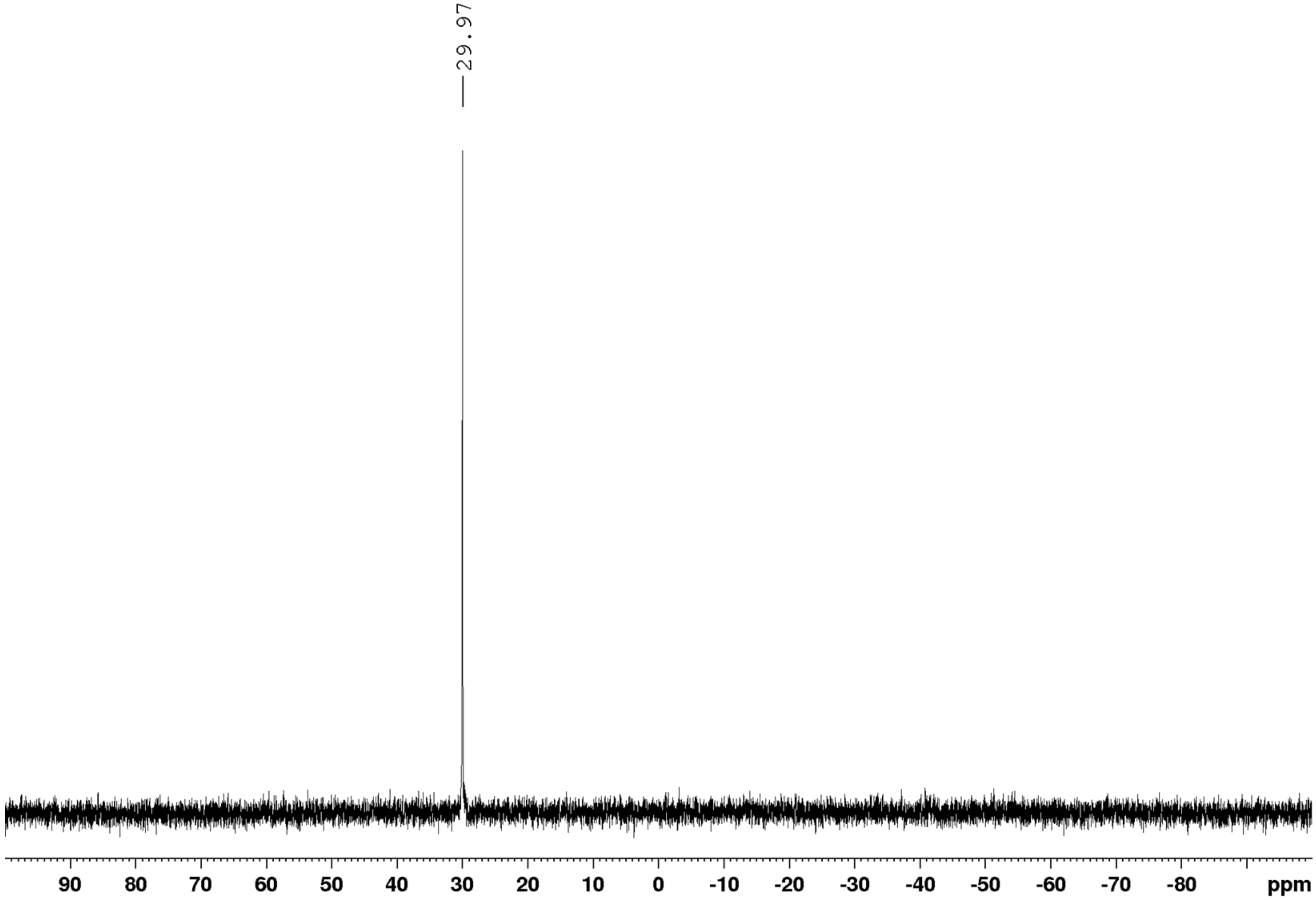

Figure S28. ^{1}H NMR spectrum in CD_2Cl_2 of **Re1**.

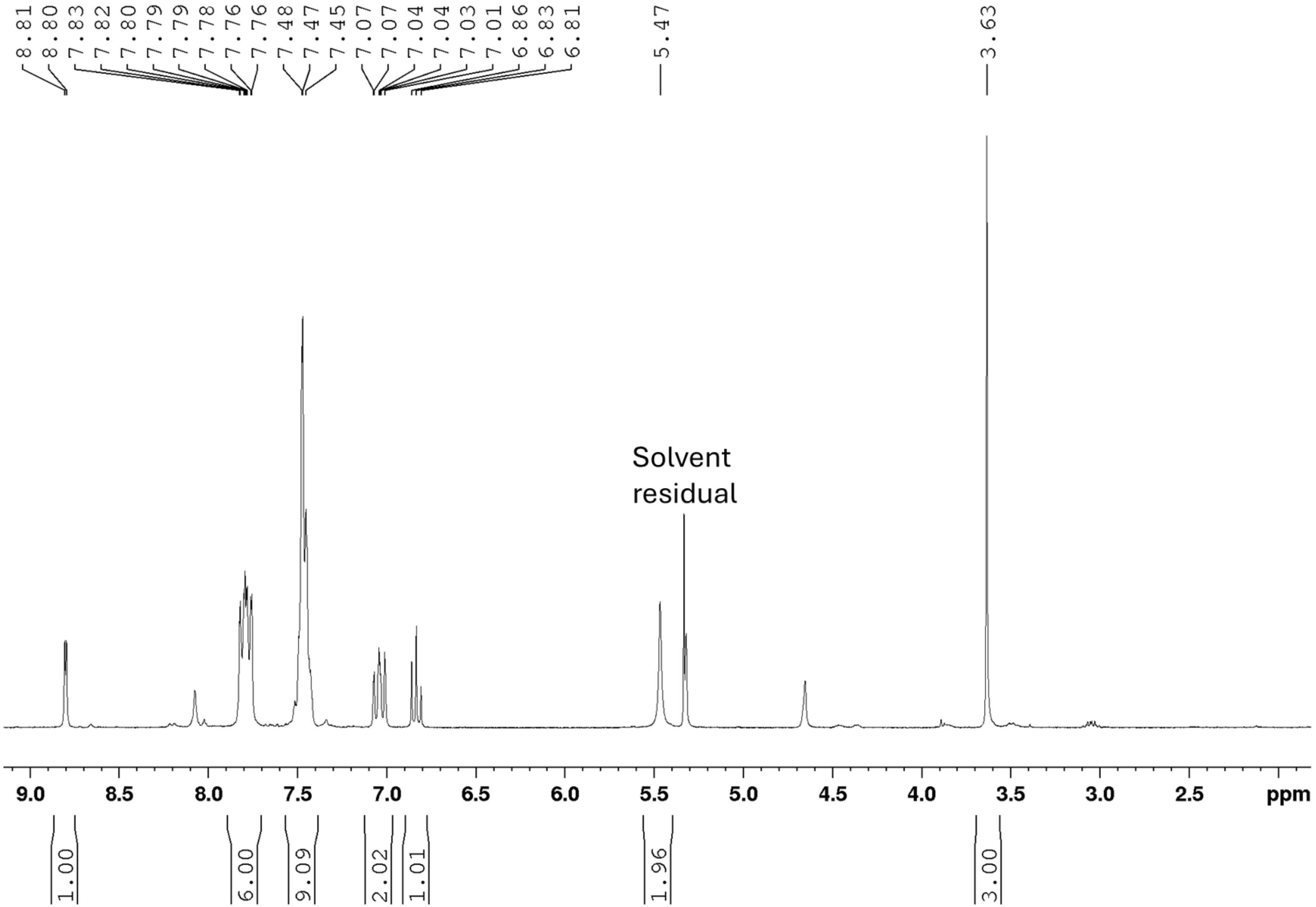

Figure S29. $^{13}C\{^1H\}$ NMR spectrum in CD_2Cl_2 of **Re1**.

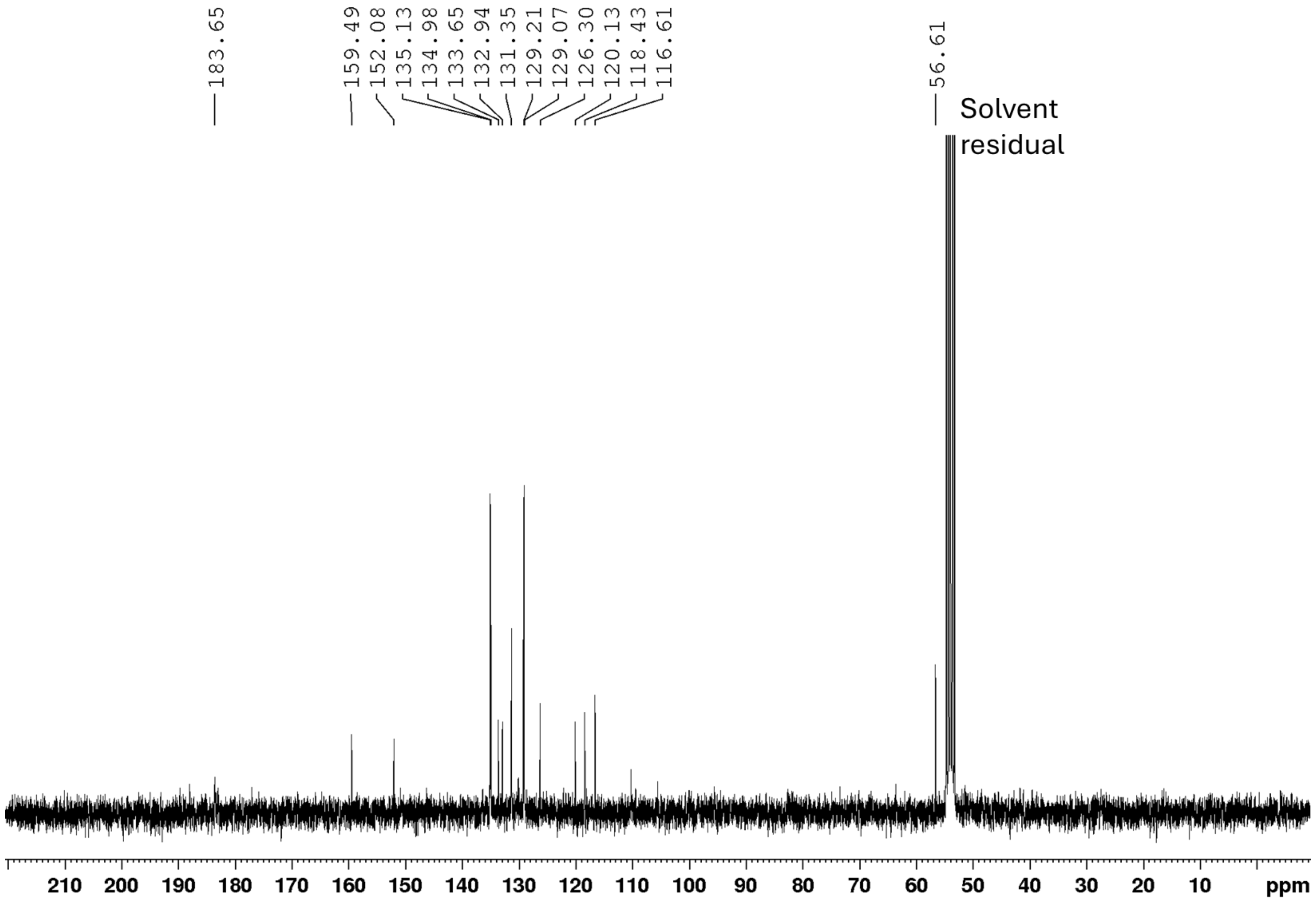

Figure S30. ^{1}H-^{13}C HMQC in CD_2Cl_2 of **Re1**.

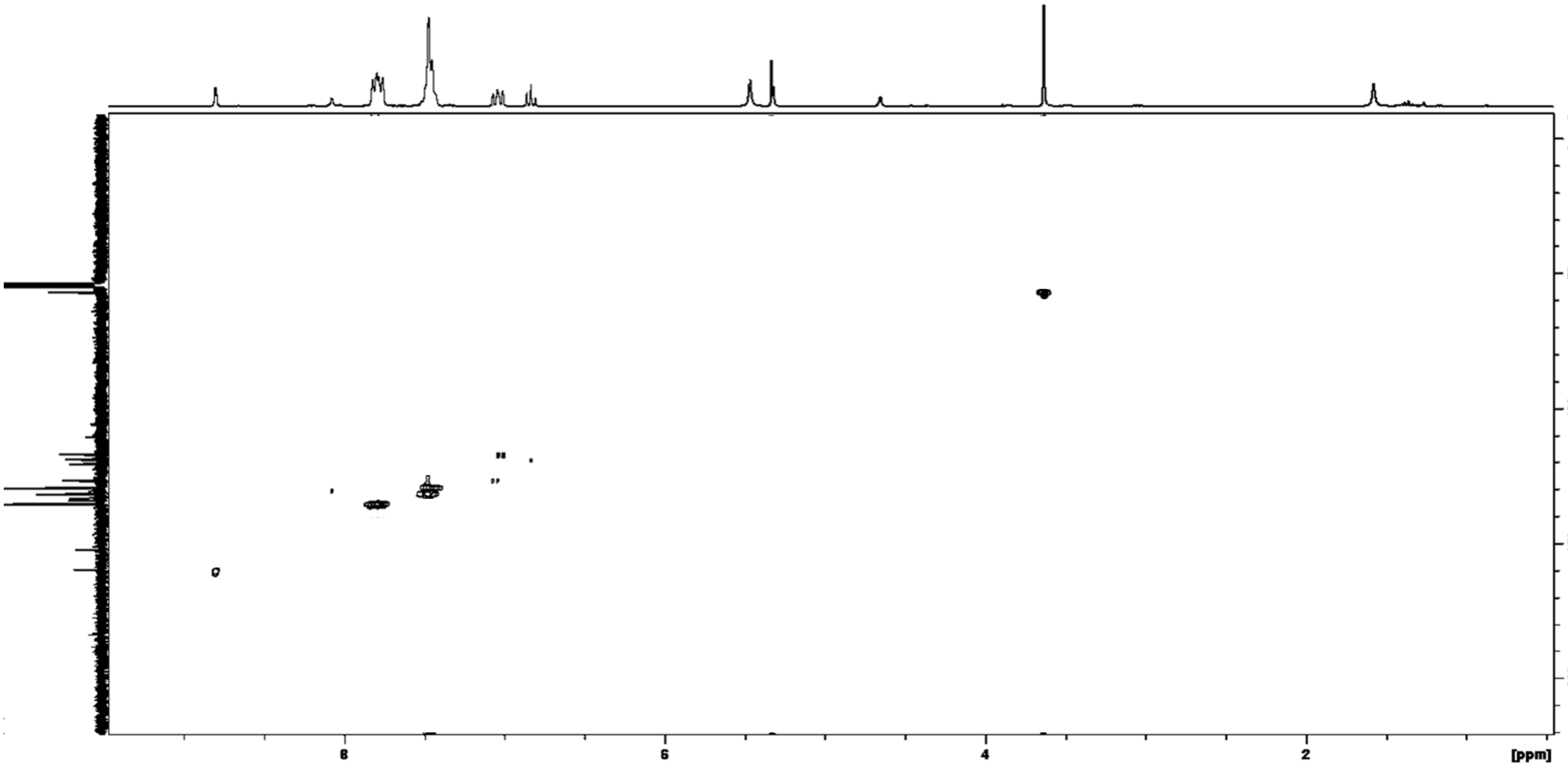

Figure S31. ^{1}H-^{13}C HMBC in CD_2Cl_2 of **Re1**.

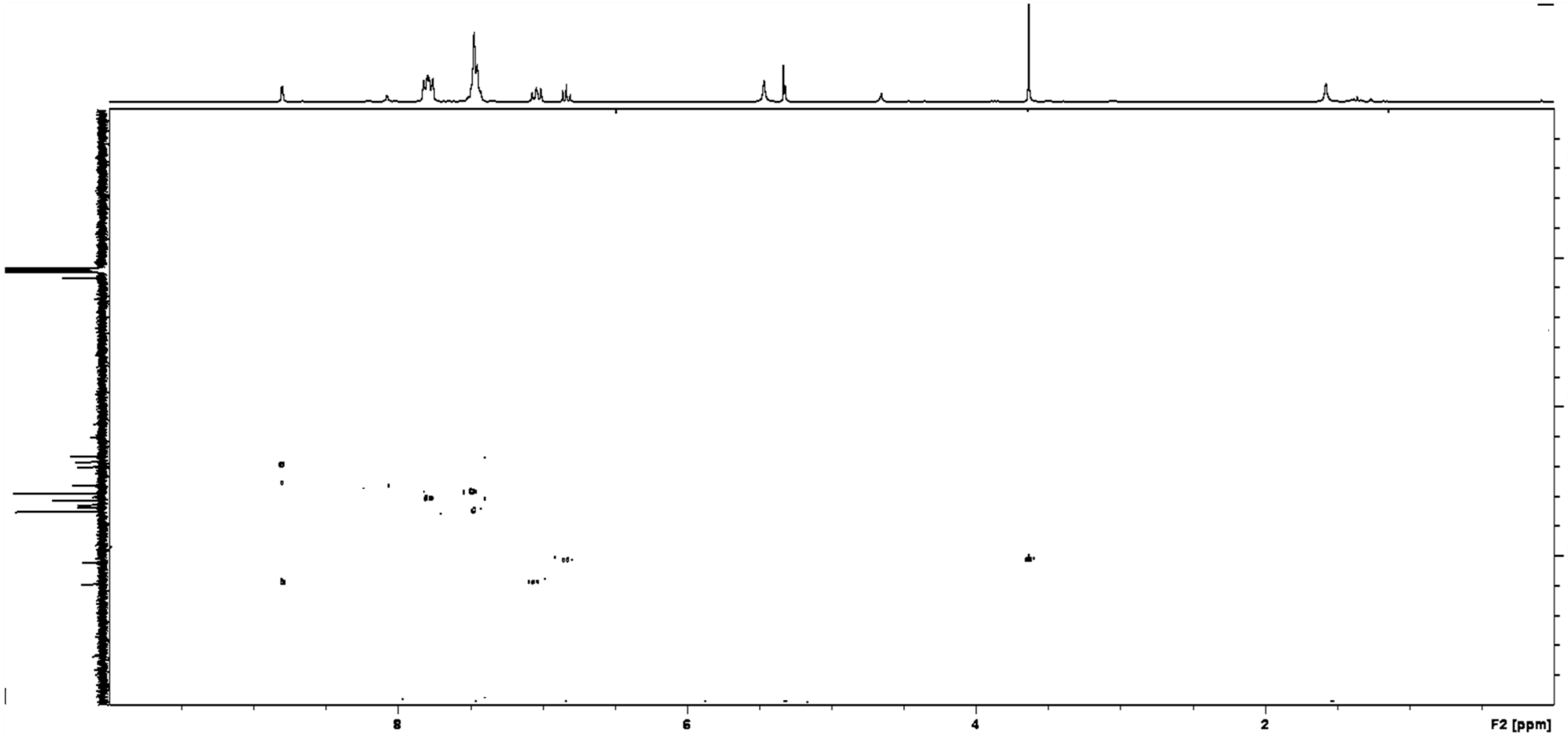

Figure S32. ^{1}H-^{31}P HMBC in CD_2Cl_2 of **Re1**.

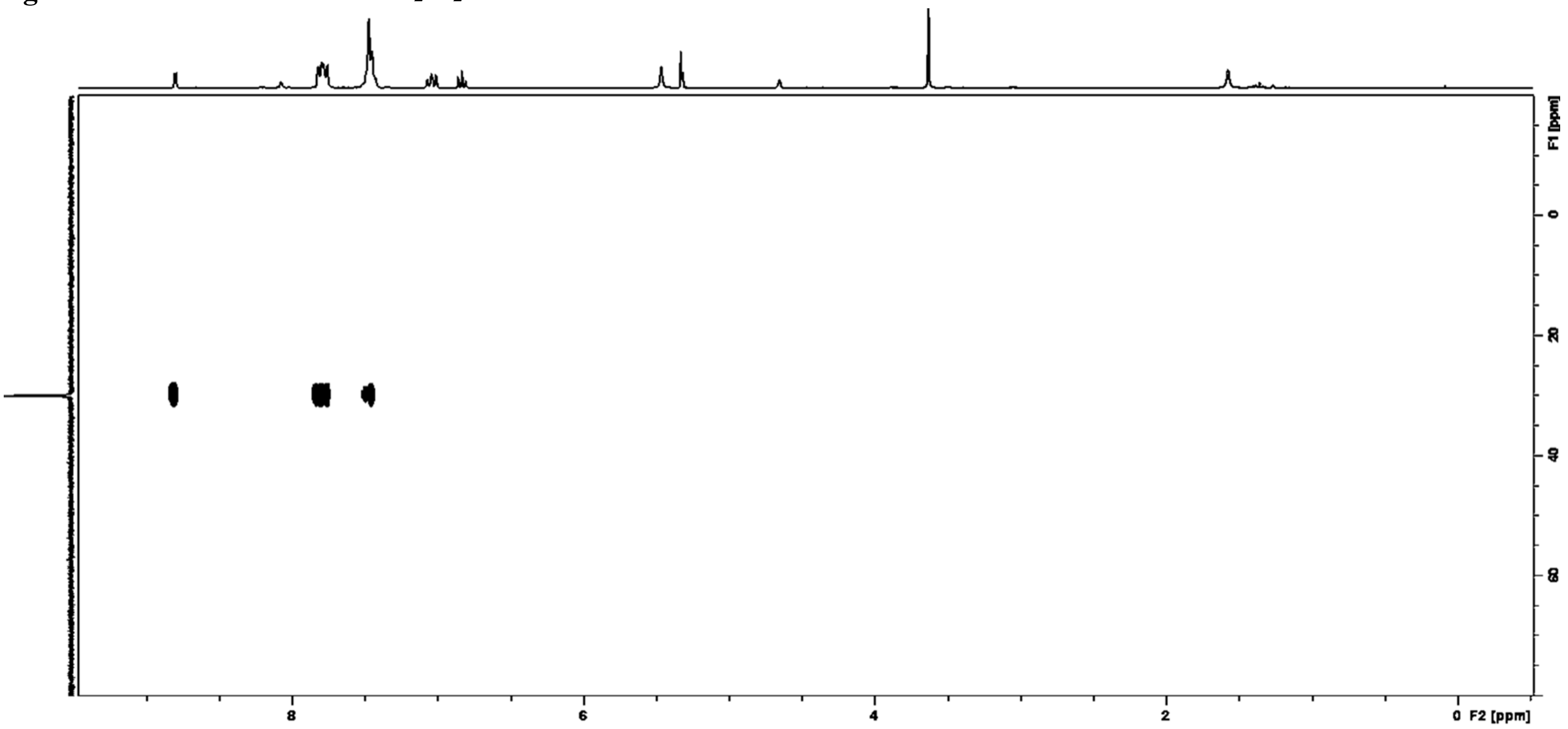

Figure S33. $^{31}P\{^1H\}$ NMR spectrum in CD_2Cl_2 of **Re2**.

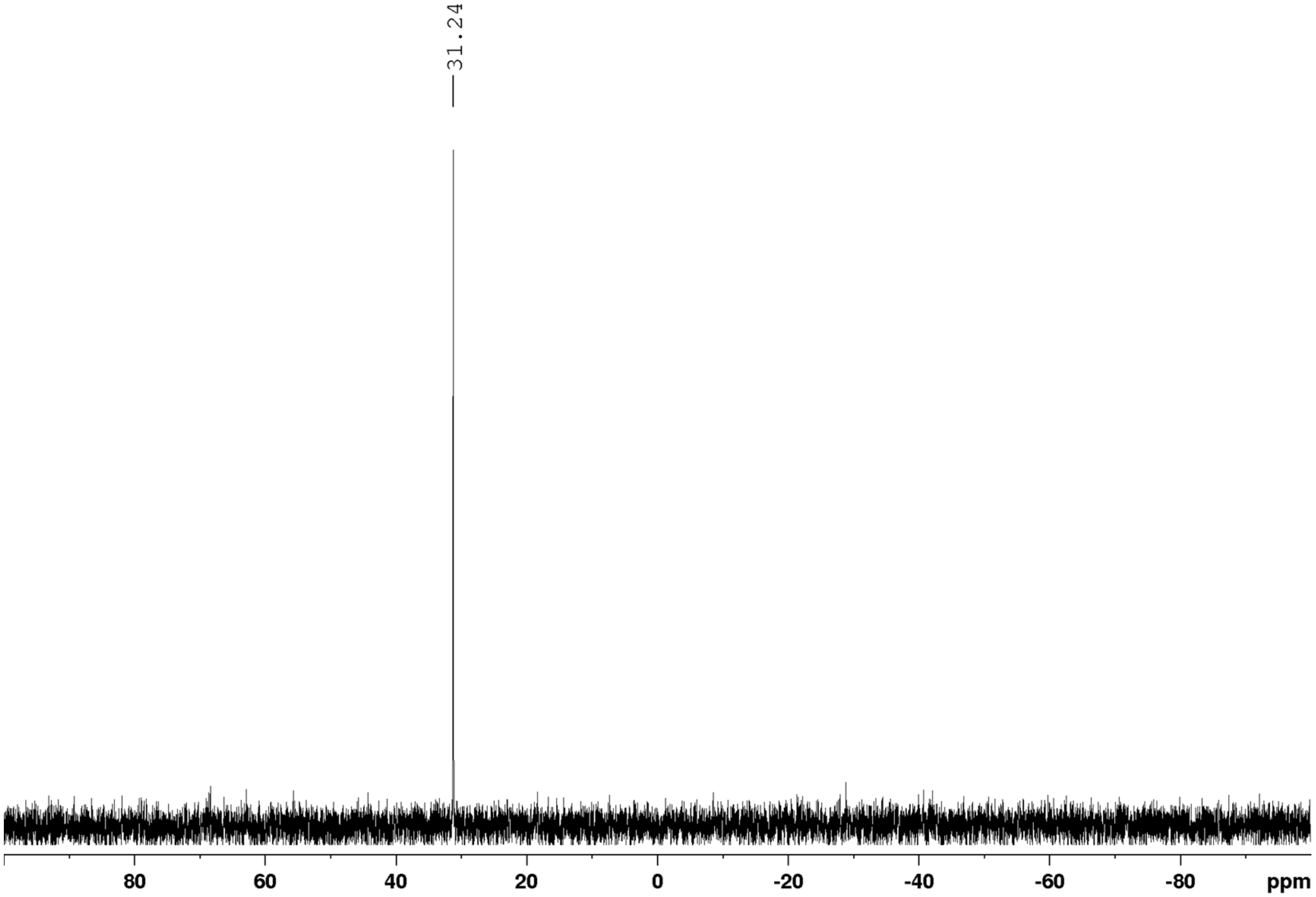

Figure S34. ^{1}H NMR spectrum in CD_2Cl_2 of **Re2**.

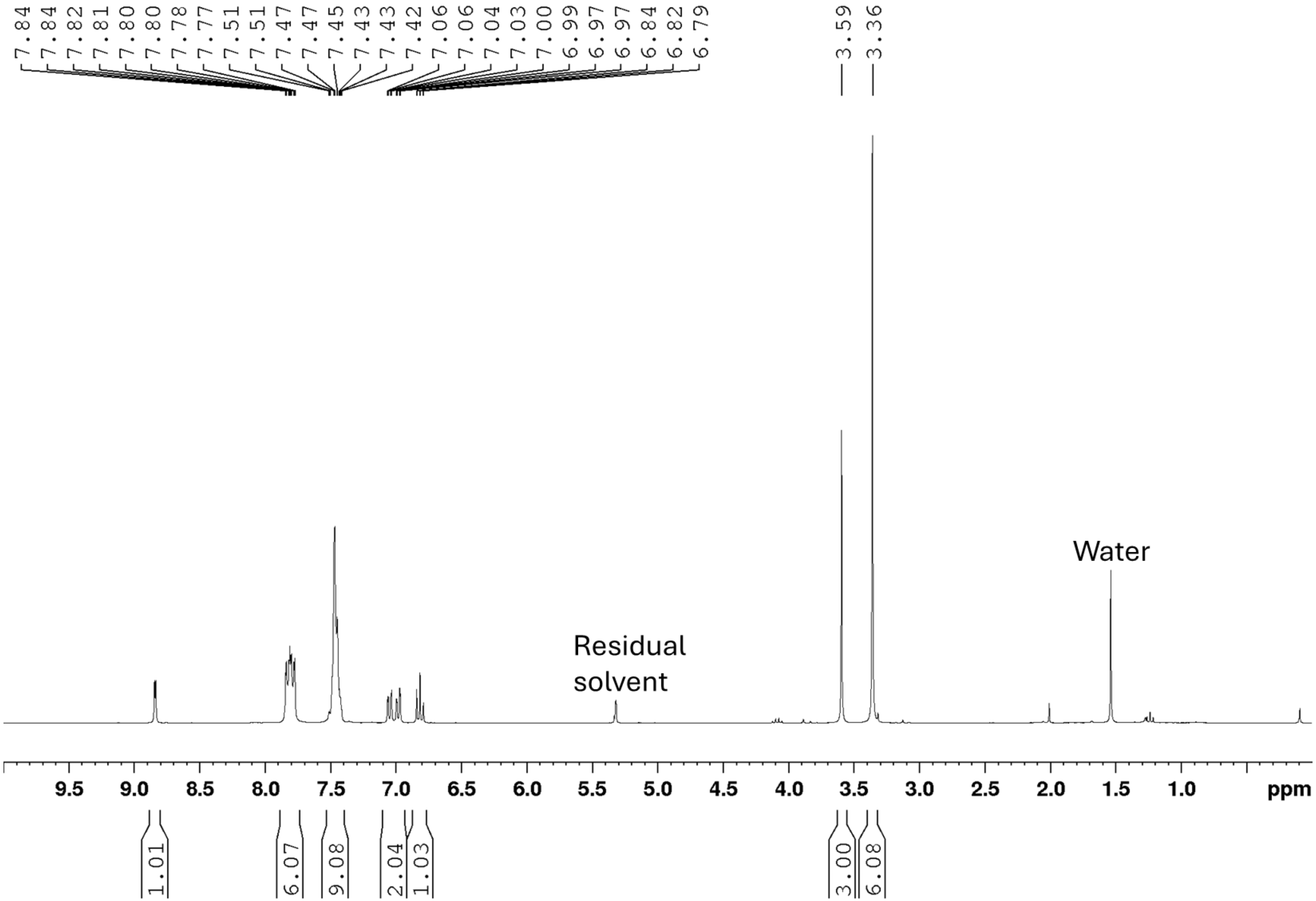

Figure S35. $^{13}C\{^{1}H\}$ NMR spectrum in CD_2Cl_2 of **Re2**.

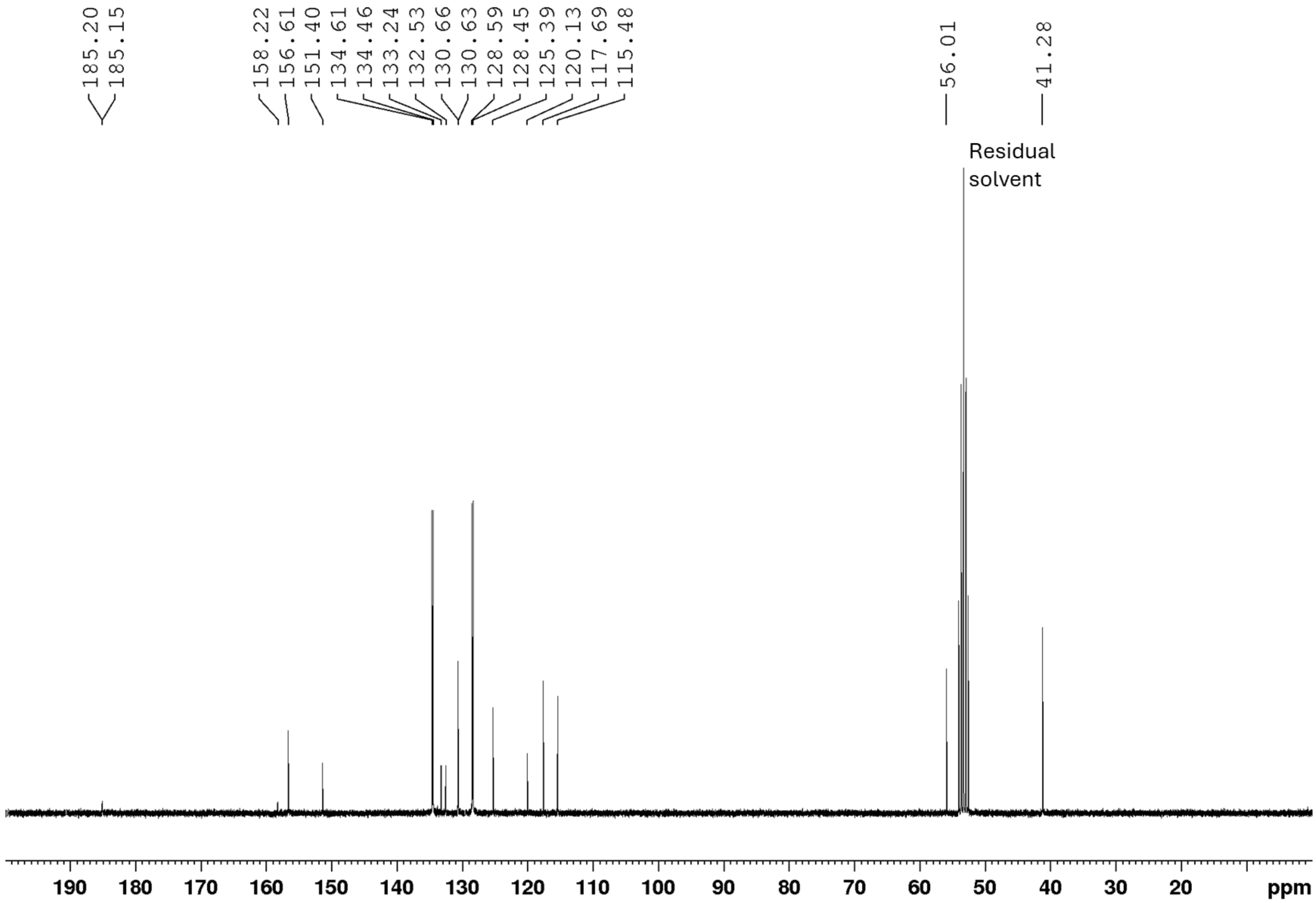

Figure S36. ^{1}H-^{13}C HSQC in CD_2Cl_2 of **Re2**.

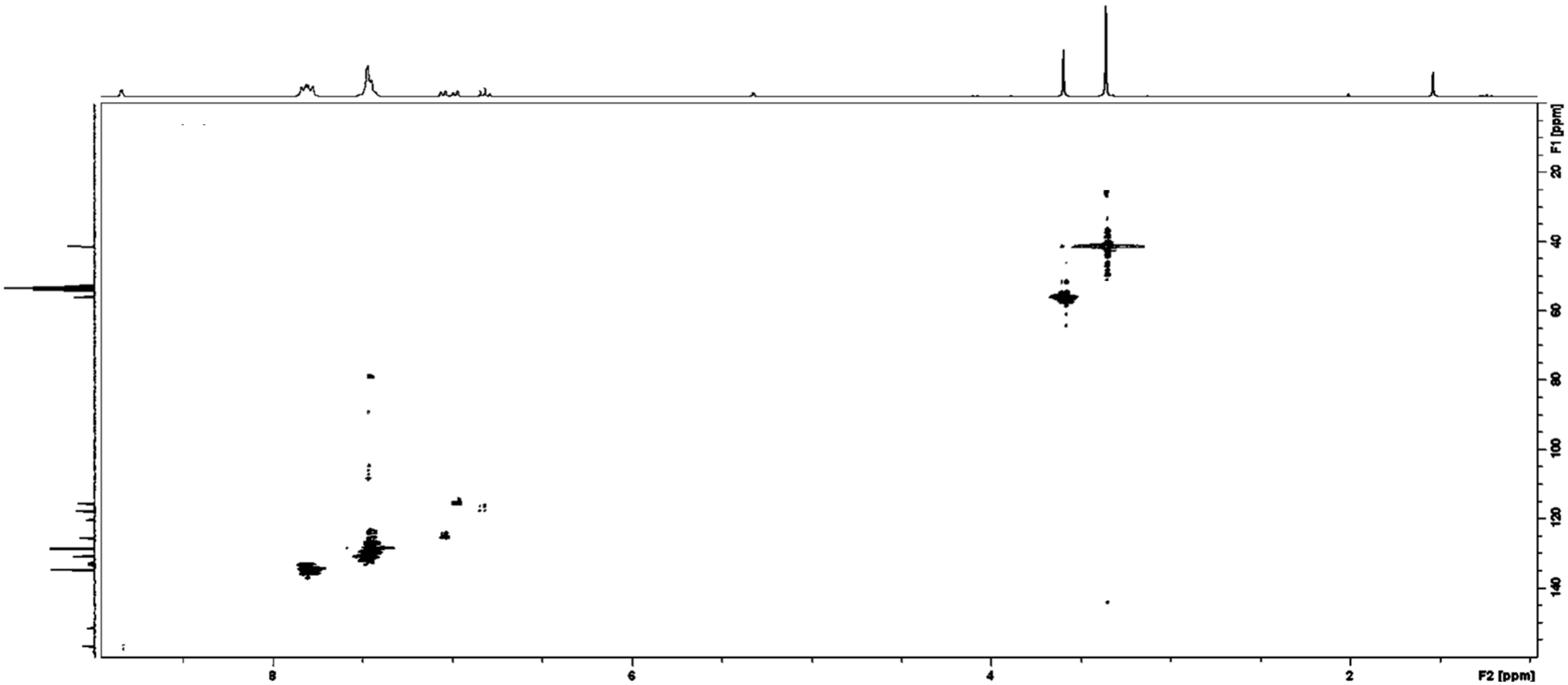

Figure S37. ^{1}H-^{13}C HMBC in CD_2Cl_2 of **Re2**.

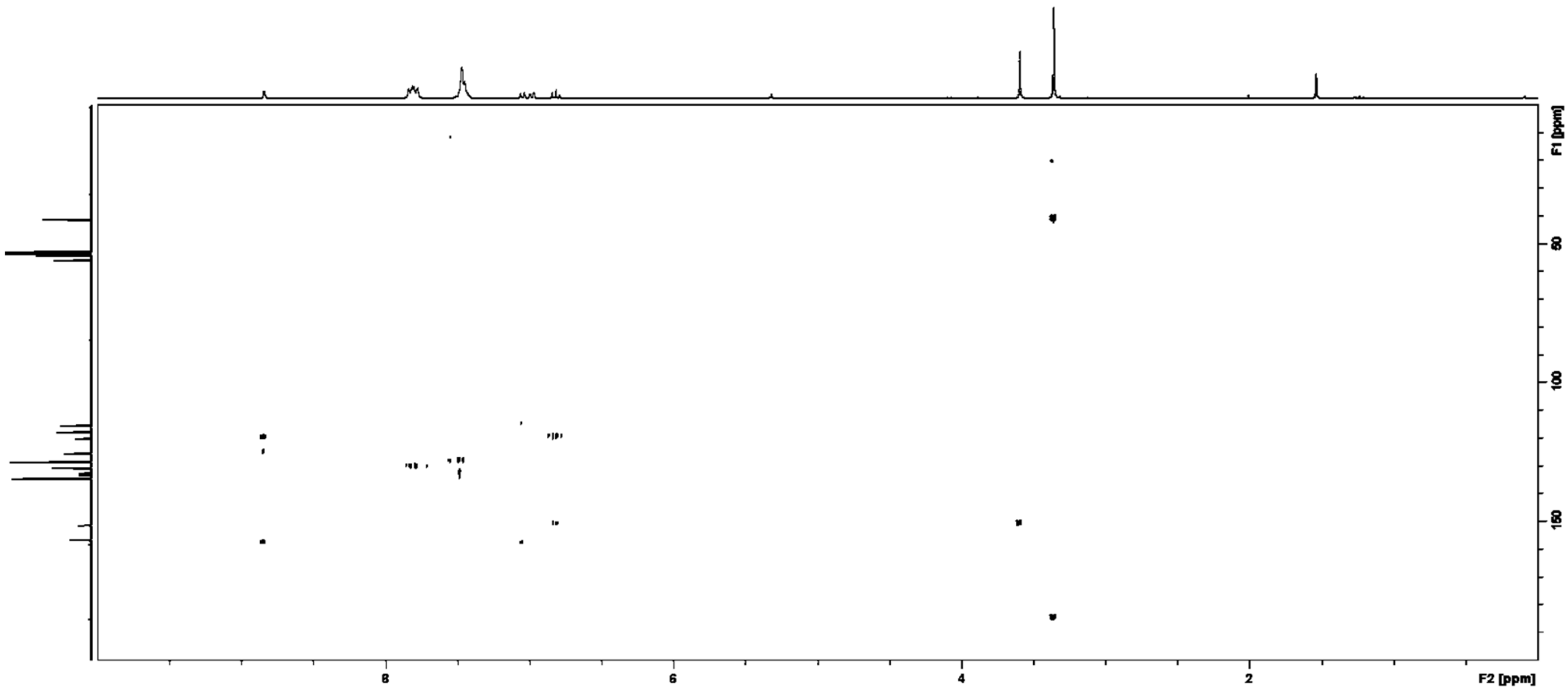

Figure S38. ^{1}H-^{31}P HMBC in CD_2Cl_2 of **Re2**.

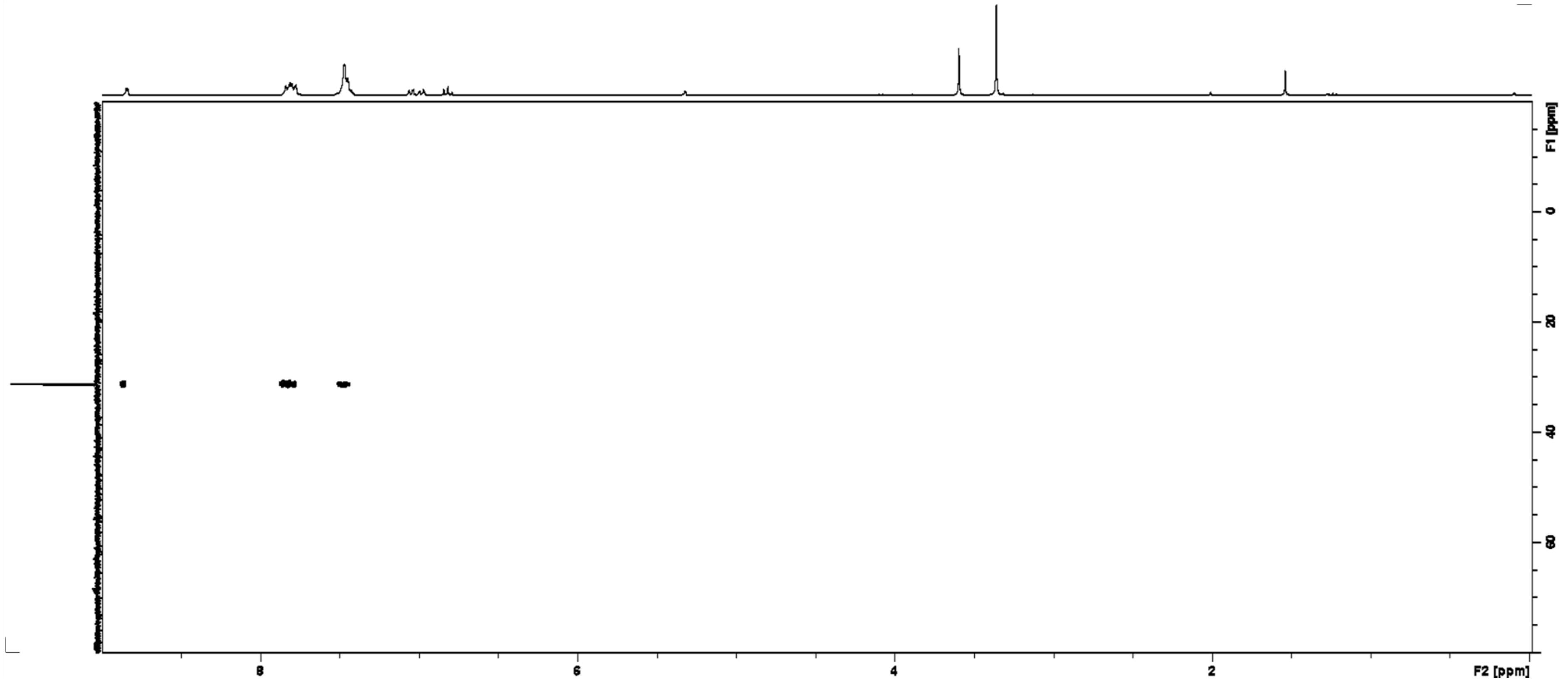

Figure S39. $^{31}P\{^{1}H\}$ NMR spectrum in CD_3CN of **PCN**.

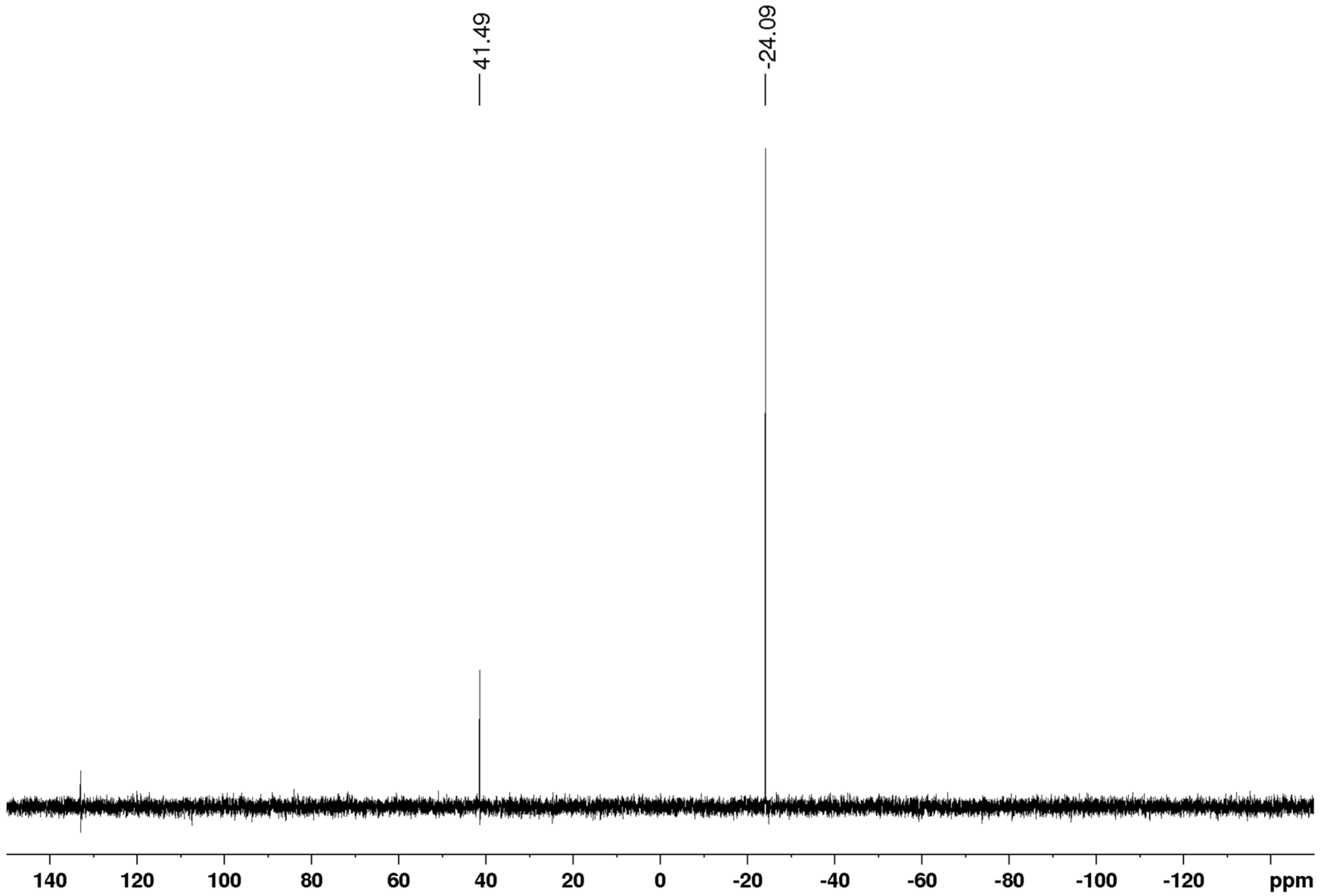

Figure S40. ^{1}H NMR spectrum in CD_2Cl_2 of $\mathbf{H_2L2}$.

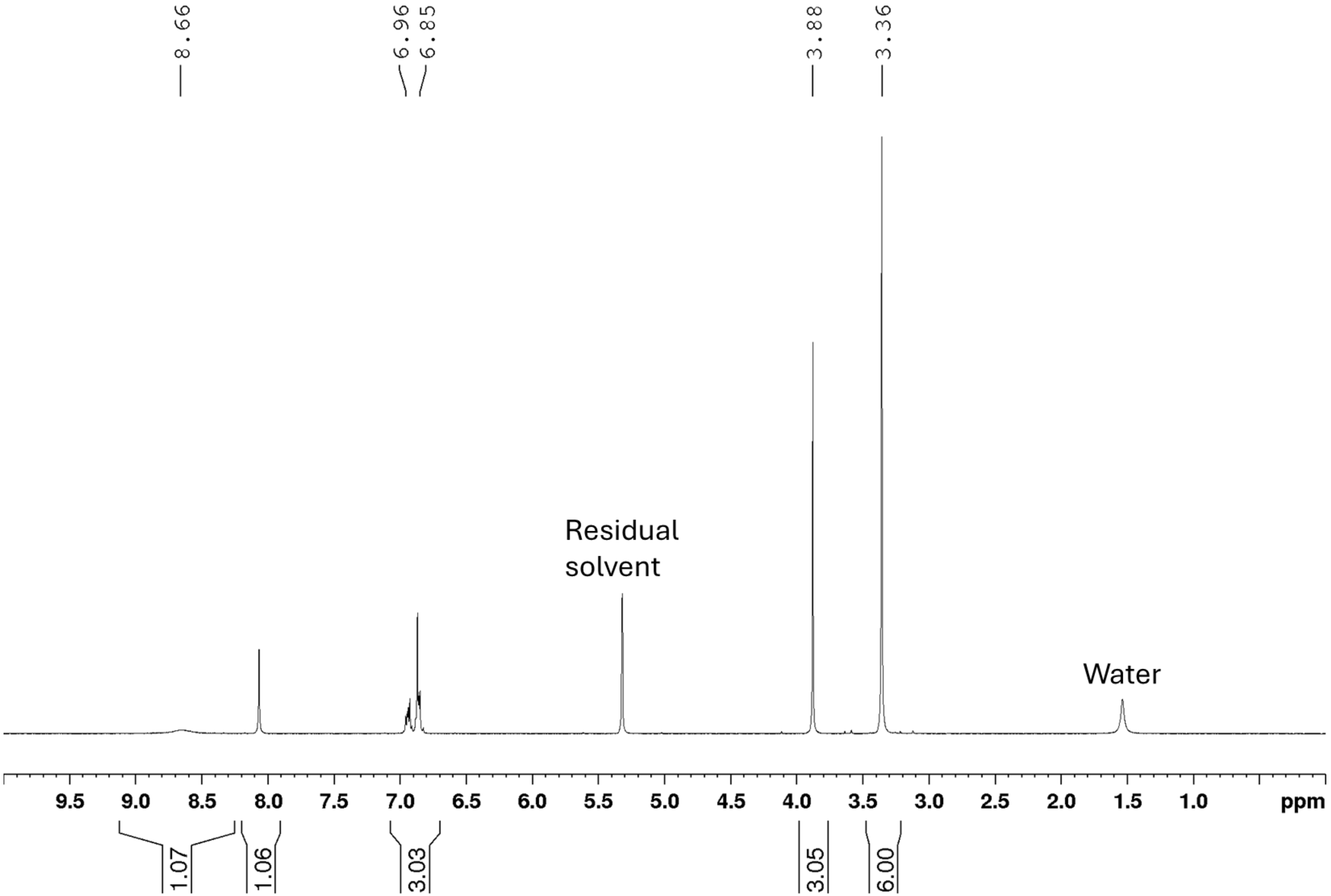

Figure S41. Comparison between ^{1}H NMR spectra (in CD_2Cl_2) of **H_2L2** (red) and **Re2** (blue). Top: 11.6 ppm – 6.6 ppm region; green lines underline the acidic protons of **H_2L2** (absent in **Re2**), the arrow indicates the downfield shift of the azomethinic proton signal in **Re2**. Bottom: 5.6 – 2.6 ppm region; the arrow indicates the upfield shift of the methoxyl proton signal in **Re2**.

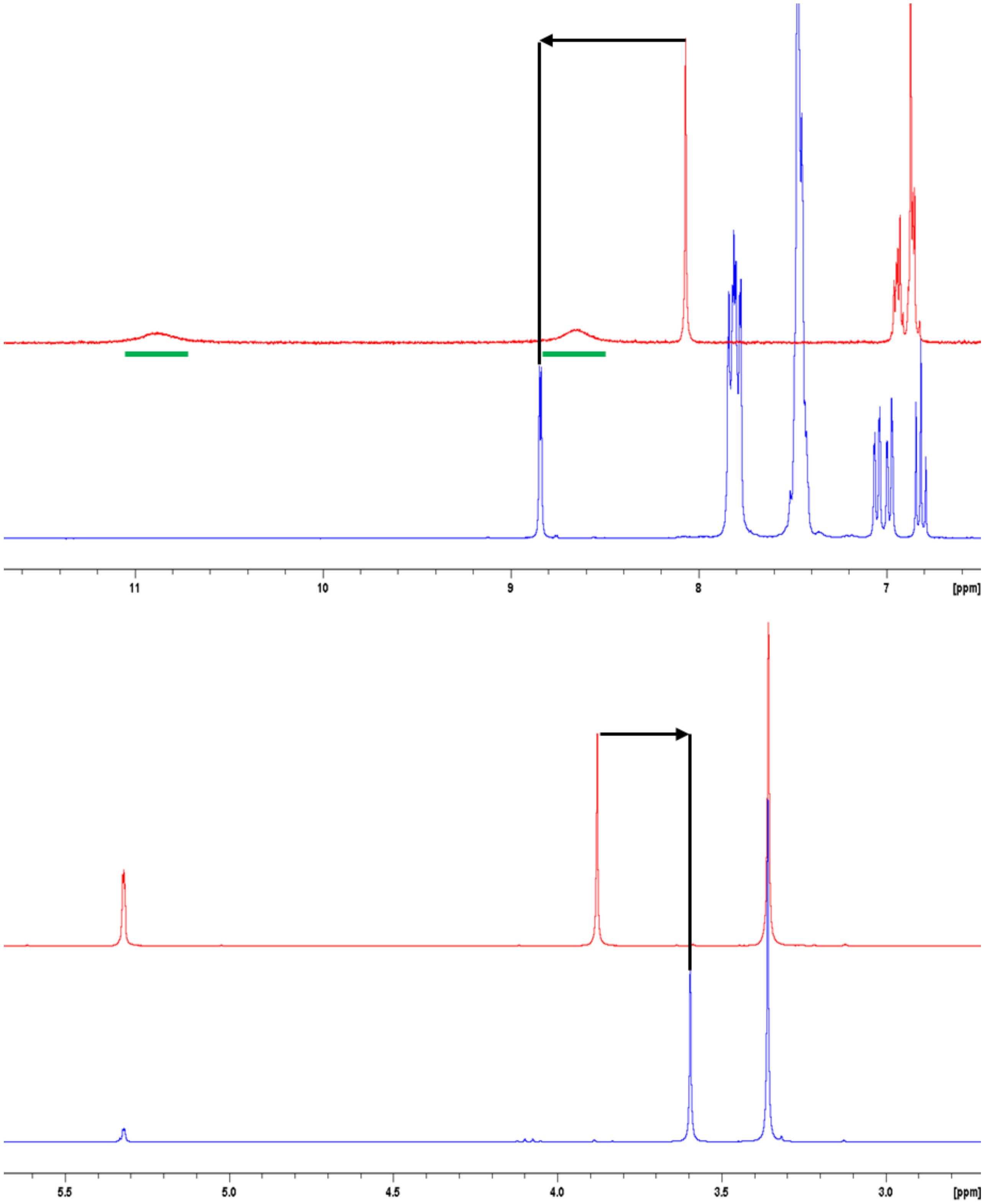

Figure S42. Crystals used in the analysis reported in the main text. Left to right: **H_2L2**, **Re1**, **Re2**. Capillary diameter of 100 μm.

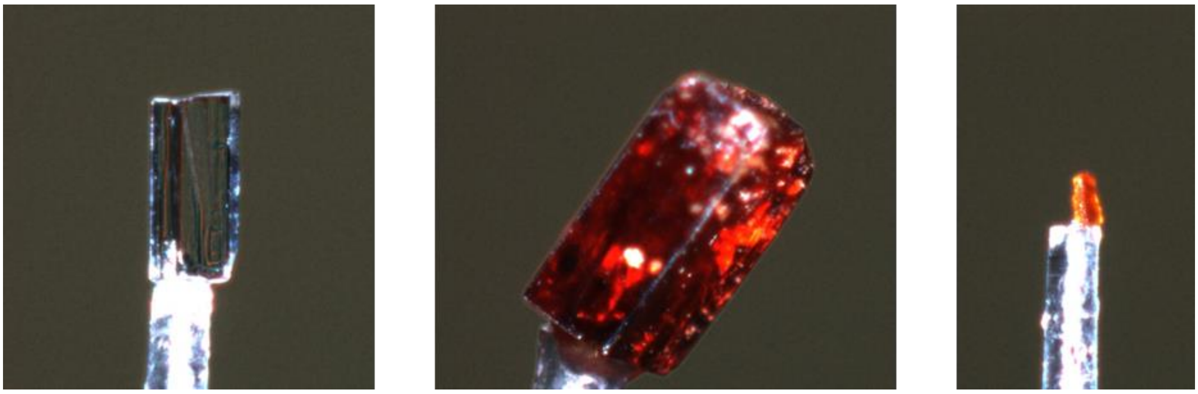

Figure S43. ORTEP diagram of the asymmetric unit of **H_2L2**. Ellipsoids at 50% of probability, H atoms omitted for clarity.

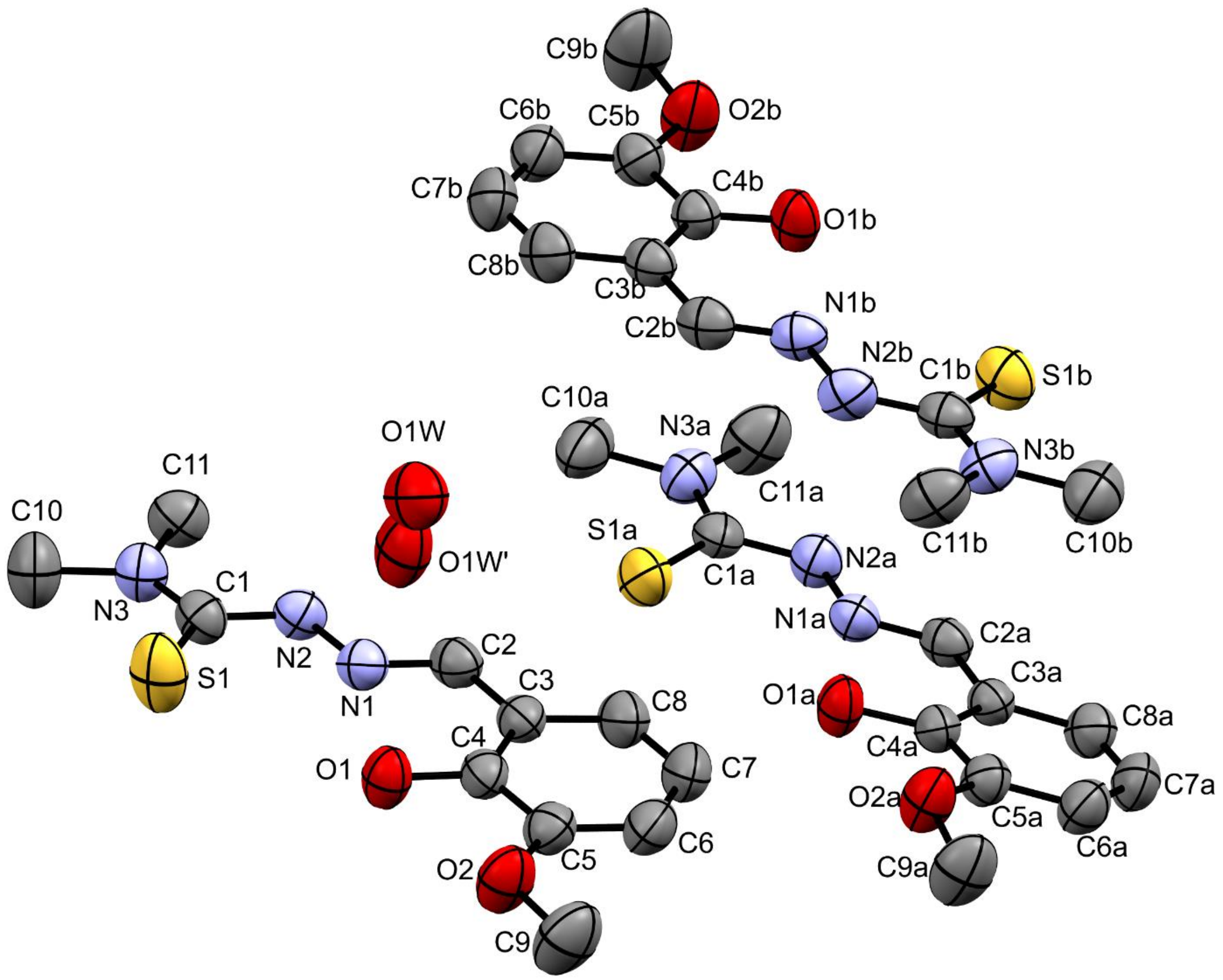

Figure S44. Packing diagram of **H_2L2** viewed along _a_ axis. Non-H ellipsoids at 50% of probability, H omitted for clarity. Molecules aligned into a row are equally coloured.

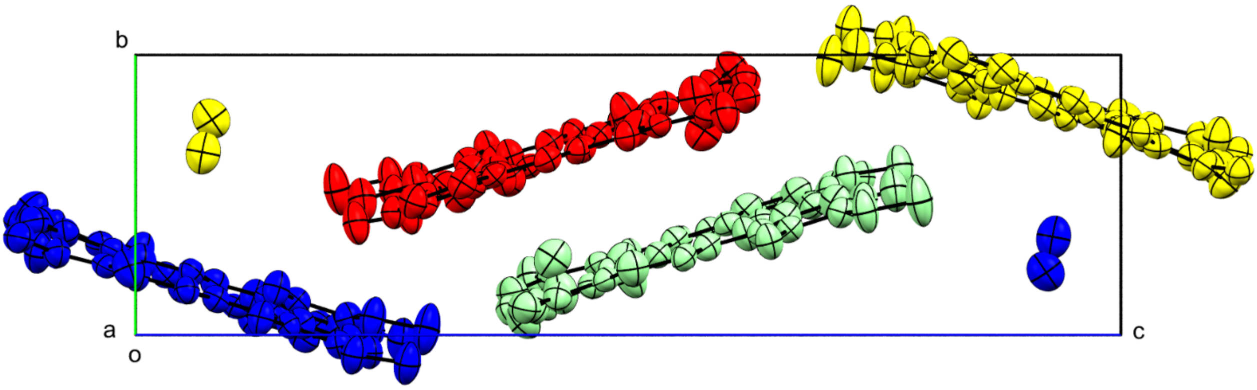

Figure S45. Packing diagram of $\mathbf{H_2L2}$ viewed along _b_ axis. Non-H ellipsoids at 50% of probability, H omitted for clarity. Molecules aligned into a row are equally coloured.

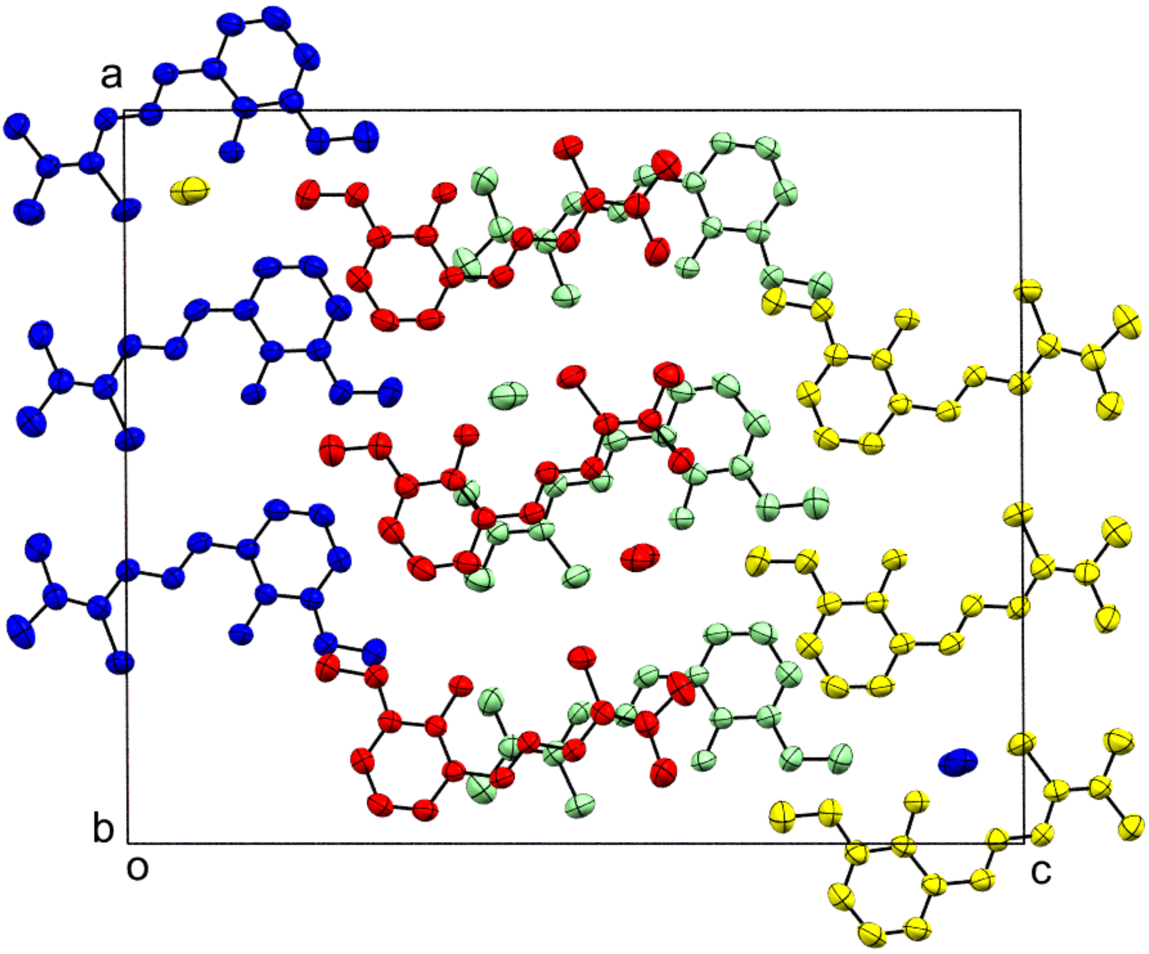

Figure S46. ORTEP diagram of asymmetric unit of **Re2** crystal. Ellipsoids at 30% of probability, H atoms omitted for clarity. Left, the C ("clockwise"), right, the A ("anticlockwise") enantiomers.

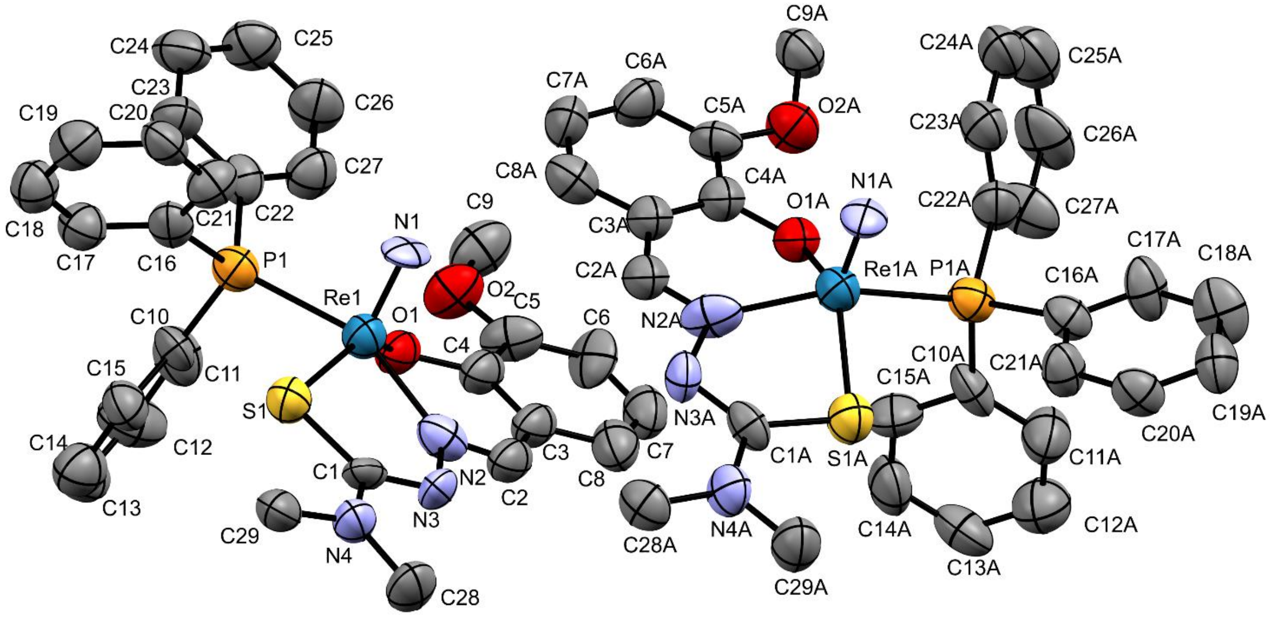

Figure S47. HPLC and LC-MS analyses of the SPE-purified carrier added preparation of $^{99g/99m}$**Tc1**. (A) radio/UV-RP-HPLC chromatogram. (B) TIC/UV-RP-HPLC chromatogram (mass spectrometer in positive mode). (C) ESI(+)-MS spectrum of the peak at 12.09 min in the TIC chromatogram. (D) MS^2 spectrum of the peak at m/z 599.

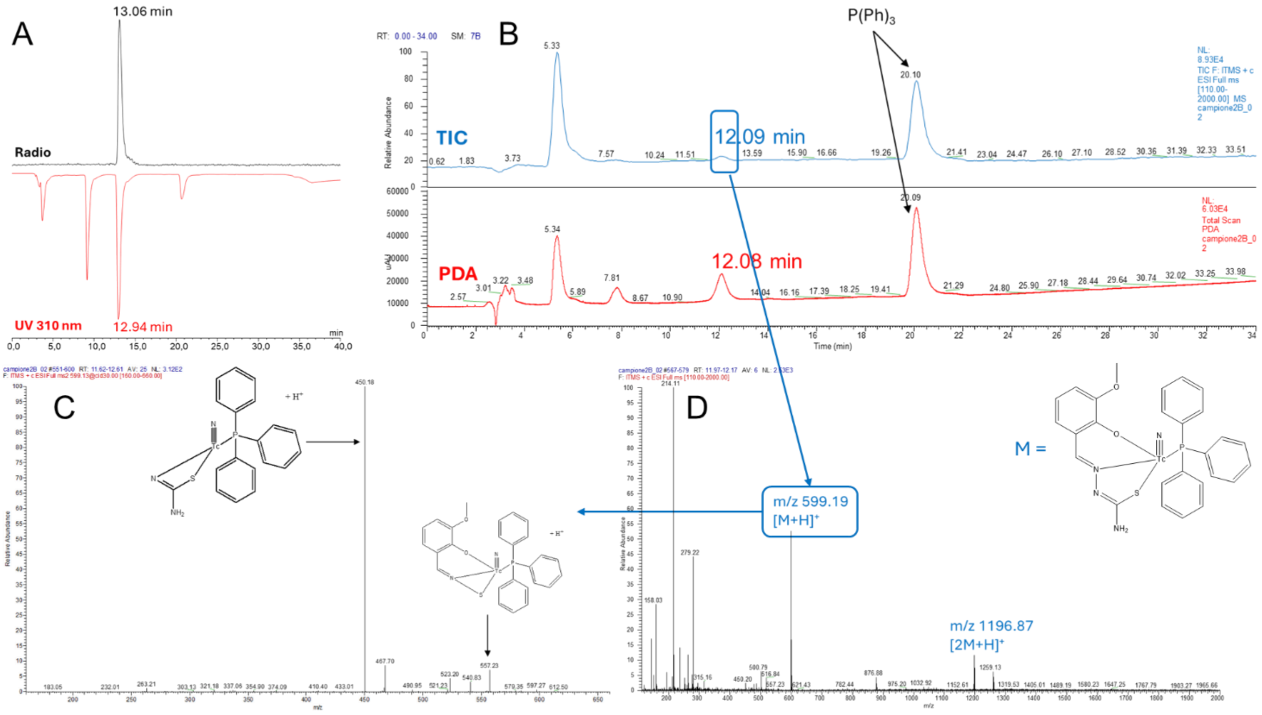

Figure S48. HPLC and LC-MS analyses of the SPE-purified carrier added preparation of $^{99g/99m}$**Tc2**. (A) radio/UV-RP-HPLC chromatogram. (B) TIC/UV-RP-HPLC chromatogram (mass spectrometer in positive mode). (C) ESI(+)-MS spectrum of the peak at 21.35 min in the TIC chromatogram. (D) MS^2 spectrum of the peak at m/z 627.

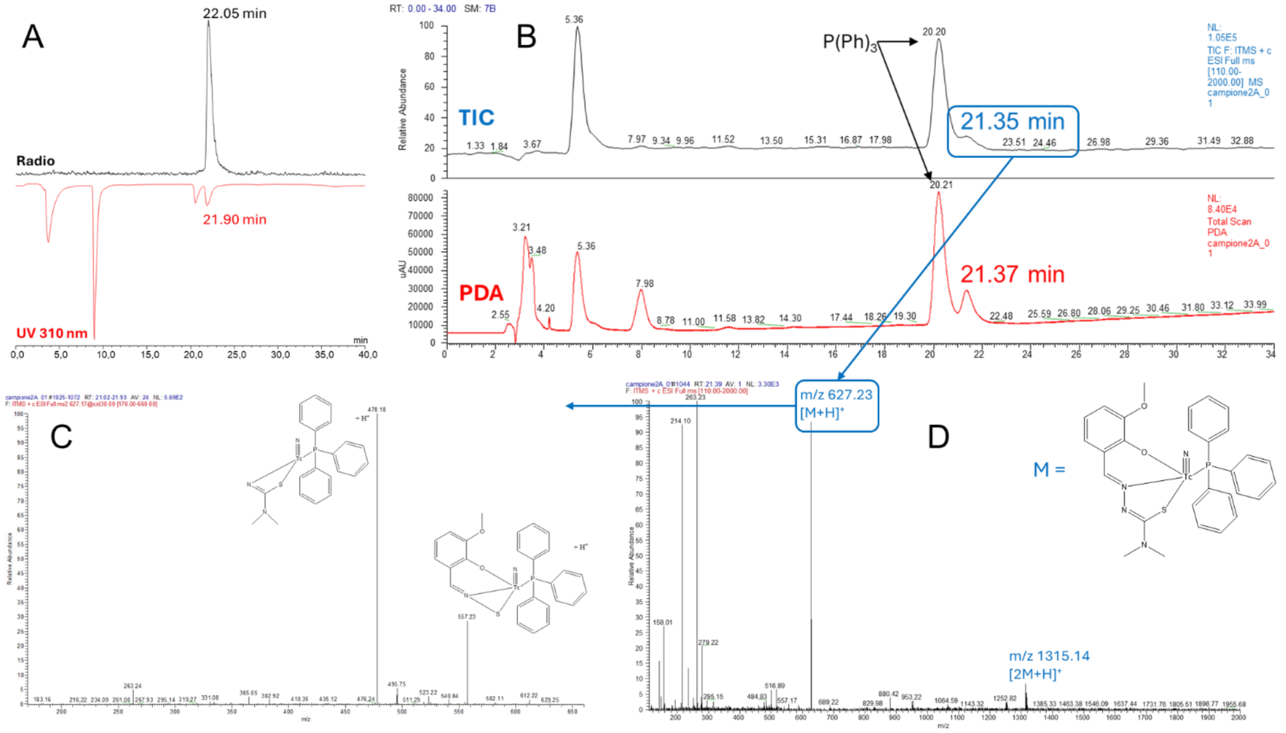

Figure S49. HPLC and LC-MS analyses of the SPE-purified carrier added preparation of $^{99g/99m}$**Tc3**. (A) radio/UV-RP-HPLC chromatogram. (B) TIC/UV-RP-HPLC chromatogram (mass spectrometer in positive mode). (C) ESI(+)-MS spectrum of the peak at 15.95 min in the TIC chromatogram. (D) MS^2 spectrum of the peak at m/z 530.

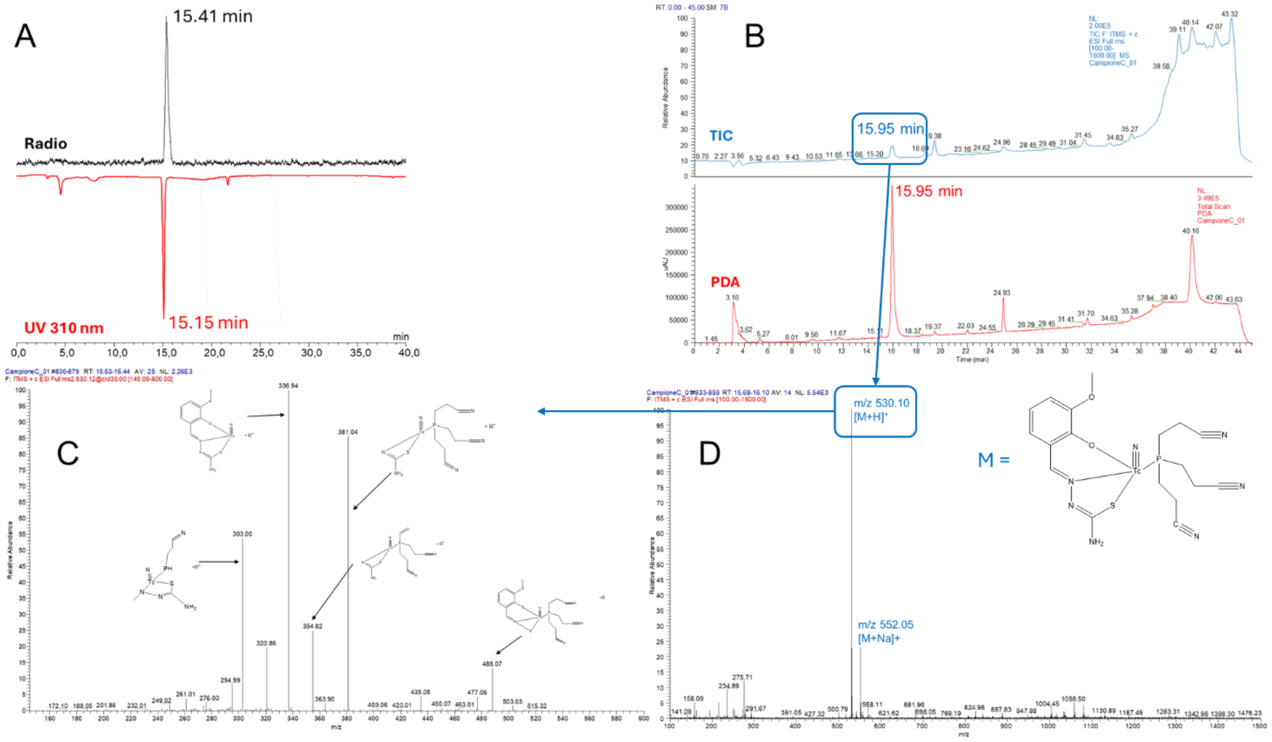

Figure S50. Stability of ^{99m}**Tc1-4** in: phosphate buffer saline (PBS); cysteine (Cys), 1 mM; glutathione (GSH), 1 mM; human serum, type AB (HS).

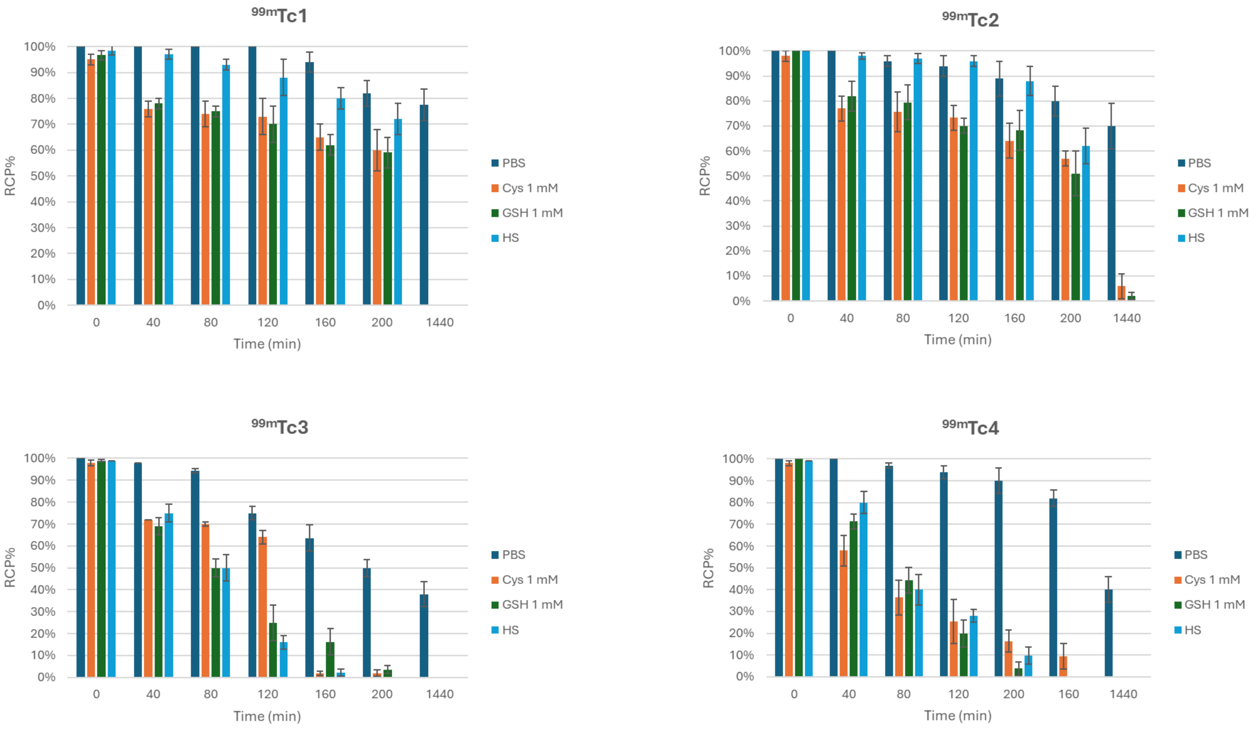

TABLES

Table S1. Principal crystallographic data for **H_2L2**, **Re1** and **Re2**.

	**H_2L2**	**Re1**	**Re2**
**Formula**	$C_{11}H_{15}N_3O_2S \cdot ⅓H_2O$	$C_{27}H_{24}N_4O_2PReS$	$C_{29}H_{28}N_4O_2PReS$
**Formula weight (Da)**	259.32	685.73	713.78
**Temperature (K)**	293(2)	293(2)	293(2)
**Crystal system**	Monoclinic	Monoclinic	Triclinic
**Space group**	$P2_1/n$	$P2_1/n$	$P\bar{1}$
**a (Å)**	20.8659(5)	9.6608(3)	9.5524(5)
**b (Å)**	7.24227(16)	16.7436(6)	17.5477(12)
**c (Å)**	25.5024(5)	16.2967(5)	18.8418(17)
**α (°)**	90	90	117.612(8)
**β (°)**	90.3003(2)	100.231(3)	95.690(6)
**γ (°)**	90	90	92.611(5)
**Cell volume (Å^3)**	3853.78(15)	2594.18(15)	2769.8(4)
**Z, Z'**	12, 3	4, 1	4, 2
**ρ_{calc} (g/cm^3)**	1.341	1.756	1.714
**μ (mm^{-1})**	0.250	4.859	4.554
**$\|F_{000}\|$**	1648.0	1344.0	1408.0
**Crystal size (mm^3)**	0.30·0.12·0.01	0.30·0.15·0.10	0.10.050.01
**Radiation**	Mo Kα (λ=0,71073 nm)	Mo Kα (λ=0,71073 nm)	Mo Kα (λ=0,71073 nm)
**2θ range for data collection (°)**	3.194 to 48.332	3.516 to 63.534	4.308 to 34.558
**Miller indexes ranges**	-24≤h≤23, -8≤k≤7, -28≤l≤28	-13≤h≤14, -24≤k≤23, -23≤l≤23	-7≤h≤7, -14≤k≤14, -15≤l≤15
**Reflections collected**	48027	72838	10832
**Independent reflections**	5289	8411	3330
**R_{int}**	0.0399	0.0460	0.0387
**R_σ**	0.0242	0.0247	0.0402
**S on $\|F\|^2$**	1.051	1.042	1.177
**Data/restr./parameters**	5289/0/497	8411/0/338	3330/27/559
**R_1, wR_2 ($I \geq 2\sigma_I$)**	0.0386, 0.0960	0.0294, 0.0638	0.0588, 0.1446
**R_1, wR_2 (all data)**	0.0572, 0.1031	0.0379, 0.0662	0.0760, 0.1540
**Largest diff./hole (e/Å^3)**	0.16/-0.22	1.97/-1.34	1.12/-0.38

Table S2. Bond lengths in asymmetric unit of **H_2L2**.

Atom	Atom	Length (Å)		Atom	Atom	Length (Å)		Atom	Atom	Length (Å)
S1	C1	1.683(2)		S1a	C1a	1.672(2)		S1b	C1b	1.680(2)
O1	C4	1.354(2)		O1a	C4a	1.358(2)		O1b	C4b	1.359(2)
O2	C5	1.365(3)		O2a	C5a	1.371(2)		O2b	C5b	1.363(3)
O2	C9	1.419(3)		O2a	C9a	1.418(3)		O2b	C9b	1.423(3)
N1	N2	1.365(2)		N1a	N2a	1.361(2)		N1b	N2b	1.369(2)
N1	C2	1.276(3)		N1a	C2a	1.279(2)		N1b	C2b	1.273(3)
N2	C1	1.362(3)		N2a	C1a	1.367(3)		N2b	C1b	1.363(3)
N3	C1	1.338(3)		N3a	C1a	1.338(3)		N3b	C1b	1.340(3)
N3	C10	1.459(3)		N3a	C10a	1.456(3)		N3b	C10b	1.455(3)
N3	C11	1.456(3)		N3a	C11a	1.461(3)		N3b	C11b	1.456(3)
C2	C3	1.450(3)		C2a	C3a	1.452(3)		C2b	C3b	1.445(3)
C3	C4	1.406(3)		C3a	C4a	1.399(3)		C3b	C4b	1.398(3)
C3	C8	1.398(3)		C3a	C8a	1.399(3)		C3b	C8b	1.398(3)
C4	C5	1.401(3)		C4a	C5a	1.399(3)		C4b	C5b	1.400(3)
C5	C6	1.378(3)		C5a	C6a	1.379(3)		C5b	C6b	1.373(3)
C6	C7	1.389(3)		C6a	C7a	1.382(3)		C6b	C7b	1.383(3)
C7	C8	1.362(3)		C7a	C8a	1.367(3)		C7b	C8b	1.361(3)

Table S3. Selected bond lengths in asymmetric unit of **Re1**.

Atom	Atom	Length (Å)		Atom	Atom	Length (Å)
Re1	S1	2.3221(7)		C5	C6	1.382(6)
Re1	P1	2.3923(7)		C6	C7	1.374(5)
Re1	O1	2.030(2)		C7	C8	1.420(4)
Re1	N1	1.654(2)		C10	C11	1.393(4)
Re1	N2	2.110(2)		C10	C15	1.379(4)
S1	C1	1.754(3)		C11	C12	1.380(5)
P1	C10	1.824(3)		C12	C13	1.372(6)
P1	C16	1.826(3)		C13	C14	1.373(6)
P1	C22	1.828(3)		C14	C15	1.394(5)
O1	C8	1.329(3)		C16	C17	1.394(4)
O2	C7	1.371(4)		C16	C21	1.393(4)
O2	C9	1.449(12)		C17	C18	1.388(4)
O2	C9A	1.289(12)		C18	C19	1.387(5)
N2	N3	1.415(3)		C19	C20	1.367(5)
N2	C2	1.292(4)		C20	C21	1.405(4)
N3	C1	1.296(4)		C22	C23	1.380(5)
N4	C1	1.367(4)		C22	C27	1.378(4)
C2	C3	1.432(4)		C23	C24	1.393(5)
C3	C4	1.414(4)		C24	C25	1.375(6)
C3	C8	1.405(4)		C25	C26	1.364(7)
C4	C5	1.372(6)		C26	C27	1.396(5)

Table S4. Selected bond lengths in asymmetric unit of **Re2**.

Atom	Atom	Length (Å)		Atom	Atom	Length (Å)
Re1	S1	2.305(7)		Re1A	S1A	2.307(7)
Re1	P1	2.405(7)		Re1A	P1A	2.396(7)
Re1	O1	2.006(18)		Re1A	O1A	1.995(16)
Re1	N1	1.604(14)		Re1A	N1A	1.572(16)
Re1	N2	2.04(2)		Re1A	N2A	2.06(3)
S1	C1	1.74(3)		S1A	C1A	1.71(3)
P1	C10	1.811(19)		P1A	C10A	1.81(2)
P1	C16	1.804(19)		P1A	C16A	1.799(16)
P1	C22	1.808(18)		P1A	C22A	1.86(2)
O1	C4	1.42(2)		O1A	C4A	1.36(2)
O2	C5	1.34(2)		O2A	C5A	1.38(2)
O2	C9	1.40(3)		O2A	C9A	1.40(3)
N2	N3	1.41(3)		N2A	N3A	1.41(3)
N2	C2	1.32(3)		N2A	C2A	1.32(3)
N3	C1	1.36(3)		N3A	C1A	1.33(3)
N4	C1	1.37(3)		N4A	C1A	1.39(3)
N4	C28	1.42(3)		N4A	C28A	1.42(3)
N4	C29	1.45(3)		N4A	C29A	1.46(3)
C2	C3	1.46(3)		C2A	C3A	1.42(3)
C7	C8	1.3900		C4A	C3A	1.3900
C7	C6	1.3900		C4A	C5A	1.3900
C8	C3	1.3900		C3A	C8A	1.3900
C3	C4	1.3900		C8A	C7A	1.3900
C4	C5	1.3900		C7A	C6A	1.3900
C5	C6	1.3900		C6A	C5A	1.3900
C10	C11	1.3900		C10A	C11A	1.3900
C10	C15	1.3900		C10A	C15A	1.3900
C11	C12	1.3900		C11A	C12A	1.3900
C12	C13	1.3900		C12A	C13A	1.3900
C13	C14	1.3900		C13A	C14A	1.3900
C14	C15	1.3900		C14A	C15A	1.3900
C21	C16	1.3900		C16A	C17A	1.3900
C21	C20	1.3900		C16A	C21A	1.3900
C16	C17	1.3900		C17A	C18A	1.3900
C17	C18	1.3900		C18A	C19A	1.3900
C18	C19	1.3900		C19A	C20A	1.3900
C19	C20	1.3900		C20A	C21A	1.3900
C22	C23	1.3900		C22A	C23A	1.3900
C22	C27	1.3900		C22A	C27A	1.3900
C23	C24	1.3900		C23A	C24A	1.3900
C24	C25	1.3900		C24A	C25A	1.3900
C25	C26	1.3900		C25A	C26A	1.3900
C26	C27	1.3900		C26A	C27A	1.3900

Table S5. Bond angles in asymmetric unit of **Re1**.

Atom	Atom	Atom	Angle (˚)		Atom	Atom	Atom	Angle (˚)
S1	Re1	P1	94.03(3)		C4	C5	C6	120.1(4)
O1	Re1	S1	139.26(7)		C7	C6	C5	121.1(3)
O1	Re1	P1	86.59(6)		O2	C7	C6	122.3(3)
O1	Re1	N2	85.25(9)		O2	C7	C8	117.2(3)
N1	Re1	S1	108.06(9)		C6	C7	C8	120.4(3)
N1	Re1	P1	95.54(10)		O1	C8	C3	124.6(3)
N1	Re1	O1	112.42(11)		O1	C8	C7	117.1(3)
N1	Re1	N2	104.69(12)		C3	C8	C7	118.2(3)
N2	Re1	S1	80.35(7)		C11	C10	P1	120.0(2)
N2	Re1	P1	159.76(7)		C15	C10	P1	121.3(2)
C1	S1	Re1	97.93(11)		C15	C10	C11	118.6(3)
C10	P1	Re1	116.67(9)		C12	C11	C10	121.1(3)
C10	P1	C16	106.59(13)		C13	C12	C11	120.0(3)
C10	P1	C22	103.34(13)		C12	C13	C14	119.6(3)
C16	P1	Re1	110.11(9)		C13	C14	C15	120.8(3)
C16	P1	C22	104.41(13)		C10	C15	C14	119.9(3)
C22	P1	Re1	114.69(10)		C17	C16	P1	118.0(2)
C8	O1	Re1	126.08(18)		C21	C16	P1	122.7(2)
C7	O2	C9	118.1(8)		C21	C16	C17	119.3(3)
C9A	O2	C7	121.5(6)		C18	C17	C16	120.0(3)
N3	N2	Re1	121.36(18)		C19	C18	C17	120.5(3)
C2	N2	Re1	124.0(2)		C20	C19	C18	119.9(3)
C2	N2	N3	114.3(2)		C19	C20	C21	120.5(3)
C1	N3	N2	112.9(2)		C16	C21	C20	119.8(3)
N3	C1	S1	125.3(2)		C23	C22	P1	118.6(2)
N3	C1	N4	119.1(3)		C27	C22	P1	122.8(3)
N4	C1	S1	115.6(3)		C27	C22	C23	118.6(3)
N2	C2	C3	126.0(3)		C22	C23	C24	121.3(4)
C4	C3	C2	117.2(3)		C25	C24	C23	119.4(4)
C8	C3	C2	122.5(3)		C26	C25	C24	119.9(4)
C8	C3	C4	119.8(3)		C25	C26	C27	120.8(4)
C5	C4	C3	120.3(4)		C22	C27	C26	120.0(4)

Table S6. Bond angles in asymmetric unit of **Re2**.

Atom	Atom	Atom	Angle (˚)		Atom	Atom	Atom	Angle (˚)
S1	Re1	P1	90.2(2)		S1A	Re1A	P1A	95.1(2)
O1	Re1	S1	142.1(5)		O1A	Re1A	S1A	141.0(5)
O1	Re1	P1	88.0(4)		O1A	Re1A	P1A	84.1(5)
O1	Re1	N2	85.8(8)		O1A	Re1A	N2A	86.8(8)
N1	Re1	S1	105.1(7)		N1A	Re1A	S1A	107.0(7)
N1	Re1	P1	96.4(6)		N1A	Re1A	P1A	97.6(7)
N1	Re1	O1	112.7(8)		N1A	Re1A	O1A	111.8(8)
N1	Re1	N2	108.3(8)		N1A	Re1A	N2A	105.3(9)
N2	Re1	S1	80.1(7)		N2A	Re1A	S1A	79.0(7)
N2	Re1	P1	154.9(6)		N2A	Re1A	P1A	157.1(6)
C1	S1	Re1	99.7(15)		C1A	S1A	Re1A	98.6(13)
C10	P1	Re1	107.3(6)		C10A	P1A	Re1A	111.0(11)
C16	P1	Re1	113.7(12)		C10A	P1A	C22A	105.6(15)
C16	P1	C10	109.0(17)		C16A	P1A	Re1A	119.4(7)
C16	P1	C22	101.6(11)		C16A	P1A	C10A	101.7(14)
C22	P1	Re1	120.2(10)		C16A	P1A	C22A	104.4(9)
C22	P1	C10	104.4(13)		C22A	P1A	Re1A	113.2(10)
C4	O1	Re1	124.6(12)		C4A	O1A	Re1A	128.0(14)
C5	O2	C9	117(2)		C5A	O2A	C9A	118(2)
N3	N2	Re1	124.8(18)		N3A	N2A	Re1A	125.2(17)
C2	N2	Re1	126(2)		C2A	N2A	Re1A	125.1(19)
C2	N2	N3	108(3)		C2A	N2A	N3A	110(3)
C1	N3	N2	111(2)		C1A	N3A	N2A	109(2)
C1	N4	C28	124(2)		C1A	N4A	C28A	123(2)
C1	N4	C29	120(3)		C1A	N4A	C29A	121(3)
C28	N4	C29	116(3)		C28A	N4A	C29A	116(3)
N3	C1	S1	122(3)		N3A	C1A	S1A	126(2)
N3	C1	N4	116(2)		N3A	C1A	N4A	115(2)
N4	C1	S1	122(3)		N4A	C1A	S1A	119(3)
N2	C2	C3	125(3)		N2A	C2A	C3A	125(3)
C8	C7	C6	120.0		O1A	C4A	C3A	120.4(19)
C3	C8	C7	120.0		O1A	C4A	C5A	119.6(19)
C8	C3	C2	116.0(18)		C3A	C4A	C5A	120.0
C8	C3	C4	120.0		C4A	C3A	C2A	126(2)
C4	C3	C2	123.9(18)		C4A	C3A	C8A	120.0
C3	C4	O1	120.2(15)		C8A	C3A	C2A	114(2)
C3	C4	C5	120.0		C7A	C8A	C3A	120.0
C5	C4	O1	119.8(15)		C8A	C7A	C6A	120.0
O2	C5	C4	113.5(17)		C5A	C6A	C7A	120.0
O2	C5	C6	126.5(17)		O2A	C5A	C4A	114(2)
C4	C5	C6	120.0		O2A	C5A	C6A	126(2)
C5	C6	C7	120.0		C6A	C5A	C4A	120.0
C11	C10	P1	122(3)		C11A	C10A	P1A	123(3)
C11	C10	C15	120.0		C11A	C10A	C15A	120.0
C15	C10	P1	118(3)		C15A	C10A	P1A	117(3)
C12	C11	C10	120.0		C12A	C11A	C10A	120.0

C11	C12	C13	120.0		C11A	C12A	C13A	120.0
C12	C13	C14	120.0		C14A	C13A	C12A	120.0
C15	C14	C13	120.0		C13A	C14A	C15A	120.0
C14	C15	C10	120.0		C14A	C15A	C10A	120.0
C16	C21	C20	120.0		C17A	C16A	P1A	121.1(13)
C21	C16	P1	118(3)		C17A	C16A	C21A	120.0
C21	C16	C17	120.0		C21A	C16A	P1A	118.5(13)
C17	C16	P1	122(3)		C18A	C17A	C16A	120.0
C18	C17	C16	120.0		C19A	C18A	C17A	120.0
C17	C18	C19	120.0		C18A	C19A	C20A	120.0
C20	C19	C18	120.0		C19A	C20A	C21A	120.0
C19	C20	C21	120.0		C20A	C21A	C16A	120.0
C23	C22	P1	121.0(18)		C23A	C22A	P1A	116(3)
C23	C22	C27	120.0		C23A	C22A	C27A	120.0
C27	C22	P1	119.0(18)		C27A	C22A	P1A	124(3)
C24	C23	C22	120.0		C24A	C23A	C22A	120.0
C23	C24	C25	120.0		C23A	C24A	C25A	120.0
C26	C25	C24	120.0		C24A	C25A	C26A	120.0
C25	C26	C27	120.0		C27A	C26A	C25A	120.0
C26	C27	C22	120.0		C26A	C27A	C22A	120.0

OTHER INFORMATION

Attempts of synthesis of [ReN(L1)PCN]

Attempts were made to obtain the PCN-based complex [ReN(L1)PCN].

The general procedure was as follows; variable parameters are specified in the table on the next page.

General procedure. A specified quantity of the relevant precursor complex (Parameter A) and PCN (Parameter B) were suspended or dissolved in a specified volume of the relevant dinitrogen-saturated solvent (Parameter C) within a dinitrogen-saturated two-necked flask. Subsequently, a solution containing a specified quantity of H_2L1 (Parameter D) and triethylamine (Parameter E) in a specified solvent (Parameter F) was added. The final mixture was stirred under defined conditions (Parameter G).

The reactions were monitored by TLC, and none showed the formation of a complex with chromatographic properties similar to the corresponding technetium-99m complex (**^{99m}Tc3**, as referenced in the main text). Reactions starting from $[ReNCl_2(PCN)_2]$ (**Re0**, as referenced in the main text) indicated the release of PCN into the solution.

A (mg; μmol)	*B (mg; eq.)*	*C (mL, temperature of the final mixture)*	*D (mg; eq.)*	*E (mL)*	*F (mL, temperature of the final solution)*	*G (time, temperature)*	*NOTES*
$(NBu_4)[ReNCl_4]$ (17.89; 30.5)	17.8; 2.80	Me_2CO (2, RT)	16.3; 2.4	1.2	CH_2Cl_2 (2, RT)	1.3 h, reflux	-
$(NBu_4)[ReNCl_4]$ (17.8; 30.5)	17.8; 2.80	Me_2CO (2, b.p.)	16.3; 2.4	1.2	CH_2Cl_2 (5, RT)	1.3 h, reflux	The H_2L1/triethylamine solution was added after the mixture in the flask turned yellow-orange, indicating reduction.
$[ReNCl_2(PCN)_2]$ (13.2; 24.7)	0	CH_2Cl_2 (4, RT)	13.9; 2.5	0.9	MeOH (4, b.p.)	2.7 h, reflux	-
$[ReNCl_2(PCN)_2]$ (15.0; 22.8)	0	MeCN (2, RT)	3.9; 1.05	0.4	MeCN (2, b.p.)	1 h, reflux	-
$[ReNCl_2(PCN)_2]$ (10.8; 16.4)	0	MeCN (3, RT)	3.9; 1.05	0.6	MeCN (2, RT)	190 h, RT	-
$[ReNCl_2(PCN)_2]$ (15.0; 22.8)	0	Me_2CO (3, RT)	5.5; 1.07	0.9	Me_2CO (4, RT)	6, reflux	The H_2L1/NEt_3 solution was added in 0.5 mL aliquots every five minutes

RT = room temperature, roughly 23 °C; b.p. = boiling point.
