# Supplementary material for: Synthesis, Characterisation and Structural Analysis of Rhenium and Technetium Nitride Complexes With Tridentate Thiosemicarbazone‐Phenols and Phosphine Ligands: Potential Applications in Technetium‐99m Radiotracer Development
Source: Bioinorg Chem Appl. 2026 Jul 22;2026:3202767. doi: 10.1155/bca/3202767 (PMC13390190; doi:10.1155/bca/3202767)

checkCIF/PLATON report

Structure factors have been supplied for datablock(s) 32_xx1_twin_nowob_nofried_twin1_hklf4_088

THIS REPORT IS FOR GUIDANCE ONLY. IF USED AS PART OF A REVIEW PROCEDURE FOR PUBLICATION, IT SHOULD NOT REPLACE THE EXPERTISE OF AN EXPERIENCED CRYSTALLOGRAPHIC REFEREE.

No syntax errors found.   CIF dictionary   Interpreting this report

Datablock: 32_xx1_twin_nowob_nofried_twin1_hklf4_088

```
Bond precision:   C-C = 0.0418 A          Wavelength=0.71073

Cell:           a=9.5524(5)        b=17.5477(12)     c=18.8418(17)
                alpha=117.612(8)   beta=95.690(6)    gamma=92.611(5)
Temperature:    293 K

                 Calculated                   Reported
Volume           2769.8(4)                    2769.8(4)
Space group      P -1                         P -1
Hall group       -P 1                         -P 1
Moiety formula   C29 H28 N4 O2 P Re S         C29 H28 N4 O2 P Re S
Sum formula      C29 H28 N4 O2 P Re S         C29 H28 N4 O2 P Re S
Mr               713.79                       713.78
Dx,g cm-3        1.712                        1.712
Z                4                            4
Mu (mm-1)        4.554                        4.554
F000             1408.0                       1408.0
F000'            1405.33
h,k,lmax         7,14,15                      7,14,15
Nref             3374                         3330
Tmin,Tmax                                     0.001,0.004
Tmin'

Correction method= # Reported T Limits: Tmin=0.001 Tmax=0.004
AbsCorr = SPHERE

Data completeness= 0.987          Theta(max)= 17.279

                                                      wR2(reflections)=
R(reflections)= 0.0588( 2622)                         0.1540( 3330)

S = 1.177                  Npar= 559
```

The following ALERTS were generated. Each ALERT has the format
test-name_ALERT_alert-type_alert-level.
Click on the hyperlinks for more details of the test.

Alert level A

THETM01_ALERT_3_A The value of sine(theta_max)/wavelength is less than 0.550
Calculated sin(theta_max)/wavelength = 0.4179

Author Response: Despite several crystallization attempts, the only usable specimen was a very thin plate, which eventually turned out to be a non-merohedral twin. The diffracting ability of the sample was low and virtually no useful information is available at a resolution higher than 1.20 angstroms. Also above this threshold the signal-to-noise ratio is very low and the weak signals hardly emerge over the background. Besides, the proposed solution is based on the refinement of the data pertaining to the major twin component, which was showing a better Rint value. Accordingly, the quality of the manageable data is far from optimal. This explains the very low value of the ratio. As a further consequence, data redundancy was also appreciably reduced and this accounts for the PLAT088 Alert below.

PLAT088_ALERT_3_A Poor Data / Parameter Ratio .... 5.96 Note

Author Response: See comments made above for the THETM01 Alert.

PLAT234_ALERT_4_A Large Hirshfeld Difference C14A --C15A . 0.32 Ang.

Author Response: This issue is also determined by the same reasons above underlined for the other type A Alerts above. Several attempts were made to model the phenyl rings of the triphenylphosphine and of the thiosemicarbazonate by introducing RIGU restraints or a combination of RIGU restraints and EADP constraints, but these strategies did not allow to reach a final model incorporating anisotropic treatment of non-hydrogen atoms. At the end, we were forced to impose also AFIX constraints to the above molecular fragments; nevertheless, the ring carbon atoms remained affected by significant thermal motions and the paucity of the data did not allow to further attenuate differences in the displacement parameters. The same applies to the remaining PLAT234 and PLAT342 Alerts, as well as similar Level C Alerts.

Alert level B

PLAT234_ALERT_4_B Large Hirshfeld Difference C4 --C5 . 0.30 Ang.

Author Response: This issue is also determined by the same reasons above underlined for the other type A Alerts above. Several attempts were made to model the phenyl rings of the triphenylphosphine and of the thiosemicarbazonate by introducing RIGU restraints or a combination of RIGU restraints and EADP constraints, but these strategies did not allow to reach a final model incorporating anisotropic treatment of non-hydrogen atoms. At the end, we were forced to impose also AFIX constraints to the above molecular fragments; nevertheless, the ring carbon atoms remained affected by significant thermal motions and the paucity of the data did not allow to further attenuate differences in the displacement parameters. The same applies to the remaining PLAT234 and PLAT342 Alerts, as well as similar Level C Alerts.

```
PLAT234_ALERT_4_B Large Hirshfeld Difference Re1A     --N1A      .       0.26 Ang.
```

Author Response: This issue is also determined by the same reasons above underlined for the other type A Alerts above. Several attempts were made to model the phenyl rings of the triphenylphosphine and of the thiosemicarbazonate by introducing RIGU restraints or a combination of RIGU restraints and EADP constraints, but these strategies did not allow to reach a final model incorporating anisotropic treatment of non-hydrogen atoms. At the end, we were forced to impose also AFIX constraints to the above molecular fragments; nevertheless, the ring carbon atoms remained affected by significant thermal motions and the paucity of the data did not allow to further attenuate differences in the displacement parameters. The same applies to the remaining PLAT234 and PLAT342 Alerts, as well as similar Level C Alerts.

```
PLAT234_ALERT_4_B Large Hirshfeld Difference Re1A     --N2A      .       0.26 Ang.
```

Author Response: This issue is also determined by the same reasons above underlined for the other type A Alerts above. Several attempts were made to model the phenyl rings of the triphenylphosphine and of the thiosemicarbazonate by introducing RIGU restraints or a combination of RIGU restraints and EADP constraints, but these strategies did not allow to reach a final model incorporating anisotropic treatment of non-hydrogen atoms. At the end, we were forced to impose also AFIX constraints to the above molecular fragments; nevertheless, the ring carbon atoms remained affected by significant thermal motions and the paucity of the data did not allow to further attenuate differences in the displacement parameters. The same applies to the remaining PLAT234 and PLAT342 Alerts, as well as similar Level C Alerts.

```
PLAT342_ALERT_3_B Low Bond Precision on  C-C Bonds ..............     0.0418 Ang.
```

Author Response: See comments made above for the PLAT234 Alerts.

Alert level C

```
PLAT094_ALERT_2_C Ratio of Maximum / Minimum Residual Density ....       2.94 Report
PLAT232_ALERT_2_C Hirshfeld Test Diff (M-X)  Re1      --N1      .         5.7 s.u.
PLAT234_ALERT_4_C Large Hirshfeld Difference Re1      --O1      .        0.20 Ang.
```

Author Response: This issue is also determined by the same reasons above underlined for the other type A Alerts above. Several attempts were made to model the phenyl rings of the triphenylphosphine and of the thiosemicarbazonate by introducing RIGU restraints or a combination of RIGU restraints and EADP constraints, but these strategies did not allow to reach a final model incorporating anisotropic treatment of non-hydrogen atoms. At the end, we were forced to impose also AFIX constraints to the above molecular fragments; nevertheless, the ring carbon atoms remained affected by significant thermal motions and the paucity of the data did not allow to further attenuate differences in the displacement parameters. The same applies to the remaining PLAT234 and PLAT342 Alerts, as well as similar Level C Alerts.

```
PLAT241_ALERT_2_C High   'MainMol' Ueq as Compared to Neighbors of        C12 Check
PLAT241_ALERT_2_C High   'MainMol' Ueq as Compared to Neighbors of        S1A Check
PLAT241_ALERT_2_C High   'MainMol' Ueq as Compared to Neighbors of        O2A Check
PLAT241_ALERT_2_C High   'MainMol' Ueq as Compared to Neighbors of       C12A Check
PLAT241_ALERT_2_C High   'MainMol' Ueq as Compared to Neighbors of       C25A Check
PLAT242_ALERT_2_C Low    'MainMol' Ueq as Compared to Neighbors of        C10 Check
PLAT242_ALERT_2_C Low    'MainMol' Ueq as Compared to Neighbors of        C1A Check
PLAT242_ALERT_2_C Low    'MainMol' Ueq as Compared to Neighbors of        C5A Check
PLAT242_ALERT_2_C Low    'MainMol' Ueq as Compared to Neighbors of       C10A Check
PLAT260_ALERT_2_C Large Average Ueq of Residue Including       Re1      0.111 Check
PLAT260_ALERT_2_C Large Average Ueq of Residue Including      Re1A      0.115 Check
PLAT723_ALERT_1_C Torsion Calc        2(4), Rep       0.00 Dev...       2.00 Sigma
              C13A-C14A-C15A-C10A  1_555  1_555  1_555  1_555      #  123 Check
PLAT906_ALERT_3_C Large K Value in the Analysis of Variance ......      2.845 Check
PLAT911_ALERT_3_C Missing FCF Refl Between Thmin & STh/L=    0.418         41 Report
                 6-10  2,  -5 10  2,   0-14  3,   1-14  3,  -3 11  3,   1-13  6,
                 4-13  6,   7 -5  6,  -2-14  7,   0-14  7,  -7 -6  7,  -2-14  8,
                 5-11  8,   0  5  8,   1  7  8,  -2 -2  9,   5  1  9,  -3-13 10,
                -1 -6 10,  -6  1 10,   0-14 11,   4-11 11,  -2 -3 11,   4  0 11,
                -3  4 11,   1-10 12,   2-10 12,   1 -9 12,  -2 -3 12,  -3 -2 12,
                 0  3 12,   1-11 13,   1-10 13,   2-10 13,  -2 -3 13,  -3 -2 13,
                -3  1 13,   1-11 14,  -4 -3 14,  -3 -2 14,  -2 -3 15,
```

Alert level G

```
PLAT083_ALERT_2_G SHELXL Second Parameter in WGHT  Unusually Large      25.84 Why ?
PLAT171_ALERT_4_G The CIF-Embedded .res File Contains EADP Records          2 Report
PLAT187_ALERT_4_G The CIF-Embedded .res File Contains RIGU Records          3 Report
PLAT199_ALERT_1_G Reported _cell_measurement_temperature ..... (K)        293 Check
PLAT200_ALERT_1_G Reported   _diffrn_ambient_temperature ..... (K)        293 Check
PLAT720_ALERT_4_G Number of Unusual/Non-Standard Labels .........           3 Note
              H9AA    H9AB    H9AC
PLAT860_ALERT_3_G Number of Least-Squares Restraints ............          27 Note
PLAT909_ALERT_3_G Percentage of I>2sig(I) Data at Theta(Max) Still        71% Note
```

```
PLAT910_ALERT_3_G Missing # of FCF Reflection(s) Below Theta(Min).          3 Note
                0  1  0,   0 -1  1,   0  0  1,
PLAT933_ALERT_2_G Number of HKL-OMIT Records in Embedded .res File          4 Note
                0-14  7,   0  5  8,   1-13  6,   1  7  8,
PLAT941_ALERT_3_G Average HKL Measurement Multiplicity ..........         3.3 Low
PLAT969_ALERT_5_G The 'Henn et al.' R-Factor-gap value ..........       4.280 Note
              Predicted wR2: Based on SigI**2  3.60 or SHELX Weight 13.08
PLAT978_ALERT_2_G Number C-C Bonds with Positive Residual Density.          0 Info
```

```
   3 ALERT level A = Most likely a serious problem - resolve or explain
   4 ALERT level B = A potentially serious problem, consider carefully
  17 ALERT level C = Check. Ensure it is not caused by an omission or oversight
  13 ALERT level G = General information/check it is not something unexpected

   3 ALERT type 1 CIF construction/syntax error, inconsistent or missing data
  16 ALERT type 2 Indicator that the structure model may be wrong or deficient
   9 ALERT type 3 Indicator that the structure quality may be low
   8 ALERT type 4 Improvement, methodology, query or suggestion
   1 ALERT type 5 Informative message, check
```

It is advisable to attempt to resolve as many as possible of the alerts in all categories. Often the minor alerts point to easily fixed oversights, errors and omissions in your CIF or refinement strategy, so attention to these fine details can be worthwhile. In order to resolve some of the more serious problems it may be necessary to carry out additional measurements or structure refinements. However, the purpose of your study may justify the reported deviations and the more serious of these should normally be commented upon in the discussion or experimental section of a paper or in the "special_details" fields of the CIF. checkCIF was carefully designed to identify outliers and unusual parameters, but every test has its limitations and alerts that are not important in a particular case may appear. Conversely, the absence of alerts does not guarantee there are no aspects of the results needing attention. It is up to the individual to critically assess their own results and, if necessary, seek expert advice.

Publication of your CIF in IUCr journals

A basic structural check has been run on your CIF. These basic checks will be run on all CIFs submitted for publication in IUCr journals (*Acta Crystallographica*, *Journal of Applied Crystallography*, *Journal of Synchrotron Radiation*); however, if you intend to submit to *Acta Crystallographica Section C* or *E* or *IUCrData*, you should make sure that full publication checks are run on the final version of your CIF prior to submission.

Publication of your CIF in other journals

Please refer to the *Notes for Authors* of the relevant journal for any special instructions relating to CIF submission.

PLATON version of 02/02/2025; check.def file version of 02/02/2025

Datablock 32_xx1_twin_nowob_nofried_twin1_hklf4_088 - ellipsoid plot

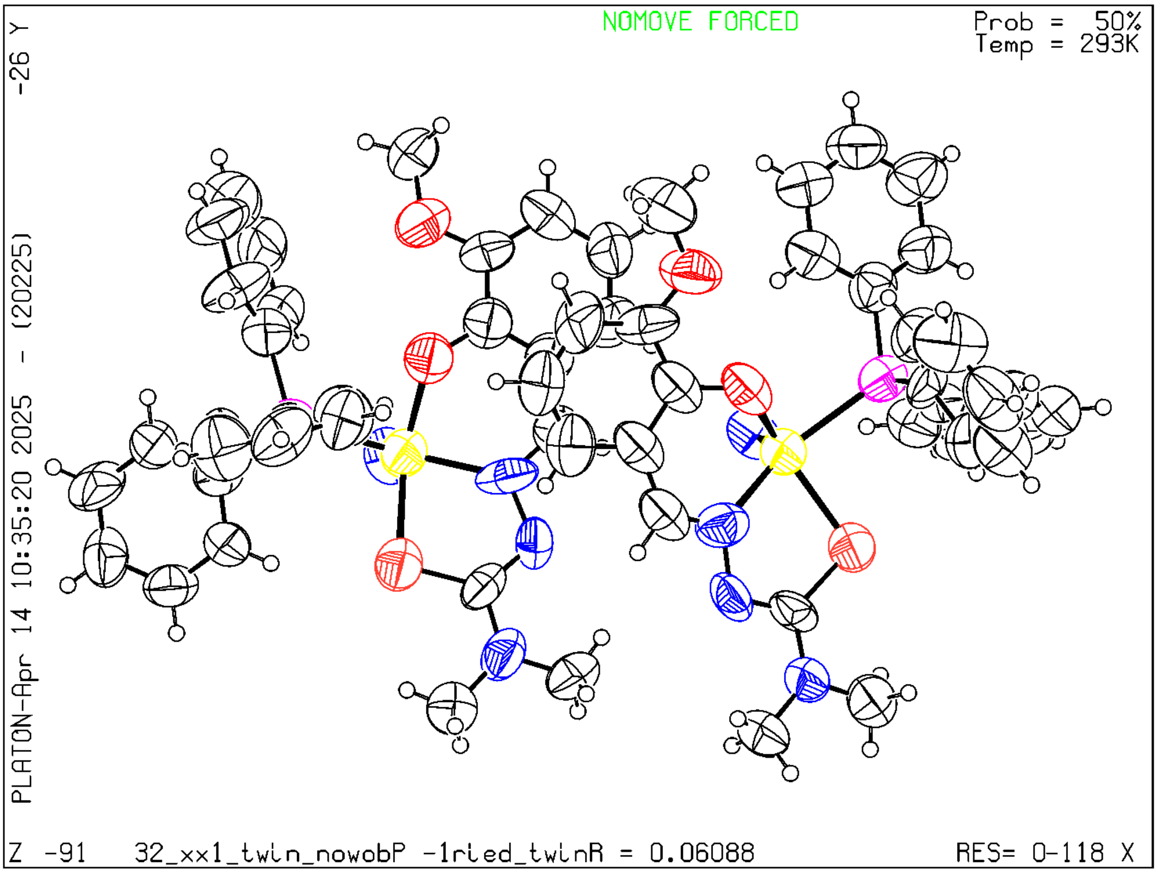

Supplement: Supplementary file 1 — Supporting Information Supporting Information is available as separate files. This information provides additional data related to the study presented in the main manuscript, essential for providing further evidence for the chemical identities of the obtained compounds and supporting the conclusions. It includes a detailed pdf file containing the following material: ESI–MS, FT‐IR, one‐dimensional 1H, 13C, 31P NMR and two‐dimensional NMR spectra of rhenium complexes Re0, Re1 and Re2; crystallographic data and diagrams for H 2 L2 and rhenium complexes Re1 and Re2; radio/UV‐HPLC and LC–MS data for 99g/99m Tc1-3; stability of 99m Tc1-4 in phosphate buffer saline, cysteine 1 mM, glutathione 1 mM and human serum type AB; experimental details of the attempts to obtain PCN‐based rhenium complexes. Crystallographic data in the form of .cif files (file names: ‘32_xx1_twin_nowob_nofried_twin1_hklf4_088.cif’, ‘33_DC_auto.cif’ and ‘test_mr24fp.cif’), and checkcif as PDF files (file names: ‘32_xx1_twin_nowob_nofried_twin1_hklf4_088 cifreport.pdf’, ‘33_DC_auto_cifreport.pdf’ and ‘test_mr24fp_cifreport.pdf’), are also included. [file 32_xx1_twin_nowob_nofried_twin1_hklf4_088 cifreport.pdf › 32_xx1_twin_nowob_nofried_twin1_hklf4_088 cifreport.pdf]
